# Curcumin‐Enabled Metal‐Free Photocatalytic Oxidation of Aryl‐ and Alkyl‐methanamines to Imines Under Green Conditions

**DOI:** 10.1002/cssc.70980

**Published:** 2026-08-18

**Authors:** Tommaso Castiglia, Maria Angela Chiarella, Andrea Shati, Andrea Aloia, Stefano Savino, Cosimo Annese, Angelo Nacci, Antonio Monopoli

**Affiliations:** ^1^ Dipartimento di Chimica Università degli Studi di Bari Aldo Moro Bari Italy; ^2^ Department of Chemistry University of Elbasan ‘Aleksandër Xhuvani’ Elbasan Albania; ^3^ ICCOM‐CNR SS Bari Bari Italy; ^4^ Department of Life Sciences Health and Health Professions Università degli Studi Link Roma Italy

**Keywords:** aerobic oxidation, curcumin, imine synthesis, photocatalysis, sustainable chemistry

## Abstract

Herein, we report a fully bio‐based, metal‐free photocatalytic protocol for the aerobic oxidation of aryl‐ and alkyl‐methanamines to imines under blue‐light irradiation. This transformation leverages curcumin, a naturally abundant and renewable polyphenol, as an efficient visible‐light photosensitizer and 2‐methyltetrahydrofuran (2‐MeTHF), a biomass‐derived solvent, establishing a sustainable catalytic system composed entirely of organic, bio‐derived components. The reaction proceeds efficiently at room temperature using molecular oxygen as the sole terminal oxidant, strictly avoiding transition metals, stoichiometric oxidants, and hazardous additives. A broad range of benzylic and aliphatic amines is converted to the corresponding imines in good to excellent yields (up to 98%), demonstrating the generality and practicality of the protocol. Comprehensive mechanistic investigations—including light on/off experiments, oxygen‐dependence studies, radical trapping, Stern–Volmer fluorescence quenching studies and Hammett analysis—support a sensitizer‐controlled aerobic photocatalytic pathway involving photoinduced electron transfer and oxygen‐mediated catalyst regeneration via superoxide radical anions. This work highlights the efficacy of natural pigments in driving high‐efficiency, sustainable organic transformations.

## Graphical abstract

Curcumin, a natural polyphenol, enables a fully bio‐based and metal‐free photocatalytic system for the aerobic oxidation of aryl‐ and alkyl‐methanamines to imines under blue light. Using 2‐methyltetrahydrofuran (2‐MeTHF) as a renewable solvent and molecular oxygen as the sole oxidant, the protocol affords up to 98% yield. Mechanistic studies support a photoinduced single‐electron transfer pathway via superoxide.

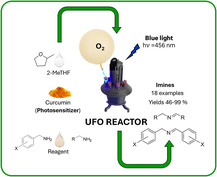

## 1 Introduction

The imine (C═N) functional group is a central structural motif in organic synthesis, materials science, and chemical biology, owing to its versatility and dynamic bonding characteristics. Imines serve as key intermediates in the synthesis of amines, heterocycles, and fine chemicals, particularly through reductive amination and multicomponent reactions that are widely applied in both academic and industrial settings [1, 2]. In medicinal and pharmaceutical chemistry, imine‐containing compounds and Schiff base derivatives have attracted sustained interest due to their broad spectrum of biological activities and their role as adaptable pharmacophores [3, 4]. From a sustainability perspective, imines are also pivotal in coordination chemistry, where Schiff base ligands enable the design of efficient catalysts based on earth‐abundant metals [5]. Moreover, the reversible nature of the C─N bond has been extensively exploited in dynamic covalent chemistry, underpinning the development of self‐healing polymers, adaptive networks, and covalent organic frameworks (COFs) with applications in gas storage, catalysis, and energy‐related materials [6, 7]. Given the widespread utility of imines and their relevance to sustainable molecular design, the development of green, metal‐free, and energy‐efficient strategies for imine synthesis remains an important objective within contemporary sustainable chemistry.

(I) $$2{\mathrm{ArCH}}_{2}{\mathrm{NH}}_{2}\;\overset{\left[\mathrm{OX}\right]}{\to }\;{\mathrm{ArCH}}_{2}N═\mathrm{CHAr}+{\mathrm{NH}}_{3}+H_{2}$$

Classical methods for the synthesis of imines via homocoupling of arylamines (Equation (1)) typically rely on oxidative conditions using stoichiometric amounts of chemical oxidants or transition‐metal catalysts. Common systems employ noble or base metals such as Pd, Cu, Ru, or Mn in combination with oxidants, including molecular oxygen, peroxides, or hypervalent iodine reagents [8, 9, 10, 11, 12, 13]. While effective, these approaches often suffer from environmental drawbacks, including metal contamination, poor atom economy, hazardous waste generation, and the need for elevated temperatures or organic solvents. In addition, the use of nonrenewable catalysts and external oxidants raises sustainability concerns, particularly for large‐scale applications. These limitations have motivated the development of greener, metal‐free, and energy‐efficient alternatives for imine synthesis [2, 14].

Modern advancements have identified COFs as a transformative platform for addressing these sustainability challenges. Due to their high surface area, tunable porosity, and predesignable active sites, COFs have emerged as superior metal‐free heterogeneous catalysts for imine synthesis [15, 16]. These frameworks allow for the precise integration of photoactive or electroactive moieties, facilitating efficient amine homocoupling under ambient conditions and ensuring high chemical stability and recyclability, bridging the gap between high‐performance catalysis and the requirements of green chemistry [17].

**FIGURE 1:**
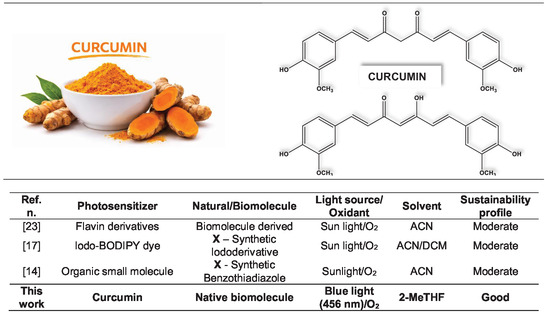
Upper panel: curcumin. Lower panel: qualitative comparative analysis between this work and some previous studies.

Recent green methods for arylamine homocoupling employ electrochemical oxidation, or oxygen as a benign oxidant, often under metal‐free conditions and ambient temperatures, minimizing waste and energy use [14, 18, 19]. Among these methods, photocatalysis has emerged as a powerful green strategy in organic synthesis, enabling chemical transformations under mild conditions using light as a clean energy source [20]. By avoiding stoichiometric oxidants, photocatalytic processes reduce waste, energy consumption, and environmental impact, aligning with the principles of sustainable chemistry [21, 22, 23, 24]. They also enable the use of renewable and bio‐derived, visible or UV‐light‐absorbing photocatalysts, further enhancing eco‐friendliness, like flavonoids, riboflavin, chlorophyl, porphyrins and curcumin, just to cite a few [2, 25, 26, 27]. This latter, a naturally occurring polyphenolic dye isolated from *Curcuma longa* that exhibits a strong absorption band in the visible region (*λ*_max_ ≈ 420–430 nm in organic solvents), attributable to its extended π‐conjugated β‐diketone framework and donor–acceptor electronic structure [28, 29], has recently attracted attention as an eco‐friendly photocatalyst. Its strong absorption in the visible region and photostability allow it to drive organic transformations under mild conditions, reducing reliance on toxic photosensitizers and harsh oxidants (Figure 1) [30, 31, 32].

Despite the increasing use of curcumin as a natural and sustainable photosensitizer in photochemical and photocatalytic applications, its synthetic scope remains limited. It is widely regarded as an ideal natural photosensitizer for applications ranging from antimicrobial therapy to cancer treatment because its primary absorption band aligns almost perfectly with standard blue light sources. Curcumin has been explored primarily in photodynamic processes, photodegradation, and selected organic transformations owing to its strong visible‐light absorption and photochemical activity [33, 34]. However, to the best of our knowledge, its application in the photocatalytic oxidation of amines into imines has not been reported to date.

In 2014, some of us reported the use of ionic liquids in the metal‐free oxidative homocoupling of amines to yield imines with very good yields. The high efficiency observed in ionic liquids was mechanistically attributed to enhanced activation of both benzylamine and molecular oxygen through an initial electron‐transfer step, facilitated by the ionic character of the solvent [35]. Following our interest in the development of greener protocols in organic synthesis [36, 37, 38], we report here a new protocol for the oxidation of amines under metal‐free photocatalytic conditions, utilizing a natural‐derived photosensitizer and a biomass‐derived solvent, at room temperature.

## 2 Results and Discussion

Optimization of the reaction conditions was started with the solvent scope summarized in Table 1. The screening was conducted using 4‐fluorobenzylamine (**1**) as a model substrate in a custom‐3D‐printed reactor (UFO reactor, Figure S1) [39]. Reactions were initially performed using 1,2,3,5‐tetrakis(carbazol‐9‐yl)‐4,6‐dicyanobenzene (4‐CzIPN, 5 mol%) as the photosensitizer, selected based on its recent application by some of us [40], and irradiated with a blue lamp (456 nm) as the light source. To manage the heat generated by lamp use, the system was continuously cooled by a fan positioned beneath the UFO reactor, maintaining a reaction temperature of approximately 22–26 °C. Gases such as O_2_ or N_2_ were supplied by a balloon at atmospheric pressure connected to the vial (see Supplementary Material).

**TABLE 1:** Solvent scope in the oxidative homocoupling of 4‐fluorobenzylamine.

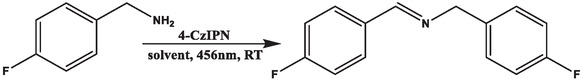					
Entry^a^	Solvent	Atmosphere	*t*, h	Conversion^b^, %	Selectivity^b^, %
1^c^	CH_3_CN	Air	20	5	98
2^c^	CH_3_CN	O_2_	20	8	97
3	CH_3_CN	Air	20	51	94
4	CH_3_CN	O_2_	4	64	95
5	THF	O_2_	4	59	85
6	1,1,1,3,3,3‐Exafluoro‐2‐propanol	O_2_	4	29	99
7	Isopropanol	O_2_	4	36	98
8	2‐Me‐THF	O_2_	2	98	99
9	2‐Me‐THF	N_2_	4	5	73

a General conditions: solvent 1 mL, blue‐light (100%), 4‐CzIPN (5 mol%), 0.5 mmol of 4‐fluorobenzylamine, O_2_ at atmospheric pressure, in a gas‐tight vial at room temperature.

b Conversions and selectivity were determined by GC–MS analyses, with anisole as the internal standard. All results represent the average of at least two experiments.

c Reaction performed without photosensitizer.

Based on the data presented in Table 1, both the photosensitizer, the solvent, and the reaction atmosphere exert a pronounced influence on the efficiency of the visible‐light‐driven homocoupling of 4‐fluorobenzylamine. In the absence of a photosensitizer, only negligible conversion was observed in acetonitrile, irrespective of the atmosphere (entries 1, 2), confirming that direct aerobic oxidation is inefficient under these conditions. Introduction of 4‐CzIPN enabled productive photoredox turnover, with molecular oxygen significantly accelerating the reaction (entry 4 vs. entry 3). The effects of solvents were substantial: protic solvents and fluoroalcohol resulted in lower reaction conversions (entries 6, 7), likely due to intermolecular hydrogen‐bonding interactions with the medium. Such interactions may interfere with the formation and stabilization of the enolic form of curcumin, thereby perturbing its photophysical properties and reducing catalytic efficiency [41]. In contrast, 2‐methyltetrahydrofuran (2‐MeTHF) afforded near‐quantitative conversion with excellent selectivity (entry 8). Importantly, beyond its favorable mechanistic role, 2‐MeTHF represents a sustainable solvent choice, being derived from renewable biomass, exhibiting low toxicity, and offering improved safety and environmental profiles compared to conventional ethers, thereby reinforcing the overall greenness of the developed photocatalytic protocol [42]. The loss of activity under air (entry 3) and especially with N_2_ (entry 9) confirms the essential role of molecular oxygen as a terminal oxidant in the catalytic cycle, suggesting an involvement in the rate‐determining step (vide infra).

Then, the effect of the photosensitizer on the homocoupling efficiency was systematically investigated (Table 2).

**TABLE 2:** Photosensitizer scope in the oxidative homocoupling of 4‐fluorobenzylamine.

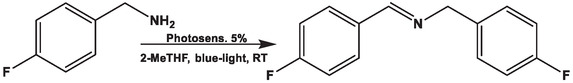				
Entry^a^	Photosensitizer	*t*, h	Conversion^b^, %	Selectivity^b^, %
1	4‐CzIPN	2	99	99
2	Lumazine	4	18	97
3	9,10‐Diphenylanthracene	4	48	99
4	9‐Bromoanthracene	4	26	99
5	9‐Methylanthracene	4	48	95
6	2,7‐Dibromofluorene	4	53	99
7	Curcumin	4	91	95
8	Curcumin	2	66	94
9	Curcumin	3	90	95
10	Curcumin (10%)	2	36	90
11	Curcumin (10%)	4	93	87
12	Curcumin (1%)	4	49	96
13	Curcumin (dark)	3	8	96
14	Curcumin (370 nm)	3	53	94
15	Curcumin (white lamp)	3	27	99

a General conditions: 4‐fluorobenzylamine: 0.5 mmol; photosensitizer: 5% mol; 1 mL of 2‐MeTHF as the solvent; blue‐light (100%) O_2_ at atmospheric pressure, at room temperature.

b Conversions and selectivity were determined by GC–MS analyses, with anisole as the internal standard. All results represent the average of at least two experiments.

As already shown, the benchmark photocatalyst 4‐CzIPN delivers quantitative conversion within 2 h (Table 2, entry 1), confirming its high photoactivity [43], albeit at the expense of a synthetically complex and less sustainable chromophore. In contrast, classical organic photosensitizers such as lumazine [44] and anthracene‐ [45] or fluorene‐based [46] derivatives (entries 2–6) afford only moderate conversions even after prolonged irradiation while maintaining excellent selectivity.

Notably, under these conditions, curcumin displayed a markedly enhanced activity profile, affording 91% conversion after 4 h and reaching comparable conversion within 3 h (entries 7–9), while maintaining high selectivity. Surprisingly, increasing the curcumin loading to 10 mol% resulted in a slower initial conversion (entry 10), possibly due to inner‐filter effects or increased excited‐state quenching at higher chromophore concentrations. Similarly, comparable results were obtained by reducing the curcumin content to 1% (entry 12). Control experiments confirm the essential role of light and wavelength matching: negligible conversion occurs in the dark (entry 13), whereas irradiation at 370 nm or with a white lamp (entries 14–15) yielded lower conversions, consistent with suboptimal overlap between the light source and curcumin’s absorption profile [33]. In fact, the UV–Vis absorption profile of curcumin in 2‐MeTHF shows a maximum at 424 nm (see Figure S2).

The use of curcumin instead of 4‐CzIPN, effectively addresses the high cost and synthetic complexity associated with established dyes, offering a biodegradable and inexpensive alternative that fulfills the requirements of green chemistry without compromising reactivity. Furthermore, as already stated in the manuscript, this represents the first application of this naturally abundant polyphenol for this specific transformation.

With the optimized reaction conditions in hand (2‐MeTHF, curcumin, O_2_ at atmospheric pressure), the substrate scope was explored to evaluate the generality of the oxidative homocoupling and to gain insight into the structural parameters governing reactivity. A range of mono‐ and di‐substituted benzylamines, α‐naphthylmethanamine, and representative alkylamines were examined in order to probe the influence of benzylic activation, electronic effects, and steric demand. For each substrate, the reaction time was adjusted to maximize conversion while minimizing irradiation time. The results are collected in Table 3. Overall, the protocol exhibits broad applicability, while clearly revealing the impact of electronic, steric, and structural features on the efficiency of imine formation. Benzylic amines generally undergo smooth oxidative homocoupling, affording the corresponding imines in good to excellent yields (80%–98%) within moderate reaction times (3–6 h). Both electron‐donating (4‐OMe, 4‐Me) and electron‐withdrawing substituents (4‐F, 4‐‍Cl, 4‐CF_3_) on the aromatic ring are well tolerated, indicating that the transformation is not limited to highly electron‐rich substrates (Table 3, entries **1**–**5**). Notably, α‐methylbenzylamine furnishes the homocoupled imine in nearly quantitative yield (98%, **6**). The enhanced efficiency relative to unsubstituted benzylamine is consistent with increased stabilization of α‐radical and/or iminium intermediates arising from secondary α‐substitution. In contrast, sterically congested substrates such as 1,1‐diphenylmethanamine display reduced efficiency (65%, **9**), which could be plausibly attributed to steric inhibition of iminium formation or subsequent C─N bond construction [9, 47].

**TABLE 3:** Scope of the reaction.^a^

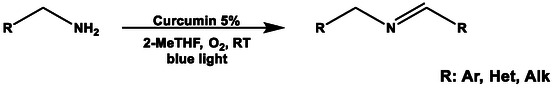		
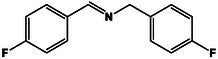 1, 3 h, 90%^b^	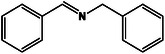 2, 5 h, 80%	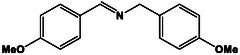 3, 6 h, 93%
		
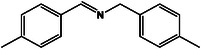 4, 4 h, 93%	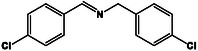 5, 3 h, 84%	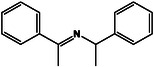 6, 6 h, 98%f
		
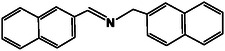 7, 2 h, 75%	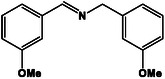 8, 4 h, 98%	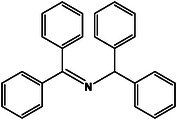 9, 2 h, 65%
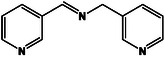 10, 2 h, 91%	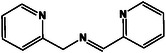 11, 6 h, 33%	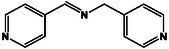 12, 4 h, 59%
		
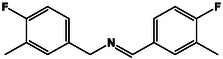 13^c^, 4 h, 84%	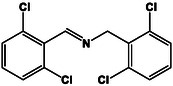 14, 4 h, 19%	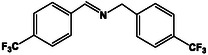 15, 4 h, 95%
		
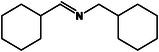 16, 3 h, 84%	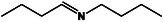 17, 2 h, 90%	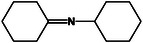 18, 6 h, traces

a General conditions: 2‐MeTHF 1 mL, blue‐light (100% of the power), curcumin (5% mol), 0.5 mmol of amine, O_2_ at atmospheric pressure, in a gas‐tight vial at room temperature.

b Yields were determined by GC–MS analyses, with anisole as the internal standard. All results represent the average of at least two experiments.

c Isolated yield.

Similarly, α‐naphthylmethanamine undergoes efficient oxidative homocoupling, albeit with a slightly diminished yield (75%, **7**), in line with its increased steric bulk. A pronounced decrease in efficiency is observed for 2,6‐dichlorobenzylamine (19%, **14**), highlighting the combined influence of strong electron‐withdrawing substituents and steric effects, which may disfavor both the initial photoinduced oxidation and the subsequent coupling step [48]. *Meta*‐substituted benzylamines, exemplified by 3‐methoxybenzylamine, also afford excellent yields, further underscoring the robustness of the protocol (**8**).

The reactivity of picolylamines displays a marked positional dependence. While 3‐ and 4‐picolylamine provide the desired imines in high yields (91%–59%, **10,** and **12**), 2‐picolylamine is substantially less reactive (33%, **11**). This reduced conversion may reflect steric and/or electronic effects associated with the proximal pyridine nitrogen, potentially leading to inefficient electron transfer or nonproductive quenching of the excited photosensitizer. Notably, while picolylamines are influenced by these coordinating effects, noncoordinating substrates like methoxybenzylamines follow a trend dictated primarily by electronic factors. In these cases, the inductive electron‐withdrawing nature of the *meta*‐substitution appears slightly more favorable for conversion (98%) than the resonance‐donating *para*‐substitution (93%), in line with our Hammet results (vide infra).

Importantly, the method is not restricted to aromatic substrates. Primary aliphatic amines such as *n*‐butylamine and cyclohexylmethanamine undergo efficient oxidative homocoupling, affording the corresponding imines in 84%–90% yield (**16** and **17**).

These results demonstrate that benzylic activation is not strictly required, provided that α‐C─H bonds adjacent to nitrogen are accessible for oxidation. In contrast, cyclohexylamine, which lacks an activated α‐position and forms a less stable iminium intermediate, affords only trace amounts of product even after prolonged irradiation (**18**), emphasizing the crucial role of α─C─H activation and iminium stabilization in the homocoupling process.

The observed selectivity remains remarkably high across nearly all tested substrates; however, small amounts of the corresponding aldehyde are occasionally detected, particularly at prolonged reaction times. Although this side product could, in principle, arise from amine over‐oxidation, oxidative imine cleavage, or an oxygenative pathway operating instead of the dehydrogenative process (vide infra), its effective suppression upon addition of MgSO_4_ (data not shown) points to a moisture‐dependent hydrolysis pathway as the most plausible explanation, consistent with the in situ generation of water during imine formation. While the addition of MgSO_4_ can further suppress traces of imine hydrolysis, it was excluded from the general protocol to maintain a minimalist, additive‐free procedure in line with green chemistry principles, as selectivity remained high even in its absence.

### 2.1 Mechanistic Insights and Catalytic Cycle

To gain further mechanistic insight, a series of control experiments was performed.

**FIGURE 2:**
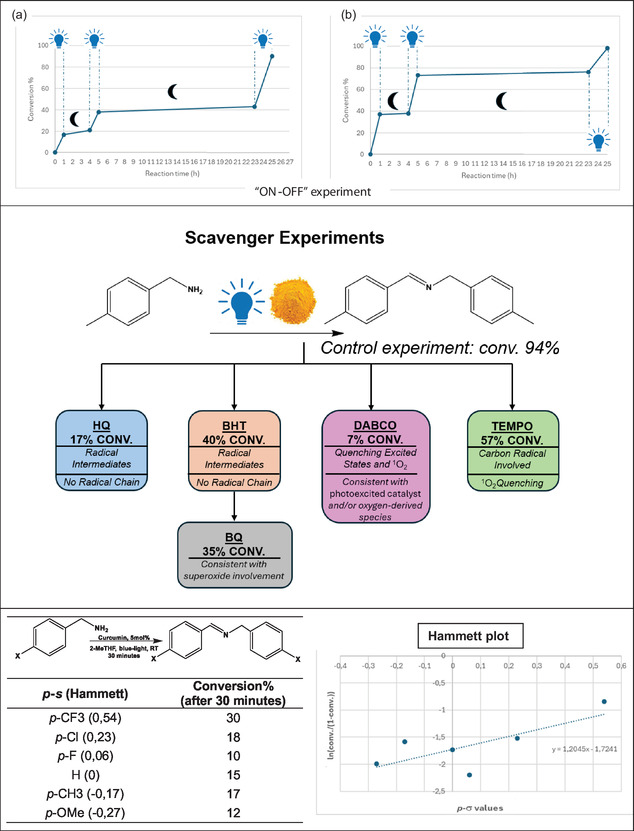
Upper panel: “ON–OFF” experiment. (a) Homocoupling of 4‐methylbenzylamine. (b) Homocoupling of 4‐fluorobenzylamine. Middle panel: scavenger experiment with 4‐methylbenzylamine under standard conditions. Lower panel: Hammet studies.

First, a light on/off experiment was conducted with 4‐methylbenzylamine under the standard reaction conditions to probe the photo‐dependence of the oxidation process. Such intermittency experiments are well established in photocatalysis and serve to distinguish genuinely photocatalytic pathways from thermal background reactions or chain‐propagated processes [49]. To exclude any influence of the substituent, the experiment was repeated using 4‐fluorobenzylamine under identical conditions (Figure 2, upper panel). In the case of 4‐methylbenzylamine (Figure 2, upper panel a), during the initial 1 h of irradiation, the reaction reached 17% conversion. When the light source was switched off for 3 h, only a marginal increase in conversion was observed (21%), indicating negligible background reactivity in the absence of photoexcitation. Upon reirradiation for an additional hour, the conversion increased to 38%. A subsequent dark period of 18 h resulted in no significant further progress (about 41% conversion), whereas renewed irradiation for 2 h led to a pronounced increase in conversion, reaching 90%. An analogous light‐dependent behavior was observed for the homocoupling of 4‐fluorobenzylamine (panel b), further supporting a strictly photo‐driven process. This pronounced dependence on continuous light input demonstrates that sustained photoexcitation is required for efficient reaction progression and that the transformation does not proceed via a self‐propagating radical chain mechanism. The small increases in conversion observed during dark periods are negligible and likely attributable to the slow consumption of reactive intermediates generated during prior irradiation rather than to an autonomous radical process. Overall, these results are consistent with a stepwise photoinduced electron‐transfer mechanism involving repeated excitation of curcumin, in line with other aerobic, metal‐free visible‐light oxidation processes [50].

Although curcumin is known to undergo photochemical transformation under prolonged irradiation, the light on/off experiments demonstrate that the photocatalytic system remains active throughout the reaction, indicating that such transformations do not measurably compromise catalytic performance under the applied conditions. Consistent with this observation, 1‐H NMR analysis of the crude reaction mixture after completion of the reaction revealed only weak, unresolved resonances attributable to trace catalyst‐derived species, with no evidence for a single dominant degradation product. This spectral profile is consistent with the formation of multiple low‐abundance phototransformation products, as often observed for naturally derived chromophores under prolonged irradiation**.**

Moreover, while intermittent irradiation experiments demonstrate that catalytic turnover occurs exclusively during periods of blue‐light exposure and ceases immediately in the dark, the oxygen‐dependence studies reveal that light alone is insufficient to sustain the reaction in the absence of O_2_. In air, only moderate conversion of 4‐fluorobenzylamine is observed even after extended irradiation (49% after 20 h, Table S1 entry 1), whereas under an inert nitrogen atmosphere, the reaction is effectively shut down, affording only trace conversion (8% after 20 h, Table S1, entry 2). Together, these observations indicate that both continuous photoexcitation and the presence of molecular oxygen are simultaneously required for productive catalysis. This dual dependence strongly supports a photocatalytic mechanism in which the excited curcumin photosensitizer initiates substrate oxidation under illumination, while oxygen serves as the terminal oxidant, enabling catalyst regeneration. The absence of significant conversion during dark periods or under oxygen‐free conditions rules out thermally driven pathways and self‐propagating radical chains, reinforcing the interpretation of a strictly light‐gated, oxygen‐driven catalytic cycle operating under ambient and sustainable conditions. Furthermore, the sharp increase in conversion upon reirradiation—even after 23 h in the presence of dissolved oxygen and air, with all reagents remaining in solution—‍demonstrates the robustness and sustained photoresponsiveness of the system under the applied conditions.

Then, to identify the reactive intermediates involved in the photocatalytic cycle, a series of chemical scavenger experiments was performed using the oxidative homocoupling of 4‐methylbenzylamine as the benchmark reaction (94% conversion after 4 h; Table 3, compound 4). As shown in Figure 2 (middle panel), the addition of classical radical scavengers substantially suppressed the reaction. Specifically, hydroquinone (HQ) and 3,5‐di‐*tert*‐butyl‐4‐hydroxytoluene (BHT) decreased the conversion to 17% and 40%, respectively. This pronounced, yet incomplete, inhibition is consistent with the involvement of radical intermediates while arguing against a self‐sustaining radical chain mechanism. To obtain further insight into the nature of these intermediates, additional scavenger experiments were performed. The addition of benzoquinone (BQ), a well‐established scavenger of superoxide radical anions, reduced the conversion to 35%, which is consistent with the participation of O_2_^⋅−^in the catalytic cycle. In contrast, TEMPO produced a more moderate inhibition (57% conversion), suggesting the transient interception of carbon‐centered benzylic radical intermediates without completely interrupting the catalytic turnover. Finally, the addition of 1,4‐diazabicyclo [2.2.2]octane (DABCO) almost completely suppressed the reaction (7%–9% conversion). Since DABCO is an efficient quencher of both singlet oxygen (^1^O_2_) [51, 52] and photoexcited catalyst states [53], this marked inhibition indicates that productive catalysis requires photoexcitation and is consistent with the involvement of photoexcited curcumin and/or oxygen‐derived excited species. Although this experiment does not distinguish between these two quenching pathways, it clearly highlights the essential role of continuous photoexcitation in the catalytic process.

Additionally, the influence of aromatic substituents on the initial stages of the oxidative homocoupling was further examined through a Hammett analysis based on conversions measured after 30 min. A clear dependence on the para‐Hammett constant (*σ*_p_) is observed, with electron‐withdrawing substituents promoting higher early‐stage conversion. Specifically, substrates bearing strongly electron‐withdrawing groups such as ─CF_3_ (*σ*_p_ = +0.54) and ─Cl (*σ*_p_ = +0.23) exhibit significantly enhanced reactivity (30%–18% conversion) relative to the parent compound (*σ*_p_ = 0) and electron‐rich substrates such as *p*‐OMe derivative (12%).

### 2.2 Hammett Plot

This trend, characterized by a positive reaction constant (*ρ *> 0), suggests that the initial photoinduced single‐electron transfer (SET) is unlikely to be the sole turnover‐limiting event. Rather, one or more downstream chemical transformations contribute significantly to overall rate control. Instead, the data are consistent with a downstream rate‐determining step involving the loss of positive charge at the nitrogen or the development of radical character at the benzylic position. In particular, the α‐deprotonation of the amine radical cation would be electronically facilitated by electron‐withdrawing substituents, which enhance benzylic C─H acidity and stabilize the transition state leading to the neutral benzylic radical [54, 55]. Linearization of the data using ln[conv/(1–conv)] reveals a systematic, though not perfectly linear, dependence on *σ*_p_, characteristic of charge‐controlled processes embedded within multistep catalytic manifolds [56].

The deviations from ideal linearity, including the slight V‐shaped trend observed for electron‐donating substituents (Me, OMe), suggest that multiple elementary steps contribute to overall rate control within the photocatalytic cycle rather than a single, well‐defined rate‐determining electron‐transfer event. This behavior aligns well with light on/off and oxygen‐dependence experiments, demonstrating that catalytic turnover is gated by continuous photoexcitation and efficient oxygen‐mediated catalyst regeneration rather than by the intrinsic oxidation potential of the substrate alone.

**FIGURE 3:**
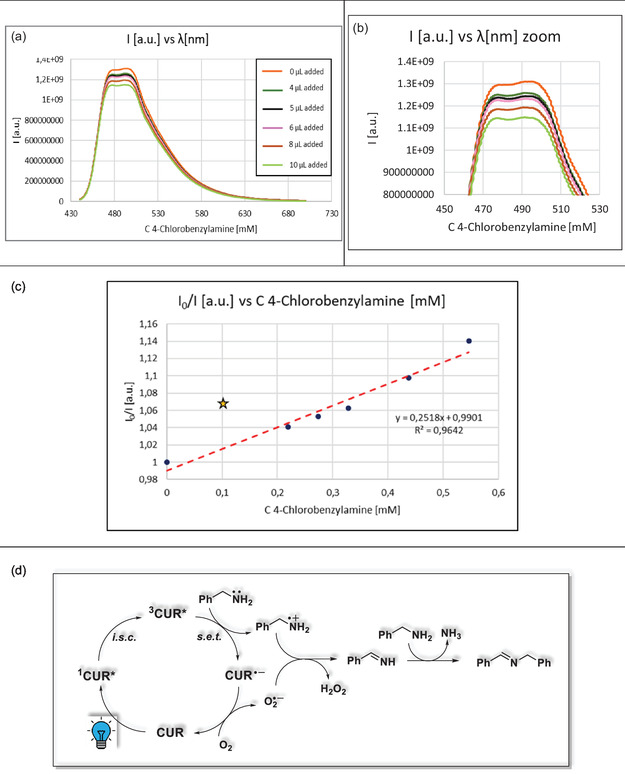
Upper panel: (a) emission spectra of curcumin in 2‐MeTHF with incremental additions of 4‐chlorobenzylamine; (b) magnification of the emission peak. Middle panel: (c) Stern–Volmer plot (*I*°/*I* vs. quencher concentration). The star point indicates a curcumin‐to‐quencher ratio of approximately 10% mol, representing a deviation from the linear quenching regime. Lower panel: (d) plausible pathway for the catalytic cycle.

Finally, to provide a quantitative basis for the proposed mechanism and verify the primary photochemical event, we conducted Stern–Volmer fluorescence quenching studies of curcumin in 2‐MeTHF using 4‐chlorobenzylamine as the quencher (Figure 3). The emission intensity of curcumin decreased systematically upon incremental additions of the amine, yielding a linear Stern–Volmer plot (*R* ^2^ = 0.9642) across the investigated concentration range (0–0.55 mM). The calculated quenching constant (*K*_sv_ = 0.2518 mM^−1^) is consistent with an efficient interaction between the excited state of the photocatalyst and the substrate. This robust quenching corroborates the initiation of the catalytic cycle via interception of photoexcited curcumin by the amine, likely through a photoinduced electron transfer (PET) process [57]. Furthermore, the near‐ideal intercept of 0.9901 supports the reliability of the fluorescence data and is consistent with a sensitizer‐controlled initiation step, providing experimental validation of the primary event in the photocatalytic manifold. Interestingly, while the quenching of curcumin by 4‍‐‍chlorobenzylamine follows a linear trend at low concentrations, a significant deviation (an outlier, the star point in the diagram) from the Stern–Volmer relationship occurs as the catalyst‐to‍‐‍quencher ratio approaches 10% (molar ratio). This nonlinear behavior suggests that at higher relative chromophore densities, nonproductive pathways—such as catalyst self‐quenching or the formation of nonreactive ground‐state complexes—compete with productive substrate oxidation. These findings provide a consistent explanation for the lower yields obtained in the homocoupling of 4‐fluorobenzylamine at high loadings, where the interference of excess photocatalyst likely outweighs the benefits of increased light absorption, identifying a critical concentration threshold for optimal catalytic turnover (Table 2, entry 11).

Taken together, the Hammett data support a mechanism where, following the initial photo‐initiation, downstream chemical steps—most plausibly involving benzylic C─H bond cleavage and/or radical intermediate formation—exert primary kinetic influence over the early stages of the reaction. The observed positive reaction constant (*ρ* > 0), therefore supports electronic stabilization of the transition state during these oxidation steps, but does not imply a simple outer‐sphere electron‐transfer equilibrium as the sole turnover‐limiting event. Furthermore, the absence of a steep or ideal linear Hammett correlation argues against a simple, outer‐sphere electron‐transfer equilibrium; instead, it supports a sensitizer‐controlled, multistep photocatalytic pathway in which oxygen‐mediated catalyst regeneration and iminium interception contribute significantly to overall efficiency. This interpretation is further supported by Stern–Volmer quenching studies using 4‐chlorobenzylamine, which experimentally verify the efficient interception of the excited curcumin by the amine substrate as the primary photochemical event. This is fully consistent with the additive studies and the strictly light‐gated behavior observed experimentally.

Based on the collective experimental evidence, a sensitizer‐controlled aerobic photocatalytic manifold is proposed (Figure 3, panel d). Upon blue‐light irradiation (456 nm), curcumin is excited to its singlet state and undergoes intersystem crossing to the corresponding triplet state (^3^CUR*), which serves as the primary catalytically active species [29]. The excited photosensitizer plausibly undergoes SET with the amine substrate, generating an aminium radical cation and the curcumin radical anion [56]. Subsequent electron transfer from the reduced catalyst to molecular oxygen regenerates the ground‐state sensitizer and furnishes the superoxide radical anion (O_2_^•−^), consistent with the pronounced suppression of reactivity observed in the presence of benzoquinone [55].

Although energy transfer to oxygen to produce singlet oxygen (^1^O_2_) cannot be fully excluded—particularly in light of DABCO quenching—the absolute requirement for oxygen and the absence of characteristic oxidative C─N cleavage products support a predominantly SET/superoxide‐driven pathway under the present conditions [58, 59].

Oxidation of the amine substrate proceeds through reactive radical intermediates. Hammett analysis reveals a positive reaction constant (*ρ* > 0), indicating that electron‐withdrawing substituents accelerate the early stages of the reaction. This trend is consistent with a kinetically influential step involving development of radical character accompanied by attenuation of positive charge at the benzylic position, most plausibly during α‐C─H bond cleavage of the aminium radical cation [60]. The data, therefore, suggest that C─H activation, plausibly assisted by superoxide or other oxygen‐derived species, contributes significantly to rate modulation, while the initial photoinduced SET event is unlikely to be solely rate‐determining [61].

Further support for this mechanistic picture arises from the reactivity of dibenzylamine (Table S2, entry a), which under standard conditions exclusively affords *N*‐benzylidenebenzylamine without detectable benzaldehyde formation, ruling out the possibility of an oxygenative pathway [62]. In classical ^1^O_2_ pathways, oxidative C─N cleavage of secondary amines typically yields aldehydes via hydrolytic fragmentation; thus, the observed selectivity strongly favors a SET/superoxide manifold over a dominant ^1^O_2_ cleavage pathway [63]. Finally, to evaluate the scalability of the protocol, a tenfold scale‐up was performed using 5 mmol of 4‐methylbenzylamine. After 6 h, the corresponding homocoupled imine was isolated in 91% yield, demonstrating the practical applicability of the method (Table S2, entry b).

## 3 Conclusion

In conclusion, we have developed a sustainable, metal‐free photocatalytic protocol for the oxidative homocoupling of aryl‐ and alkyl‐methanamines to imines, enabled by blue‐light irradiation and the natural photosensitizer curcumin. The method operates under exceptionally mild conditions at room temperature, employs molecular oxygen as the sole terminal oxidant, and avoids transition metals, stoichiometric oxidants, and hazardous additives. The use of curcumin—a renewable polyphenol extracted from *Curcuma longa*—in combination with the biomass‐derived solvent 2‐methyltetrahydrofuran establishes a fully organic, bio‐based catalytic system with a favorable environmental profile. Mechanistic investigations, including light on/off experiments, oxygen‐dependence studies, scavenger tests, Stern–Volmer and Hammett analysis, consistently support a sensitizer‐controlled, light‐gated aerobic pathway involving PET and oxygen‐mediated catalyst regeneration. The broad substrate scope, operational simplicity, and high selectivity further demonstrate the practicality of the protocol. To the best of our knowledge, this study represents the first example of curcumin employed as a photosensitizer for imine synthesis in organic chemistry. While the current homogeneous protocol does not involve the physical recovery of the pigment, ongoing studies are exploring the immobilization of curcumin on solid supports to facilitate the recovery of the photosensitizer toward future scale‐up.

## Funding

This study was partly supported by the project “Strengthening BIOdiversity Preservation through Sustainable Exploitation of the Bioresources CHAIN in the Programme Area‐ BIOCHAIN,” funded by the Interreg VI‐A Greece‐Italy 2021−2027 Programme.

## Conflicts of Interest

The authors declare no conflicts of interest.

## Supporting information

Supplementary Material

## Data Availability

The data that support the findings of this study are available on request from the corresponding author. The data are not publicly available due to privacy or ethical restrictions.
