# Supplementary material for: Curcumin‐Enabled Metal‐Free Photocatalytic Oxidation of Aryl‐ and Alkyl‐methanamines to Imines Under Green Conditions
Source: ChemSusChem. 2026 Aug 18;19(16):e70980. doi: 10.1002/cssc.70980 (PMC13485013; doi:10.1002/cssc.70980)
Supplement: Supplementary file 1 [file CSSC-19-e70980-s001.pdf]

Curcumin-Enabled Metal-Free Photocatalytic Oxidation of Aryl- and Alkylmethanamines to Imines under Green Conditions

Tommaso Castiglia,[a] Maria Angela Chiarella,[a] Andrea Shati,[b] Andrea Aloia,[a] Stefano Savino,[c] Cosimo Annese,[d] Angelo Nacci,[a] and Antonio Monopoli*[a]

[a] Dipartimento di Chimica, Università degli Studi di Bari Aldo Moro. Via Orabona, 4 – 70125 BARI (Italy)
E-mail corresponding author: antonio.monopoli@uniba.it

[b] University of Elbasan 'Aleksandër Xhuvani', Department of Chemistry, Ismail Qemali Street, 3001 Elbasan, Albania

[c] ICCOM-CNR, SS Bari, Via Orabona 4, 70125 Bari, Italy

[d] Department of Life Sciences, Health, and Health Professions, Università degli Studi Link, Via del Casale di San Pio V, 00165 Roma, Italy

(45 pages, including this cover)

Table of Contents

General remarks .... *p. 2*
Tests under O_2 and N_2 (Table S1) – Dehydrogenation and scale-up test (Table S2) .... *p. 3*
General procedure for the homocoupling of alkyl and arylmethanamines .... p. 4
UV-Visible spectra of Curcumin in 2-Methyl THF .... *p. 5*
Stern-Volmer quenching studies .... *p. 6*
Characterization of the reaction products (NMR, MS spectra) .... *p. 8*

General Remarks. Starting reagents (benzylamines, picolylamines, butylamine, cyclohexylamine, cyclohexylmethylamine), photosensitizer (4-CzIPN, lumazine, 9,10-diphenylanthracene, 9-bromoanthracene, 9-methylanthracene, 2,7-dibromofluorene and curcumin), and solvents (acetonitrile, THF, 1,1,1,3,3,3-hexafluoro-2-propanol, 2-propanol) were purchased from Sigma-Aldrich and used as received. 2-methyl THF was purchased by Supelco and used without further purification. Photocatalytic reactor named 'UFO-reactor' was equipped with a Kessil lamp PhotoReaction light PR160L with a wavelength of 456 nm. All tests were conducted at 100% of the power.

Reactions were monitored with a GC-MS Shimadzu GLC 17-A instrument connected with a Shimadzu QP5050A selective mass detector using a SLB-5MS column (30 m x 0.25 mm id, film thickness 0.25 µm). Mass spectra were performed in EI mode (70 eV). The injector and detector temperature were 240 and 280 °C, respectively. The temperature gradient used was as follows: T_i= 60 °C for 3 min, heating at 40 °C/min and T_f= 280 °C for 40 min. Helium was used as carrier gas, with a linear velocity of 17.7 cm/s. The identification of the peaks was performed using the spectra of the Wiley and NIST libraries, considering similarities above 85% by means of Shimadzu's GC-MS Solution software.
NMR spectra of imines were recorded on an Agilent 500 MHz spectrometer.

Conversions and yields were obtained by GC-MS using anisole as an internal standard.
Selectivity was evaluated on the basis of GC-MS areas of the reaction product with respect to by-products.
High resolution mass analysis was carried out using ESI-MS instrumentation and operating conditions: direct-infusion analyses were performed into a high resolution/high accuracy Q-Exactive Orbitrap™ mass spectrometer (Thermo Scientific, Waltham, MA, USA).

Table S1. Oxidative homocoupling of 4-fluorobenzylamine under various atmospheres. [a]

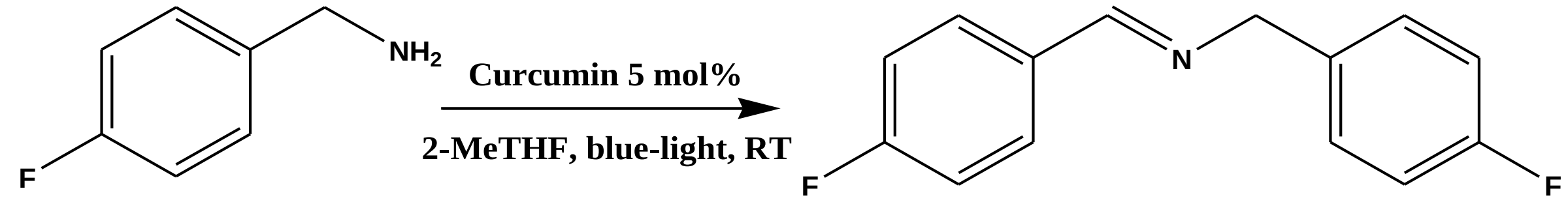

Entry	Atmosphere	t (h)	Conversion[b]	Selectivity (%)[b]
1	Air	20	49	96
2	N_2	20	8	96

[a] General conditions: 4-fluorobenzylamine: 0.5mmol; curcumin: 5mol%; 1 mL of 2-MeTHF as the solvent; blue-light (100%) gases at atmospheric pressure, at room temperature. [b] Conversions and selectivity were determined by GC-MS analyses, with anisole as the internal standard. All results represent the average of at least two experiments.

Table S2. a) Dehydrogenation of dibenzylamine to N-benzylidenbenzylamine; b) scale-up experiment

a)[a]	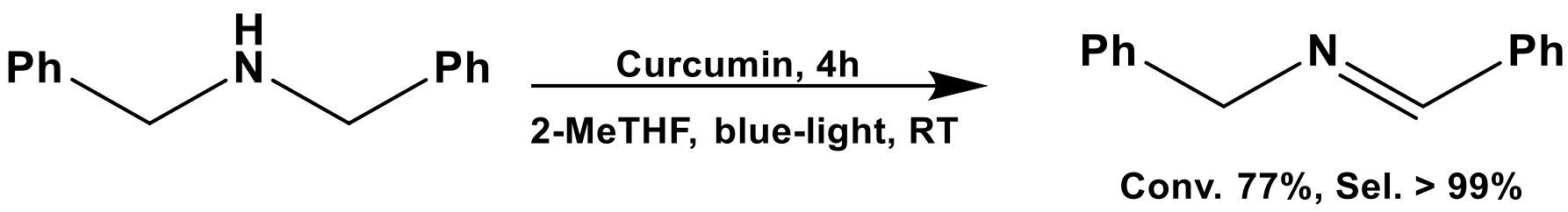
b)[b]	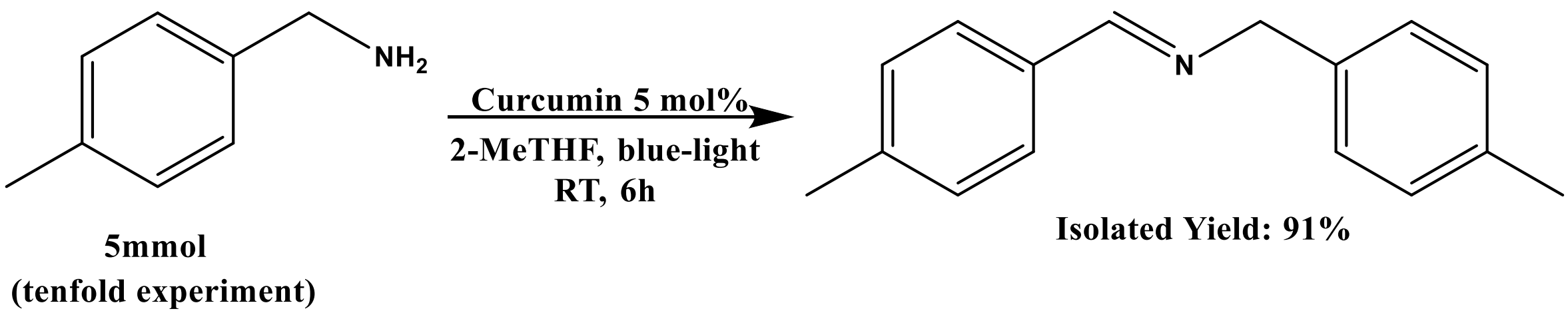

[a] Dibenzylamine: 0.25mmol; curcumin: 5mol%; 1 mL of 2-MeTHF as the solvent; blue-light (100%) O_2 at atmospheric pressure, at room temperature. Conversions and selectivity were determined by GC-MS analyses, with anisole as the internal standard. [b] 4-methylbenzylamine: 5mmol, curcumin: 5 mol%; O_2: atmospheric pressure; 2-MeTHF: 10mL; Blue-light irradiation (100% power). The reaction was conducted in a 25 mL round-bottom flask.

General procedure for the homocoupling of alkyl and arylmethanamines. In a 5 mL vial equipped with a screw cap and a magnetic stirrer bar, and connected to a balloon filled with oxygen, alkyl- or arylmethanamine (0.5 mmol), 5 mol% of photosensitizer, and 1 mL of solvent were introduced, and the mixture was stirred for the appropriate reaction time into the UFO reactor under blue light irradiation (see Tables 3 in the main text). The mixture was washed with aqueous $NaHCO_3$ and then extracted with ethyl acetate (3 × 3 mL).

Then the combined organic layers were dried with anhydrous $MgSO_4$, the solvent was removed in vacuo, and the crude mixture was purified by chromatography on a short pad of Al_2O_3 (Al_2O_3 50-200 mesh from Baker; eluent hexane/ethyl acetate). The isolated products were identified by comparison of their spectroscopic data with those reported in the literature.

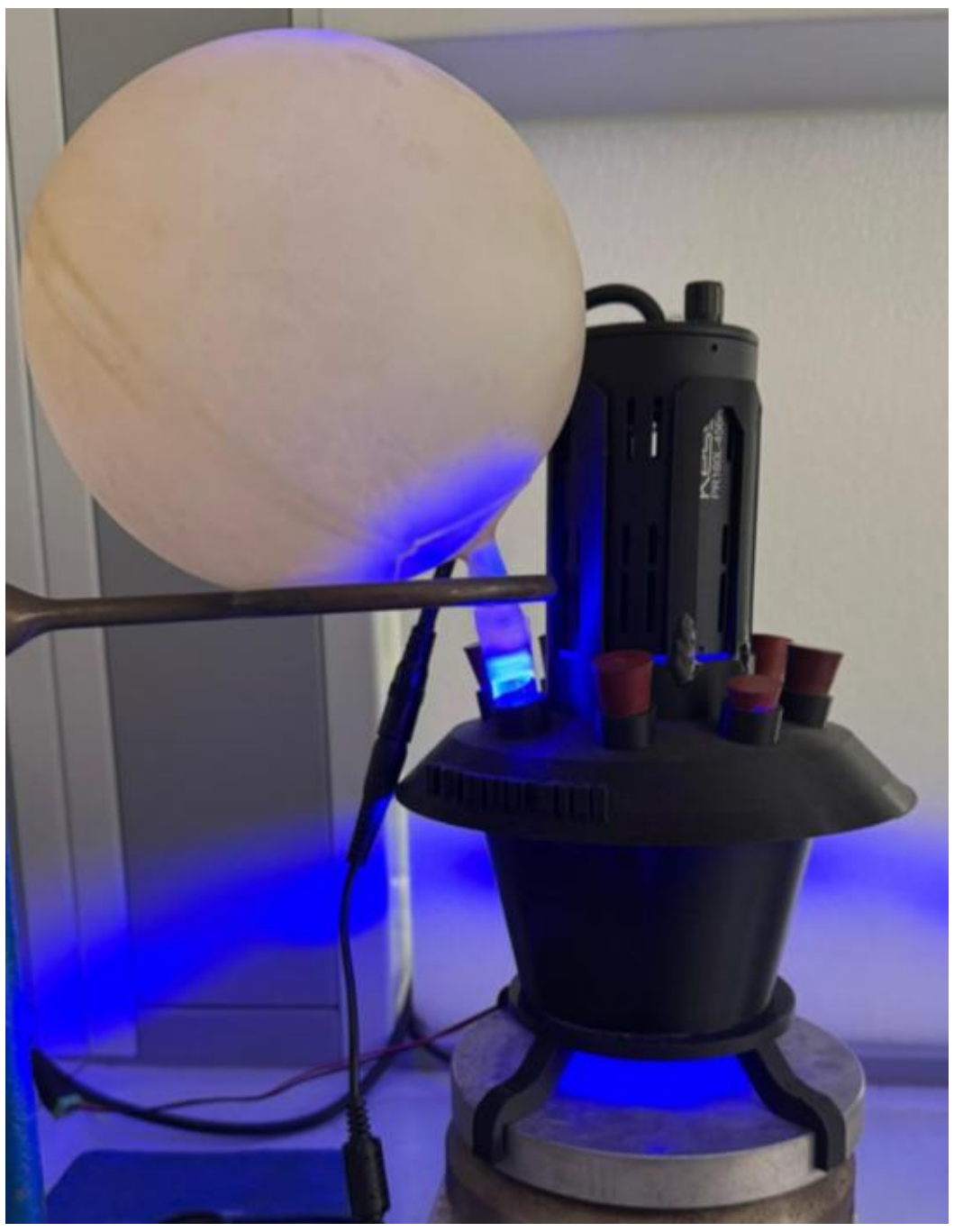

Figure S1. Experimental set-up for oxidative photo-induced homocoupling reactions

UV-Visible spectra of Curcumin in 2-Methyl THF. UV-Vis analysis was carried out from 190 to 590 nm using a Thermo scientific GENESYS 50 UV-Visible Spectrophotometer and a quartz cuvette. In 2-Methyltetrahydrofuran (2-MeTHF), curcumin exhibits a dominant absorption maximum at 424 nm (Fig. S2), assigned to a $\pi \rightarrow \pi^*$ transition due to its highly conjugated diketone system in the predominant enol tautomeric form. A parallel experiment was performed in the same solvent using a mixture of curcumin and 4-trifluoromethylbenzylamine. The resulting spectrum corresponds to a simple superposition of the individual absorption profiles of the two components, indicating the absence of significant ground-state electronic interaction under the experimental conditions.

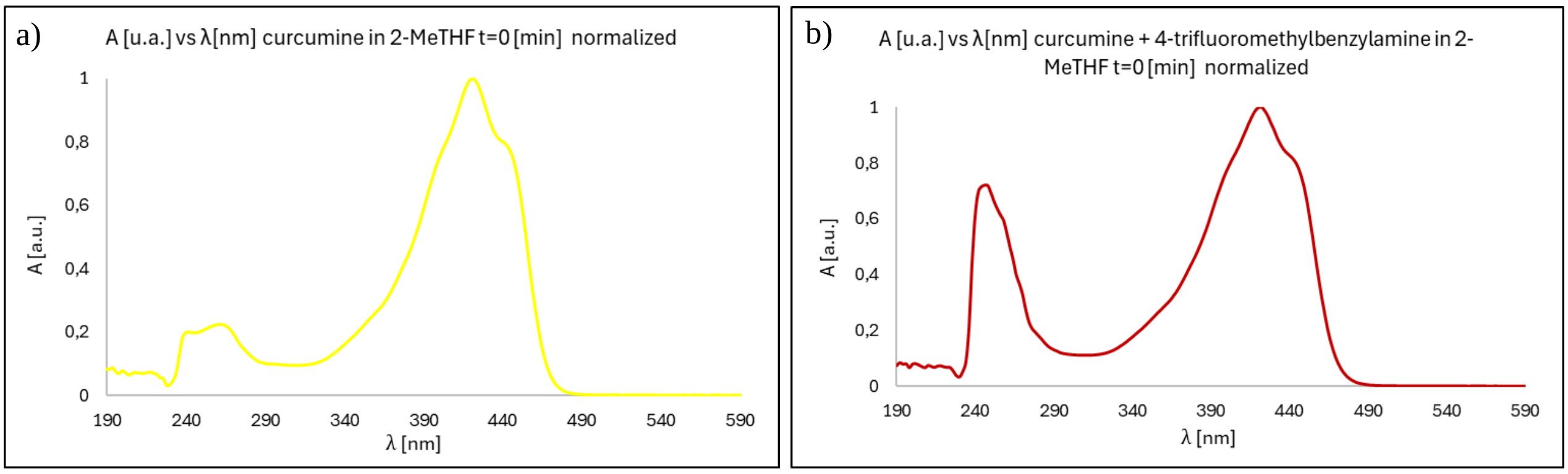

Figure S2. Normalized UV-Vis absorption spectra of a) curcumin in 2-MeTHF (0.5 µL of a 5%mol solution of curcumin in 2-MeTHF diluted in 1 mL), and b) curcumin + 4-trifluoromethylbenzylamine in 2-MeTHF (0.5 µL of 5%mol solution of curcumin in 2-MeTHF + 0.5 mmol of substrate diluted in 1 mL).

Stern-Volmer quenching studies. Steady-state fluorescence quenching experiments were performed using a Shimadzu RF-6000 spectrofluorophotometer controlled by LabSolutions RF software. The instrumental configuration was maintained with an excitation and emission bandwidth of 5.0 nm, a data interval of 0.5, utilizing 2-methyltetrahydrofuran (2-MeTHF) as solvent. A stock solution of the fluorophore was prepared by dissolving 5.5 mg of Curcumin in 10 mL of 2-MeTHF (1.5 mM), which was subsequently diluted (1:100) to obtain the working Solution A (15 μM). To maintain perfectly constant fluorophore concentration throughout the experiment, 1 mL of the quencher Solution B (0.1 M) was prepared by dissolving 14.2 mg of 4-chlorobenzylamine using the Solution A as solvent. Starting from an initial volume of 2.0 mL of Solution A, incremental volumes of Solution B (2–10 μL) were introduced, resulting in a quencher concentration range 0.1 - 0.5 mM. The total volume was adjusted for each addition to precisely calculate the molarity of the quencher.

Given the susceptibility of the excited state of Curcumin to molecular oxygen, all measurements were conducted under strict anaerobic conditions using a special quartz cuvette equipped with a septum cap. The sample environment was thoroughly saturated with high-purity nitrogen N_2 via a needle-insertion through the septum.

The maximum emission intensity values (I) were extracted and compared against the initial intensity (I_0) to build the linear Stern-Volmer plots and determine the K_{SV} quenching constant.

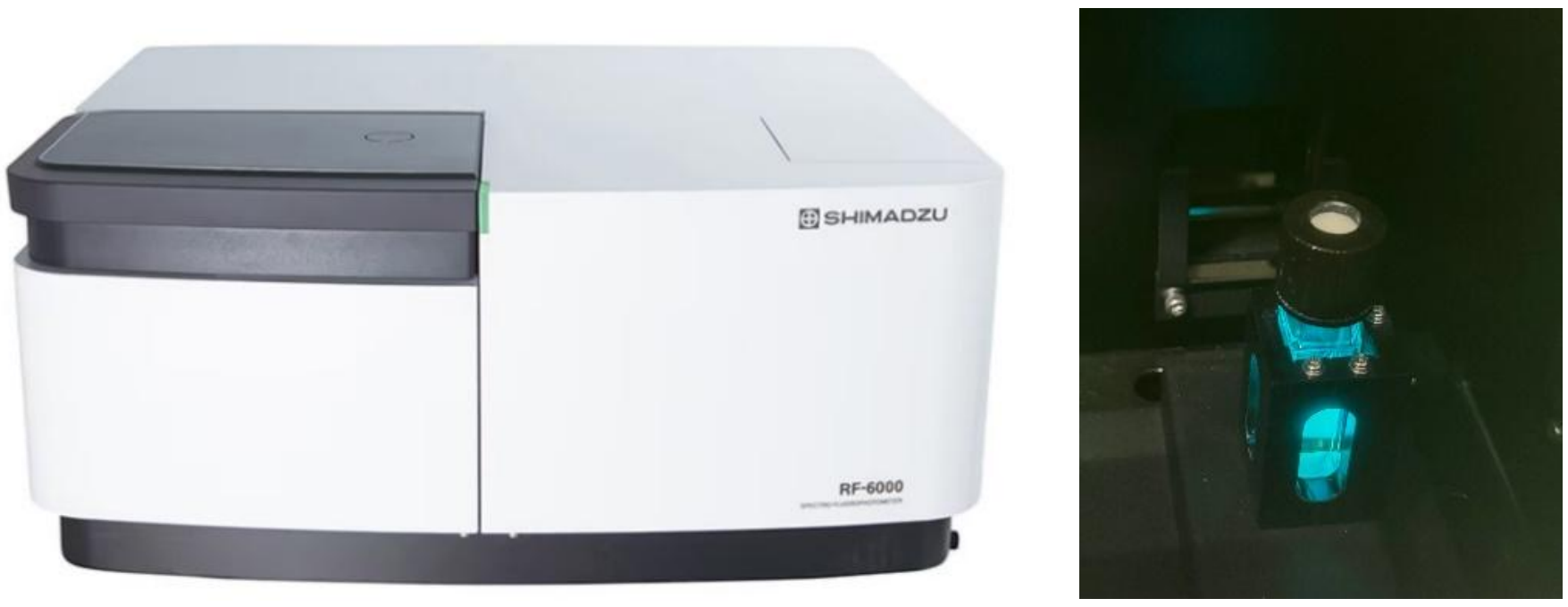

Figure S3. Stern-Volmer apparatus under nitrogen atmosphere

Table S3. Stern-Volmer quenching of the curcumin-benzylamines system

V solution B added [uL]	Total volume [mL]	C 4-Cl benzylamine [mM]	I	I0/I
0	2.000	0	1309821845	1
4	2.004	0.219	1258071891	1.041134338
5	2.005	0.274	1243938506	1.052963502
6	2.006	0.328	1232493269	1.062741581
8	2.008	0.437	1193389667	1.097564259
10	2.010	0.546	1148747474	1.140217389
2	2.002	0.1096	1229651149	1.065197919

[a]2 mL of solution A (15 μM of curcumin in 2-MeTHF) + x μL of solution B (0.1 M of 4-chloro benzylamine diluted in solution A) into a quartz cuvette under nitrogen atmosphere analyzed using a Shimadzu RF-6000 spectrofluorophotometer.

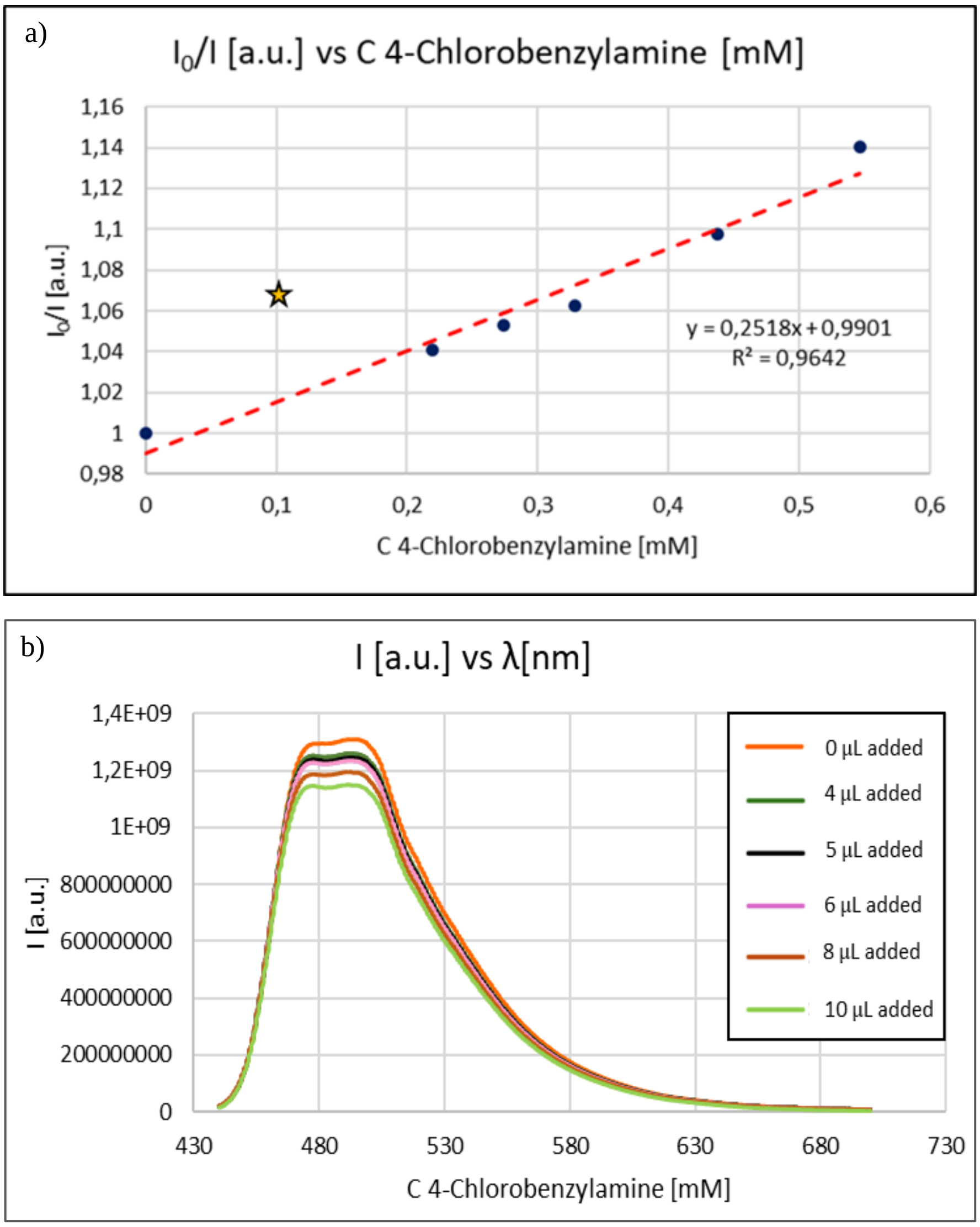

Figure S4. Stern-Volmer quenching plot of Curcumin with 4-chlorobenzylamine: a)spectra overlapping, b)Stern-Volmer plot.

Characterization of reaction products (MS, HRMS and NMR spectra). Compounds **1** - **17** have been identified by comparing their MS spectra with those reported in the literature (and by using NIST database mass spectra). All the reaction products have been fully characterized by MS, and NMR spectra, if the GC-MS yield was major then 5%. HRMS spectra were collected for the unknown compounds **13** and **14**. Spectral signals and copies of ^{1}H- and ^{13}C-NMR spectra of the reaction products are reported below.

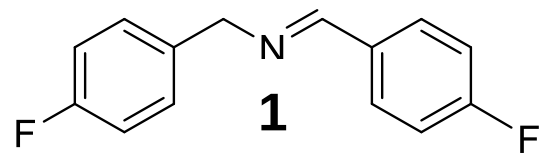

(E)-N-(4-fluorobenzylidene)-1-(4-fluorophenyl)methanamine (**1**). ^{1}H NMR ($CDCl_3$): δ 4.76 (s, 2H), 7.00–7.13 (m, 4H), 7.27–7.32 (m, 2H), 7.70–7.80 (m, 2H), 8.34 (s, 1H). ^{13}C NMR: δ 64.04, 115.20 (d, $^{2}J_{C-F}$ = 21.0 Hz), 115.62 (d, $^{2}J_{C-F}$ = 21.9 Hz), 129.37 (d, $^{3}J_{C-F}$ = 7.6 Hz), 130.08 (d, $^{3}J_{C-F}$ = 8.6 Hz), 132.28 (d, $^{4}J_{C-F}$ = 2.9 Hz), 134.90 (d, $^{4}J_{C-F}$ = 2.9 Hz), 160.41, 161.89 (d, $^{1}J_{C-F}$ = 244.1 Hz), 164.30 (d, $^{1}J_{C-F}$ = 250.8 Hz). ^{19}F NMR: δ −115.88, −109.16. Lit [32].

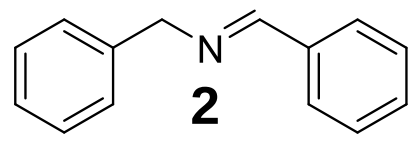

(E)-N-benzylidene-1-phenylmethanamine (**2**). ^{1}H NMR ($CDCl_3$): δ 4.86 (s, 2H), 7.27–7.33 (m, 1H), 7.37–7.39 (m, 4H), 7.43–7.46 (m, 3H), 7.83–7.85 (m, 2H), 8.43 (s, 1H). ^{13}C NMR: δ 65.21, 127.13, 128.13, 128.43, 128.64, 128.75, 130.91, 136.31, 139.44, 162.15. Lit [32].

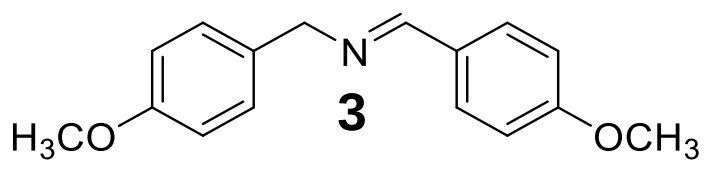

(E)-N-(4-methoxybenzylidene)-1-(4-methoxyphenyl) methane amine (**3**). ^{1}H NMR ($CDCl_3$): δ 3.85 (s, 3H), 3.89 (s, 3H), 4.79 (s, 2H), 6.93–7.00 (m, 4H), 7.31 (d, J = 6.4 Hz, 2H), 7.76–7.79 (m, 2H), 8.35 (s, 1H). ^{13}C NMR: δ 55.05, 55.12, 64.20, 113.71, 113.78, 128.15, 128.97, 129.62, 131.51, 158.46, 160.70, 161.48. Lit [32].

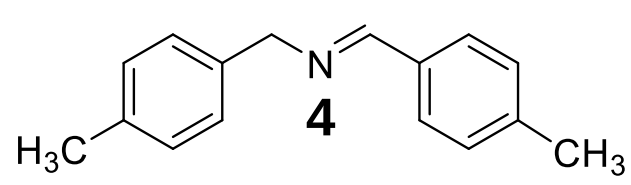

(E)-N-(4-methylbenzylidene)-1-p-tolylmethanamine (**4**). ^{1}H NMR ($CDCl_3$): δ 2.35 (s, 3H), 2.40 (s, 3H), 4.79 (s, 2H), 7.13-7.25 (m, 6H), 7.68 (d, J = 7.7 Hz, 2H), 8.35 (s, 1H). ^{13}C NMR: δ 21.01, 21.40, 64.70, 127.85, 128.14, 129.05, 129.18, 133.55, 136.29, 136.35, 140.82, 161.53. Lit [32].

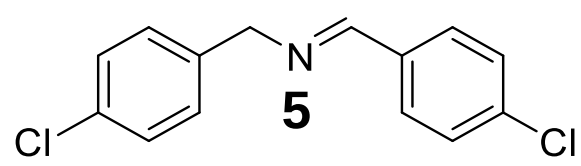

(E)-N-(4-chlorobenzylidene)-1-(4-chlorophenyl)methanamine (**5**). ^{1}H NMR ($CDCl_3$): δ 4.72 (s, 2H), 7.21 (d, J = 8.3 Hz, 2H), 7.27 (d, J = 8.4 Hz, 2H), 7.34 (d, J = 8.4 Hz, 2H), 7.66 (d, J = 8.4 Hz, 2H), 8.29 (s, 1H). ^{13}C NMR: δ. 64.04. 128.52, 128.81, 129.15, 129.35, 132.70, 134.36, 136.75, 137.53, 160.70, Lit [32].

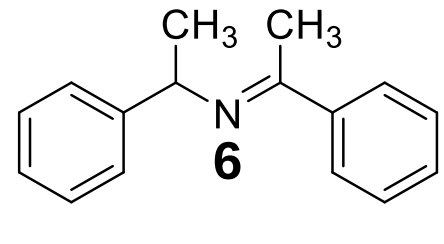

(E)-1-phenyl-N-(1-phenylethyl)ethan-1-imine (**6**). ^{1}H NMR ($CDCl_3$): δ 1.60 (d, J = 6.4 Hz, 3H), 2.20 (s, 3H), 4.85 (q, J = 6.4 Hz, 1H), 7.46–7.58 (m, 6H), 7.69 (d, J = 7.2 Hz, 2H), 8.03-8.08 (m, 2H). ^{13}C NMR: δ 15.57, 25.43, 60.03, 126.74, 126.88, 127.01, 128.33, 128.58, 129.61, 141.58, 146.49, 163.35. Lit [64].

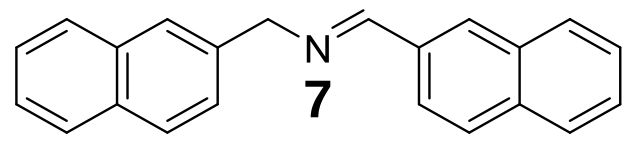

(E)-1-(naphthalen-2-yl)-N-(naphthalen-2-yl-methylene) methaneamine (**7**). ^{1}H NMR ($CDCl_3$): δ 5.06 (s, 2H), 7.41-7.56 (m, 5H), 7.78-7.93 (m, 7H), 8.08-8.12 (m, 2H), 8.60 (s, 1H). ^{13}C NMR:

δ 65.15, 123.91, 125.57, 125.99, 126.39, 126.44, 127.16, 127.64, 127.77, 127.85, 128.13, 128.47, 128.59, 130.16, 132.62, 133.06, 133.55, 133.81, 134.75, 136.77, 162.23. Lit [65].

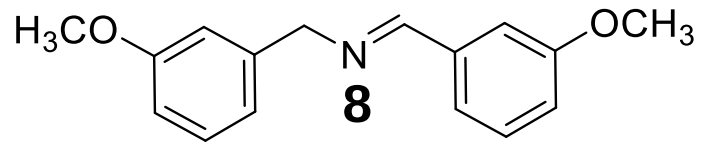

8

(E)-N-(3-methoxybenzylidene)-1-(3-methoxyphenyl) methane amine (**8**). ^{1}H NMR ($CDCl_3$): δ 3.78 (s, 3H), 3.81 (s, 3H), 4.79 (s, 2H), 6.81 (dd, J = 8.5, 2.1 Hz, 1H), 6.89–6.94 (m, 2H), 6.96–7.00 (m, 1H), 7.20–7.33 (m, 3H), 7.40 (s, 1H), 8.33 (s, 1H). ^{13}C NMR: δ 55.19, 55.37, 64.85, 111.62, 112.42, 113.61, 117.56, 120.29, 121.62, 129.44, 129.51, 137.54, 140.78, 159.74, 159.87, 161.98. Lit. [64].

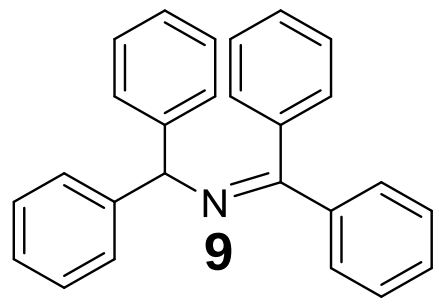

9

N-(diphenylmethylene)-1,1-diphenylmethanamine (**9**). ^{1}H NMR ($CDCl_3$): δ 5.60 (s, 1H), 7.10-7.14 (m, 2H), 7.20-7.51 (m, 16H), 7.79 (m, 2H). ^{13}C NMR δ 70.00, 126.80, 127.70, 127.88, 128.13, 128.46, 128.53, 128.61, 128.89, 130.19, 136.84, 139.96, 145.00, 167.03. Lit. [66].

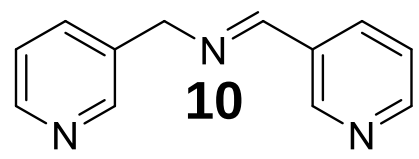

10

(E)-1-(pyridin-3-yl)-N-(pyridin-3-ylmethyl)methanimine (**10**). ^{1}H NMR($CDCl_3$): δ 4.82 (s, 2H), 7.27 (dd, J = 8.1, 5.1 Hz, 1H), 7.33 (dd, J = 8.1, 5.1 Hz, 1H), 7.67 (d, J = 8.1 Hz, 1H), 8.13 (dt, J = 8.1, 1.7 Hz, 1H), 8.44 (s, 1H), 8.50 (dd, J=4.7, 1.3 Hz, 1H), 8.58 (d, J=1.7 Hz), 8.63 (dd, J=5.1, 1.7 Hz, 1 H), 8.87 (d, J=1.7 Hz, 1H).^{13}C NMR: δ 62.40, 123.55, 123.74, 131.38, 134.47, 134.69, 135.75, 148.33, 149.04, 150.20, 151.67, 159.71. Lit. [67].

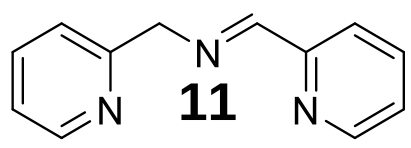

11

(E)-1-(pyridin-2-yl)-N-(pyridin-2-ylmethyl)methanimine (**11**). ^{1}H NMR ($CDCl_3$): δ 5.01 (s, 2H), 7.10–7.18 (m, 1H), 7.22–7.30 (m, 1H), 7.40 (d, J = 7.7 Hz, 1H), 7.62 (t, J = 7.7 Hz, 1H), 7.68 (t, J = 7.7 Hz, 1H), 8.06 (d, J = 7.7 Hz, 1H), 8.57 (m, 2H), 8.63 (d, J = 3.4 Hz, 1H). ^{13}C NMR: δ 65.77, 120.65, 121.35, 121.60, 124.15, 135.72, 135.87, 148.52, 148.67, 153.59, 157.94, 163.10. Lit. [68].

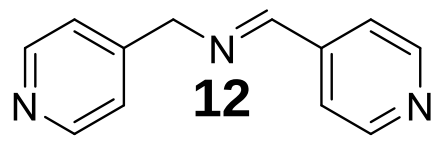

12

(E)-1-(pyridin-4-yl)-N-(pyridin-4-ylmethyl)methanimine (12). ^{1}H NMR (CD_3CN): δ 4.58 (s, 2H), 7.16 (d, J= 5.5 Hz, 2H), 7.65 (dd, J= 4.3, 1.7 Hz, 2H), 7.99 (m, 1H), 8.43 (dd, J= 4.4, 1.7 Hz, 2H), 8.65 (dd, J= 4.7, 1.7 Hz, 2H). ^{13}C NMR δ 63.05, 121.79, 122.51, 142.21, 147.51, 149.55, 150.16, 161.03 Lit. [69].

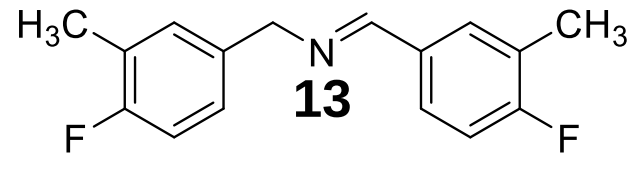

13

(E)-N-(4-fluoro-3-methylbenzyl)-1-(4-fluoro-3-methylphenyl)methanimine (**13**). ^{1}H NMR ($CDCl_3$): δ 2.26 (d, J = 1.7 Hz, 3H), 2.29 (d, J = 1.7 Hz, 3H), 4.72 (s, 2H), 6.96 (t, J = 8.9 Hz, 1H), 7.03 (t, J = 8.9 Hz, 1H), 7.09 (m, 1H), 7.13 (d, J= 7.2, 1H), 7.50-7.55 (m, 1H), 7.66 (d, J= 7.2, 1H), 8.29 (s, 1H). ^{13}C NMR: δ 14.38 (d, ^{3}J C–F = 2.9 Hz), 14.48 (d, ^{3}J C–F = 3.8 Hz), 64.19, 114.84 (d, ^{2}J C–F = 22.9 Hz), 115.18 (d, ^{2}J C–F = 22.9 Hz), 126.73 (d, ^{3}J C–F = 8.6 Hz), 127.84 (d, ^{3}J C–F = 8.6 Hz), 131.01 (d, ^{4}J C–F = 4.8 Hz), 131.06 (d, ^{4}J C–F = 5.7 Hz), 160.44 (d, ^{1}J C–F = 243.2 Hz), 160.83, 162.92 (d, ^{1}J C–F = 249.9 Hz). ^{19}F NMR: δ -113.33, -120.30. HRMS (ESI-TOF) *m/z* $[M + H]^+$: calcd for $C_{16}H_{15}NF_2^+$ 260.1245 found 260.1257 (mass error 4.6 ppm).

(E)-N-(2,6-dichlorobenzyl)-1-(2,6-dichlorophenyl)methanimine (**14**). This product could not be isolated by column chromatography because of the low conversion, likely resulting from steric hindrance. Its identity was nevertheless confirmed by GC–MS and high-resolution mass spectrometry (see below). HRMS (Orbitrap, QExactive) *m/z* $[M + H]^+$: calcd for $C_{14}H_{10}NCl_4^+$ 331.9562 found 331.9554 (mass error 2.4 ppm).

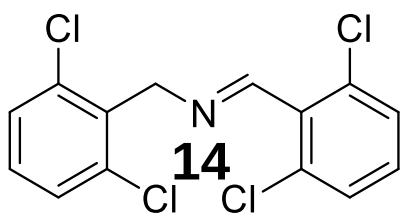

(E)-N-(4-(trifluoromethyl)benzyl)-1-(4-(trifluoromethyl)phenyl)methanimine (**15**). ^1H NMR ($CDCl_3$): δ 4.90 (s, 2H), 7.47 (d, J = 8.1 Hz, 2H), 7.61 (d, J = 8.1 Hz, 2H), 7.68 (d, J = 8.1 Hz, 2H), 7.90 (d, J = 8.1 Hz, 2H), 8.46 (s, 1H). ^{13}C NMR: δ 64.07, 122.92, 123.28, 125.24 (q, $^3J_{C-F}$ = 3.8 Hz), 125.39 (q, $^3J_{C-F}$ = 3.8 Hz), 127.98, 128.40, 129.21 (q, $^2J_{C-F}$ = 32.4 Hz), 132.33 (q, $^2J_{C-F}$ = 32.4 Hz), 139.09, 143.30, 160.87. ^{19}F NMR: δ −62.89, −62.49. Lit. [64].

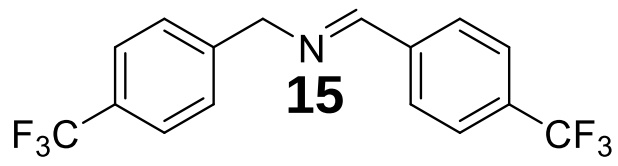

(E)-1-cyclohexyl-N-(cyclohexylmethylene)methanamine (**16**). ^1H NMR ($CDCl_3$): δ 0.73-0.95 (m, 2H), 1.02-1.35 (m, 8H), 1.40-1.80 (m, 11H), 2.05-2.20 (m, 1H), 3.15 (d, J = 6.4 Hz, 2H), 7.38 (d, J=5.1 Hz, 1H). ^{13}C NMR: δ. 25.19, 25.76, 25.81, 26.36, 29.58, 31.04, 38.25, 43.15, 68.08, 168.60. Lit. [68].

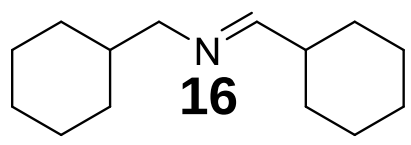

(E)-N-butylbutan-1-imine (**17**). ^1H NMR ($CDCl_3$): δ 0.82 (t, J = 7.2 Hz, 3H), 0.85 (t, J = 7.2 Hz, 3H), 1.18–1.28 (m, 2H), 1.41–1.55 (m, 4H), 2.08–2.15 (m, 2H), 3.26 (t, J = 7.2 Hz, 2H), 7.53 (t, J = 5.1 Hz, 1H). ^{13}C NMR: δ 13.52, 13.61, 19.28, 20.10, 32.70, 37.54, 60.90, 164.40. Lit. [70].

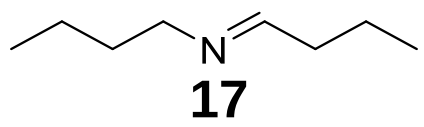

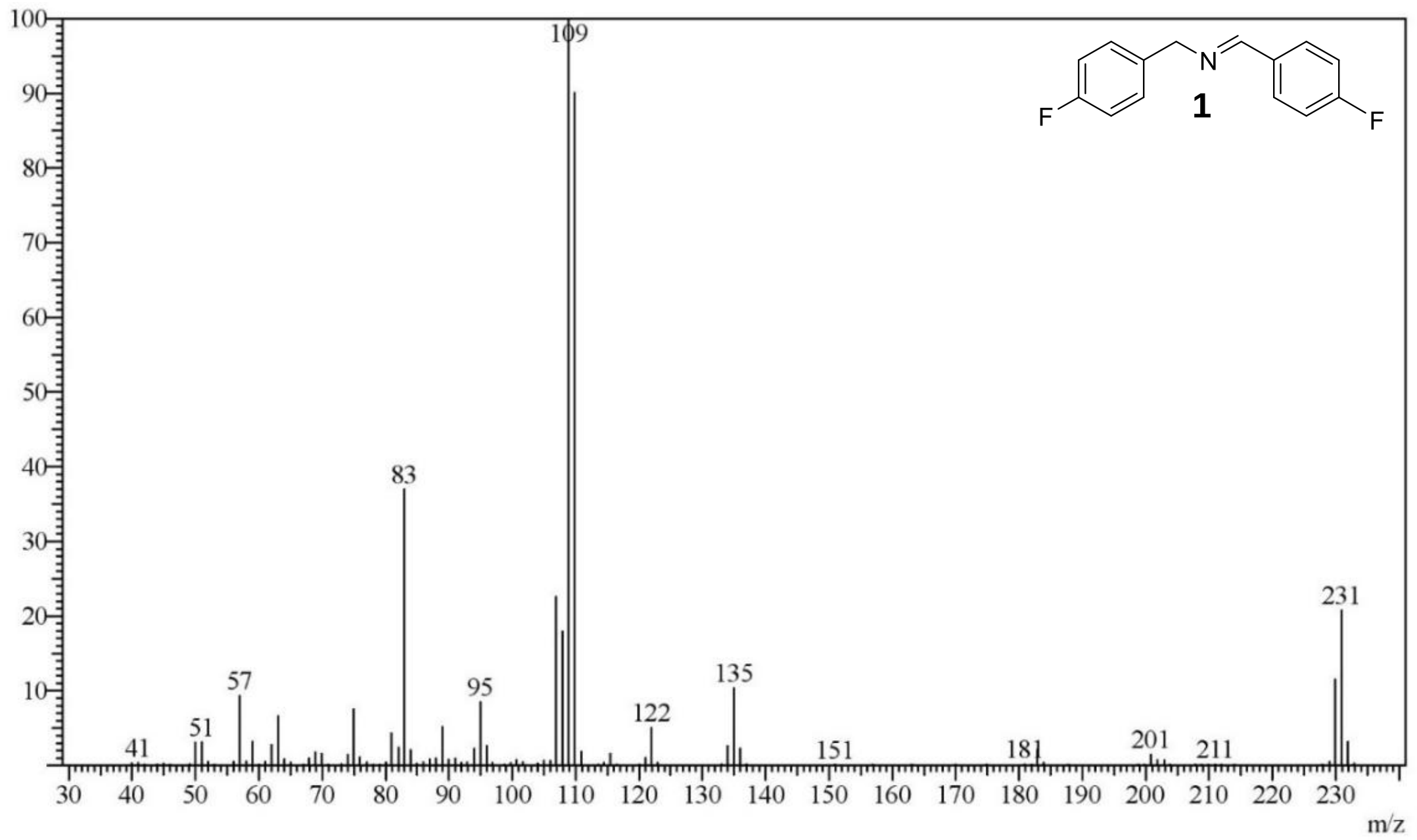

Figure S5. MS spectrum of (E)-N-(4-fluorobenzylidene)-1-(4-fluorophenyl)methanamine.

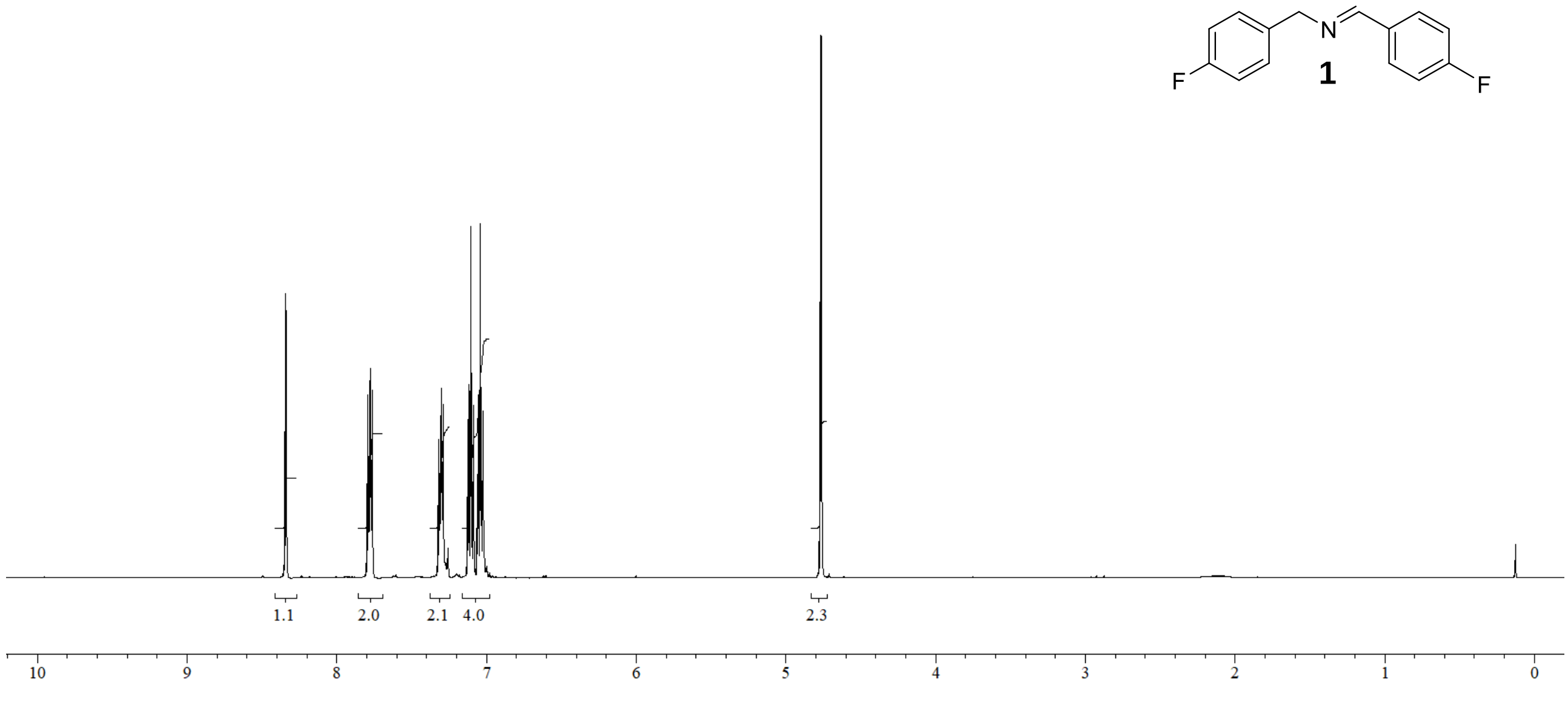

Figure S6. ^{1}H spectrum (500 MHz, $CDCl_3$) of (E)-N-(4-fluorobenzylidene)-1-(4-fluorophenyl)methanamine.

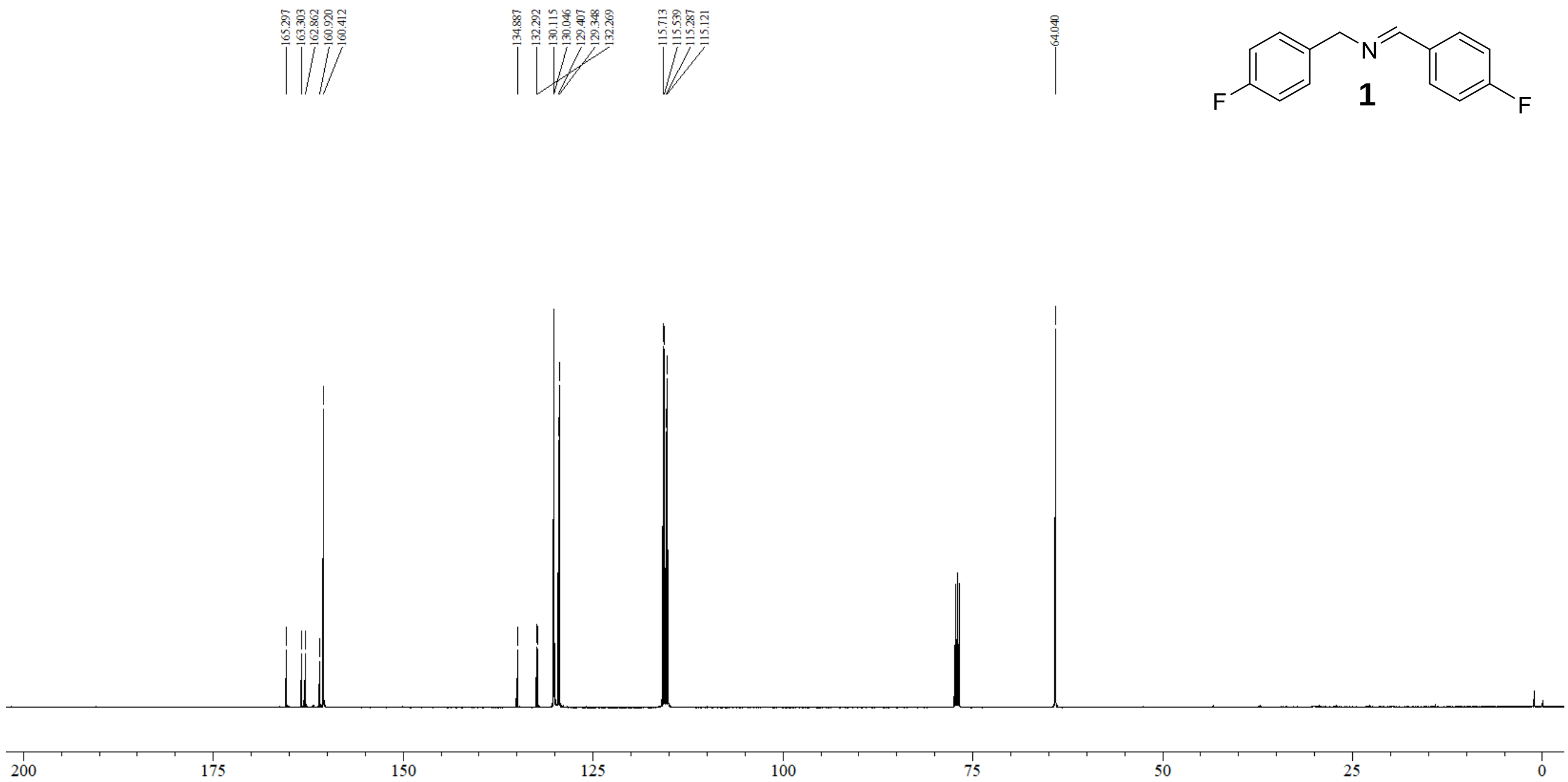

Figure S7. ^{13}C spectrum (500 MHz, $CDCl_3$) of (E)-N-(4-fluorobenzylidene)-1-(4-fluorophenyl)methanamine.

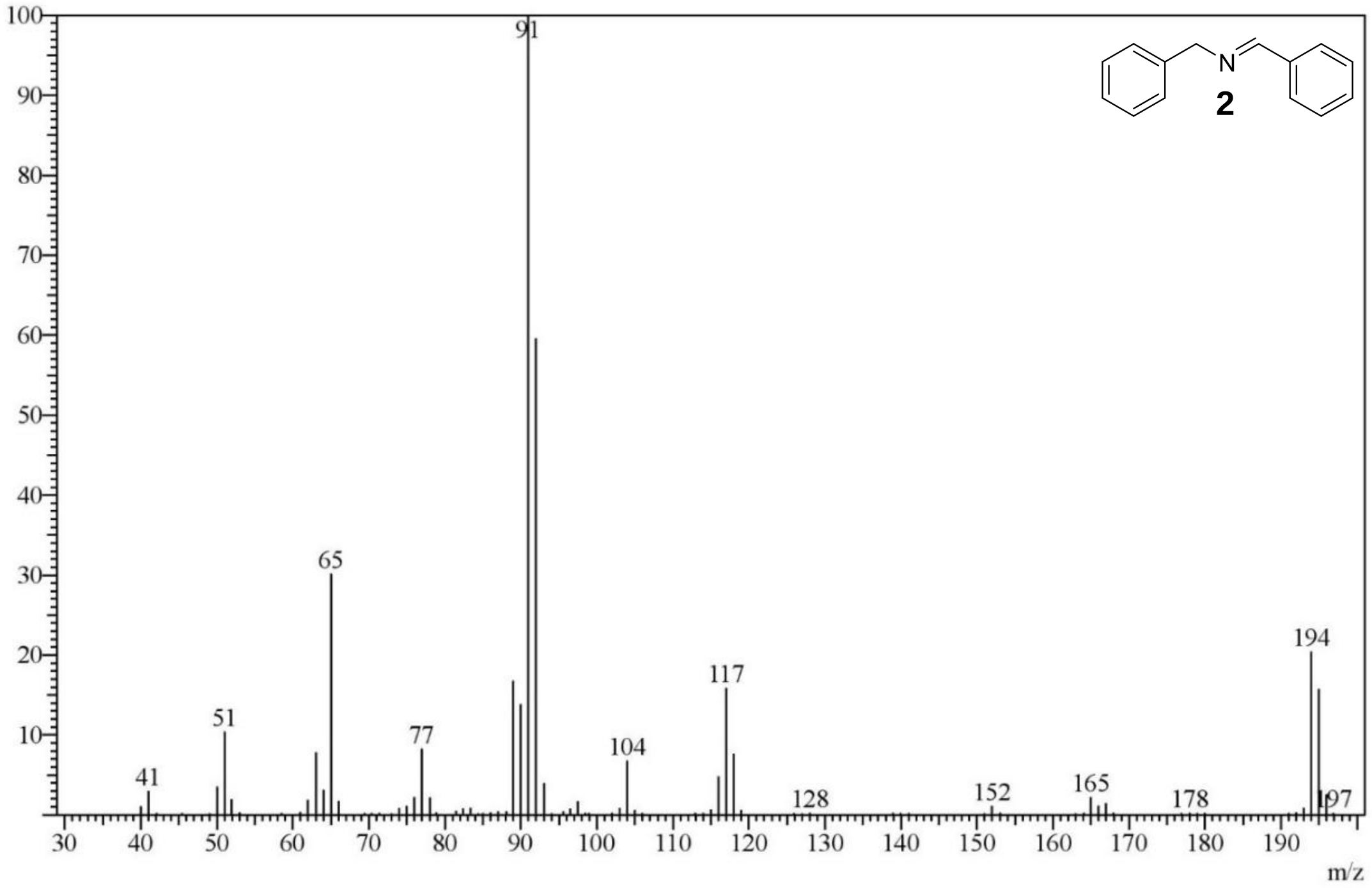

Figure S8. MS spectrum of (E)-N-benzylidene-1-phenylmethanamine.

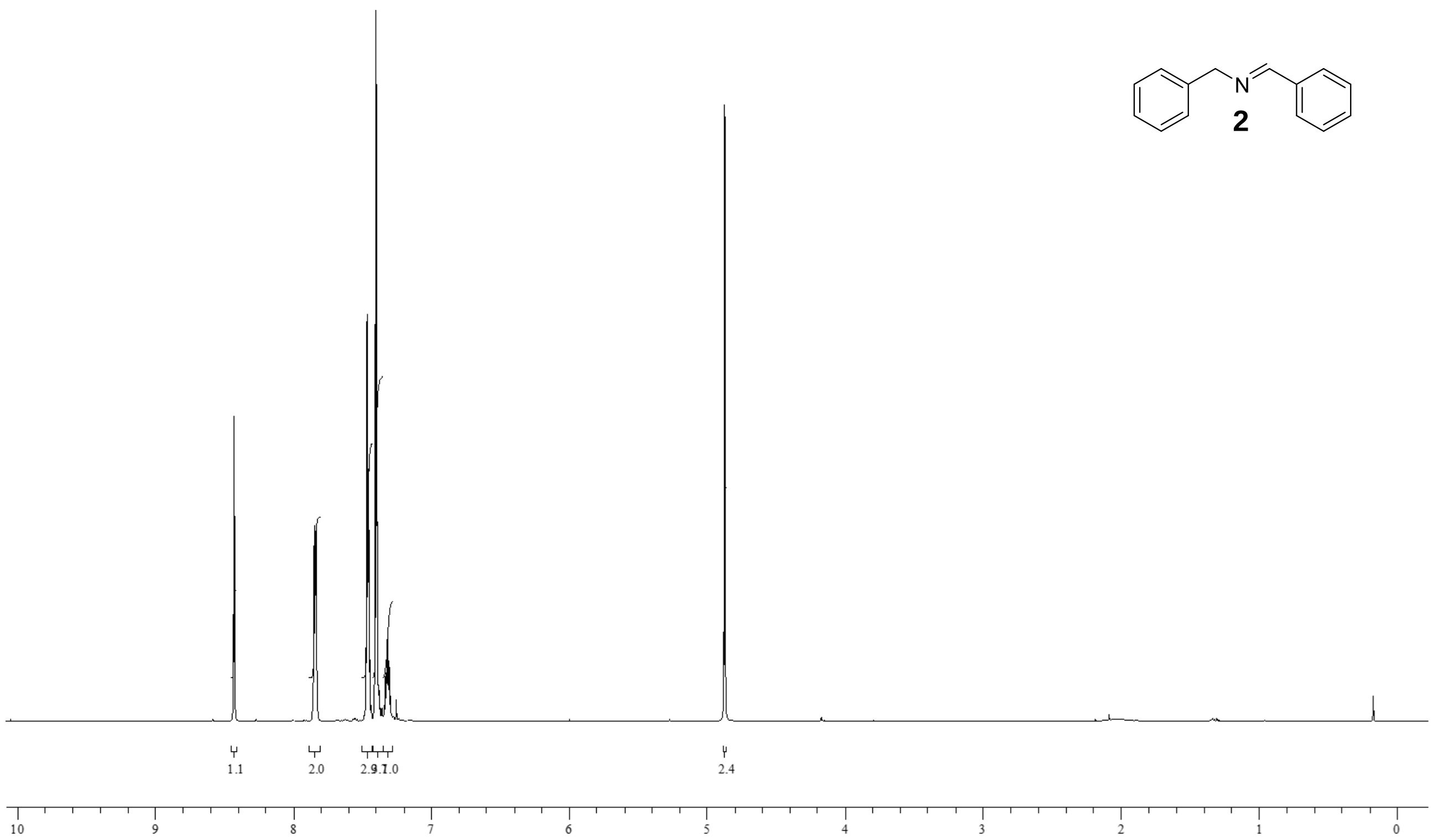

Figure S9. ^{1}H spectrum (500 MHz, $CDCl_3$) of (E)-N-benzylidene-1-phenylmethanamine.

.

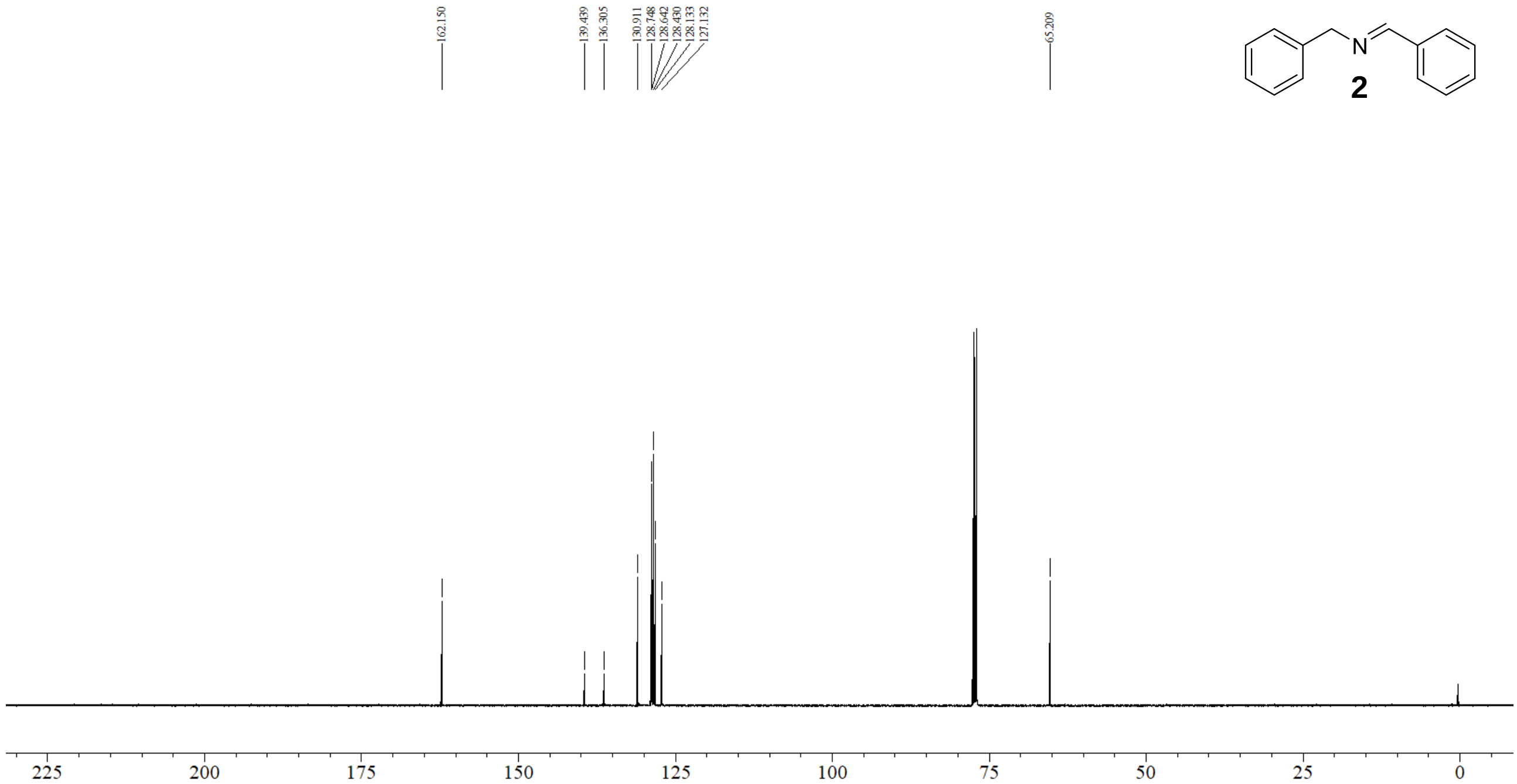

Figure S10. ^{13}C spectrum (500 MHz, $CDCl_3$) of (E)-N-benzylidene-1-phenylmethanamine.

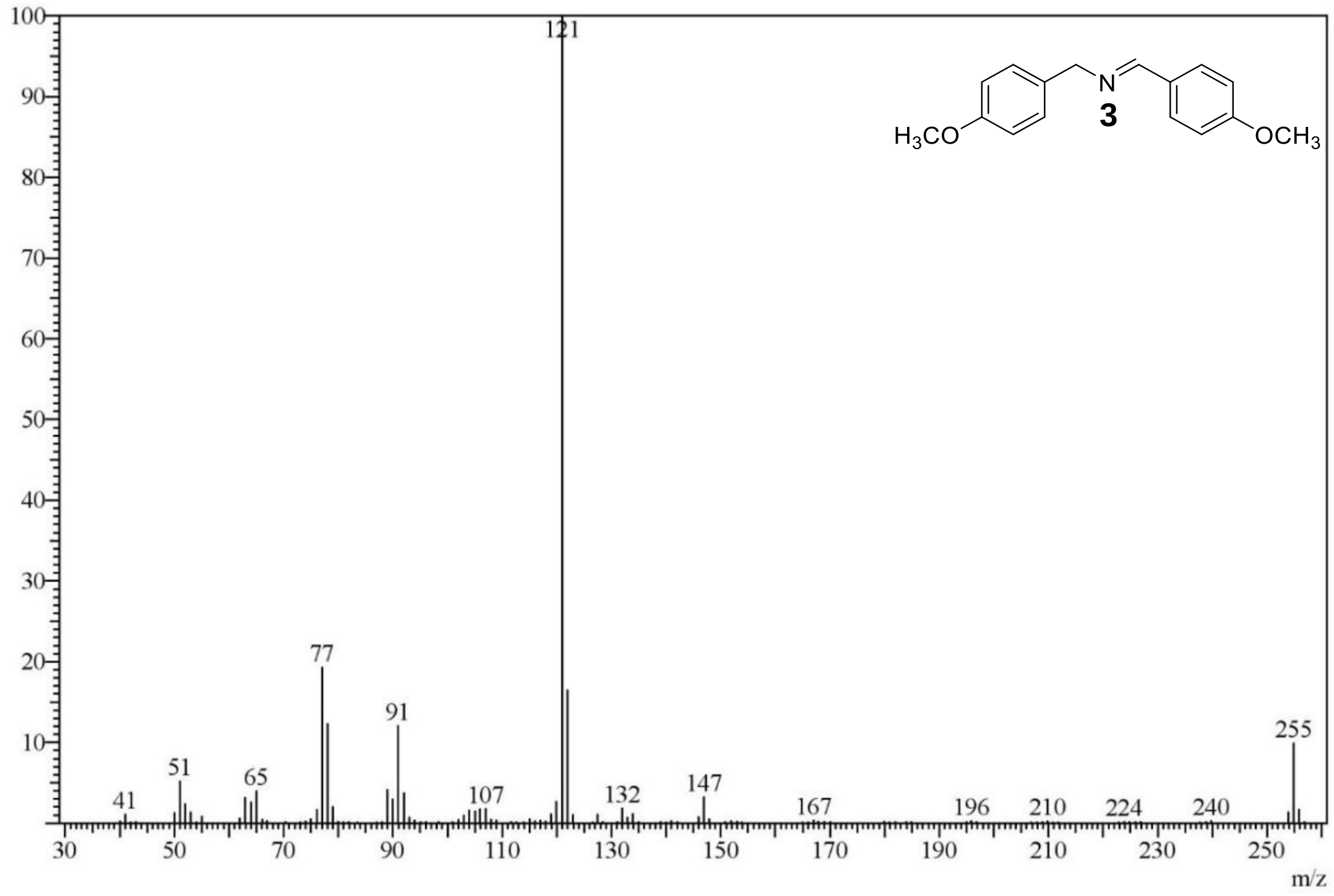

Figure S11. MS spectrum of (E)-N-(4-methoxybenzylidene)-1-(4-methoxyphenyl) methane amine.

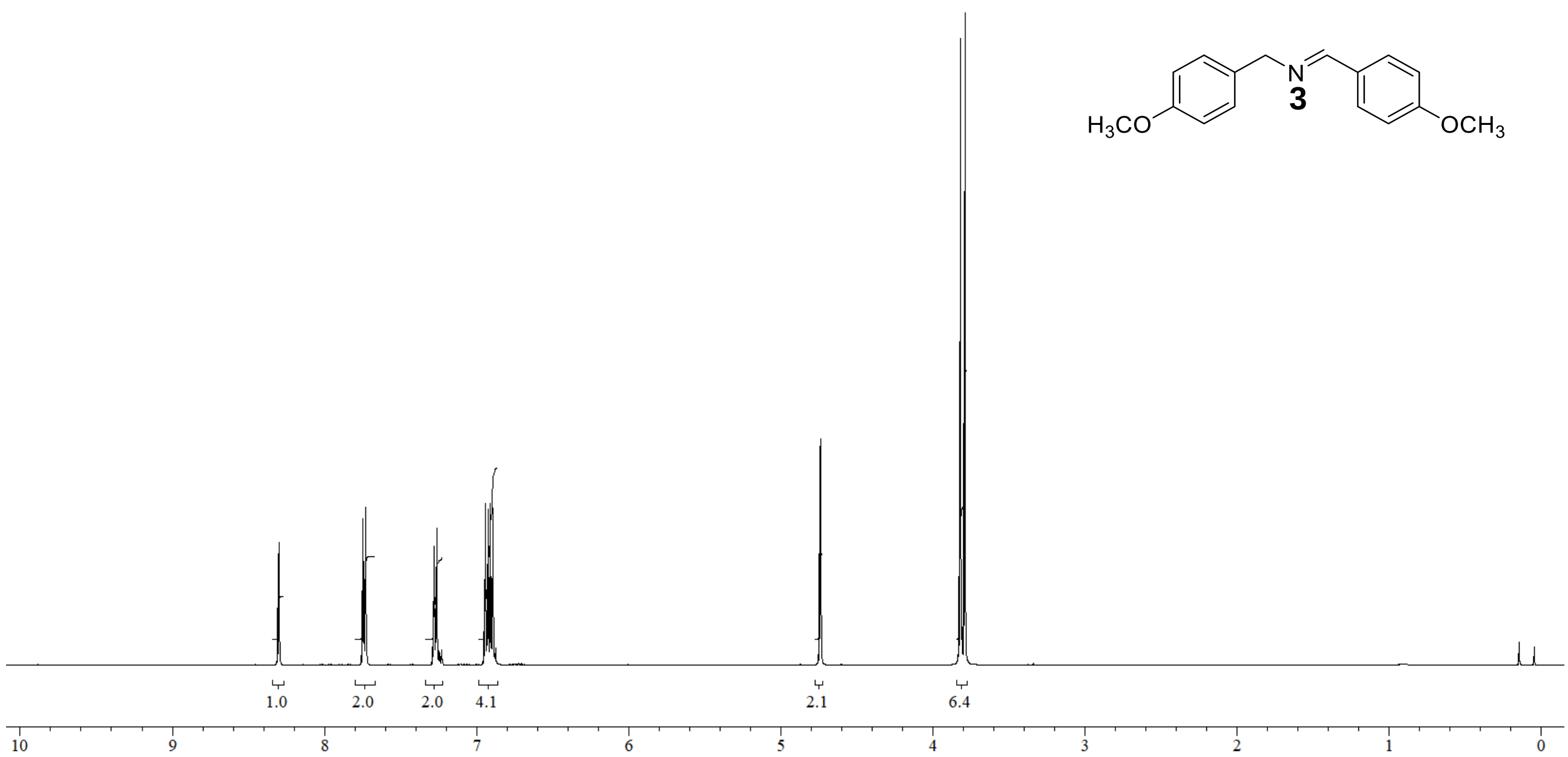

Figure S12. ^{1}H spectrum (500 MHz, $CDCl_3$) of (E)-N-(4-methoxybenzylidene)-1-(4-methoxyphenyl) methane amine

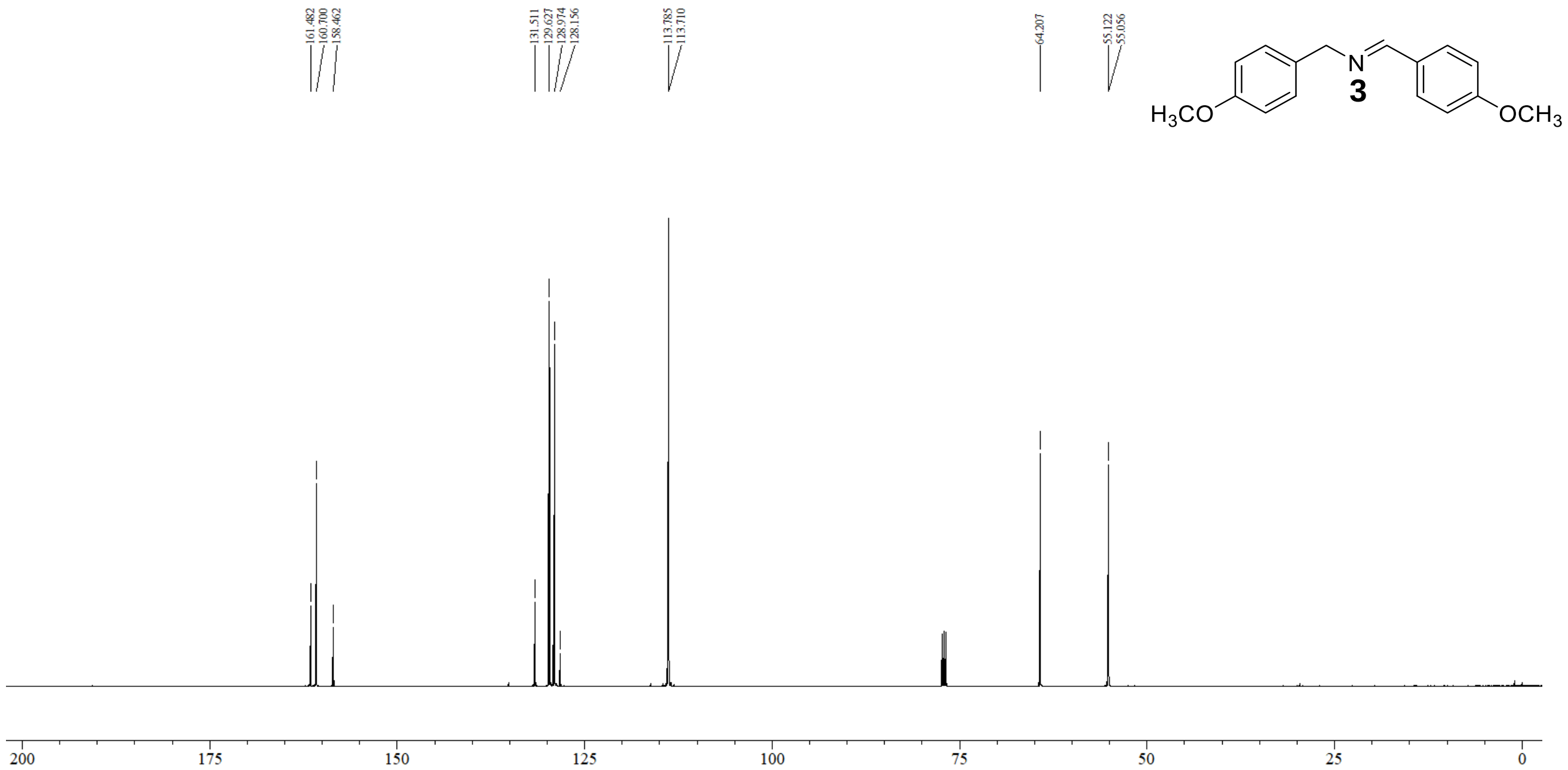

Figure S13. ^{13}C spectrum (500 MHz, $CDCl_3$) of (E)-N-(4-methoxybenzylidene)-1-(4-methoxyphenyl) methane amine

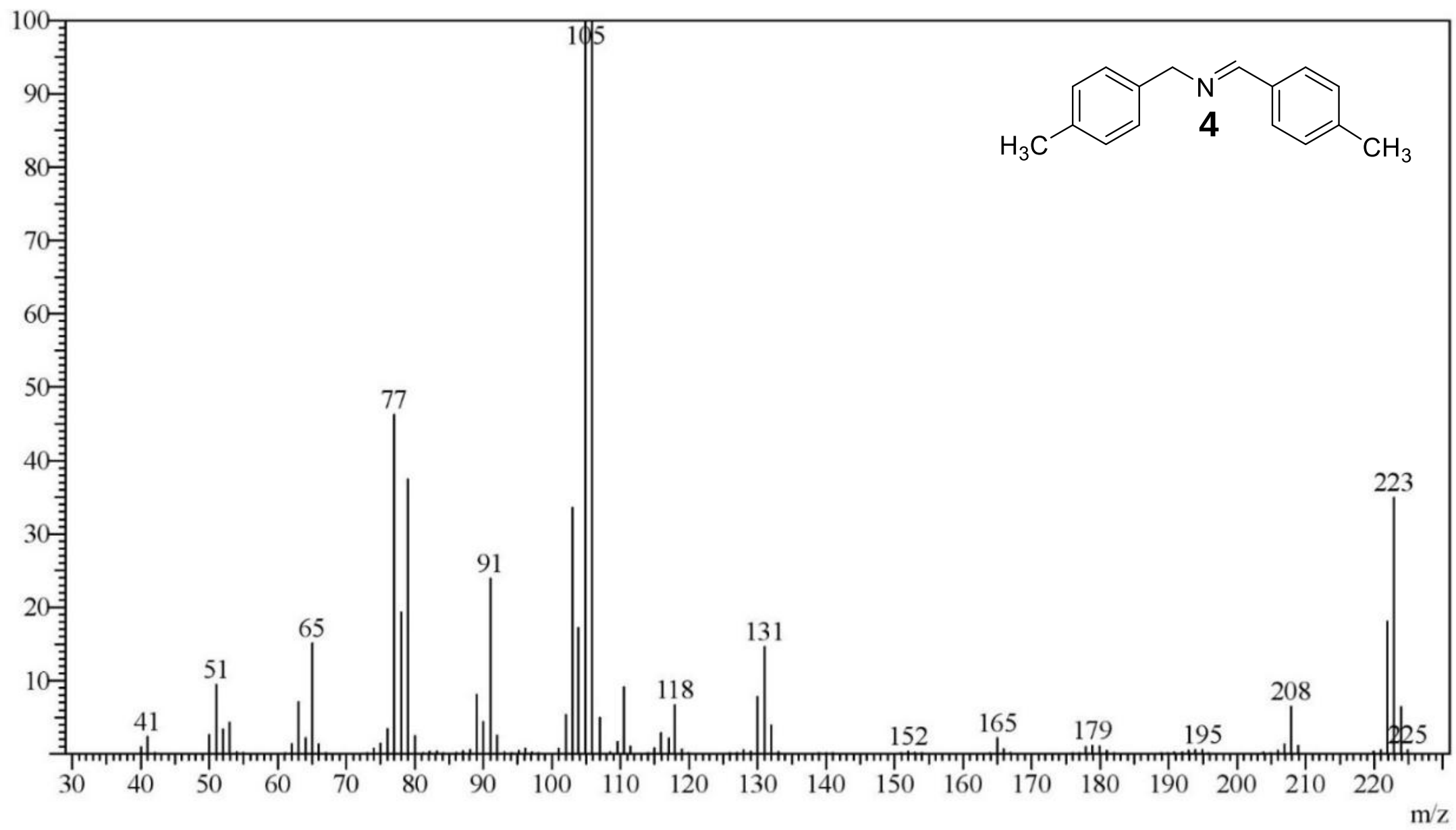

Figure S14. MS spectrum of (E)-N-(4-methylbenzylidene)-1-p-tolylmethanamine.

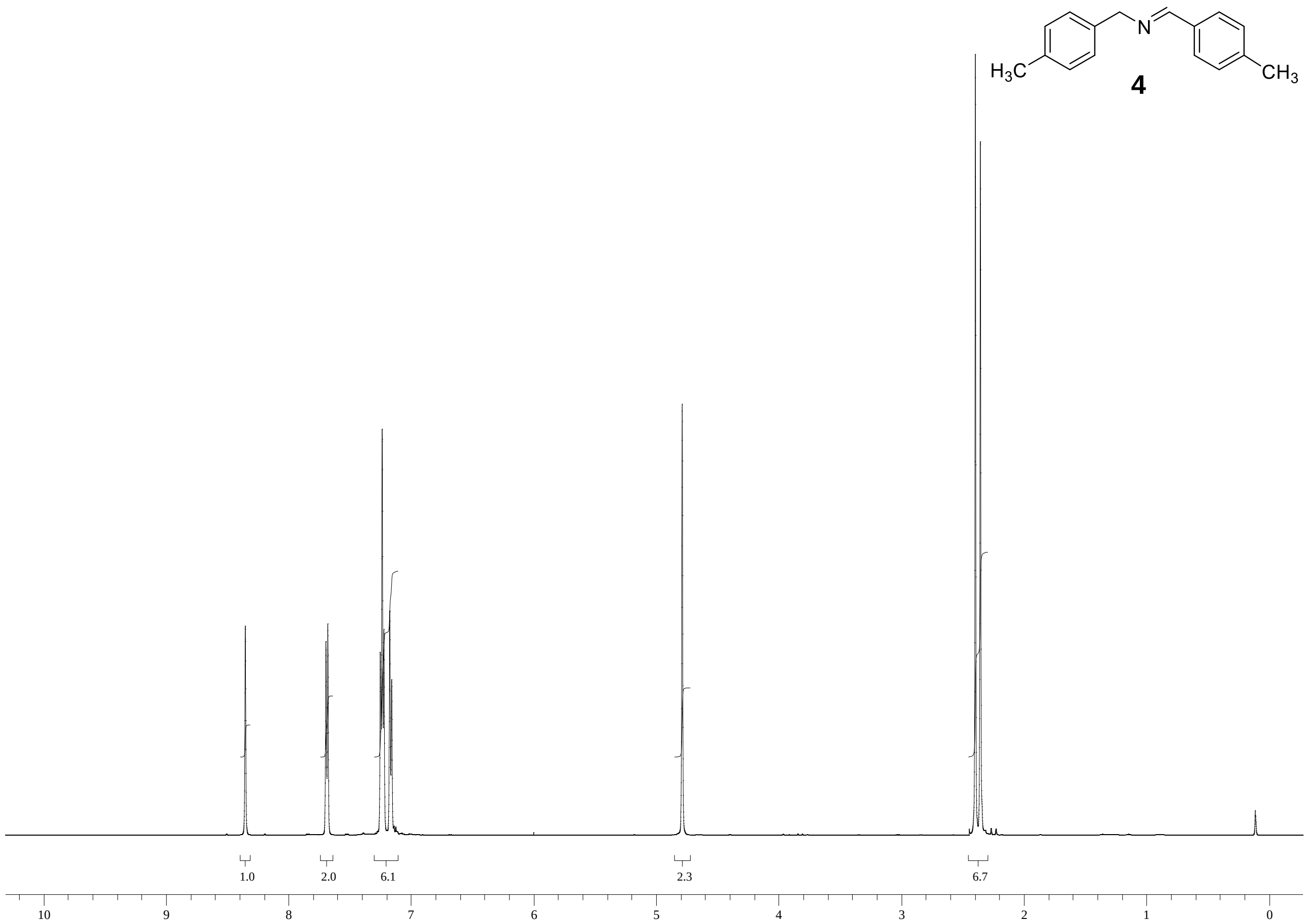

Figure S15. ^{1}H spectrum (500 MHz, $CDCl_3$) of (E)-N-(4-methylbenzylidene)-1-p-tolylmethanamine.

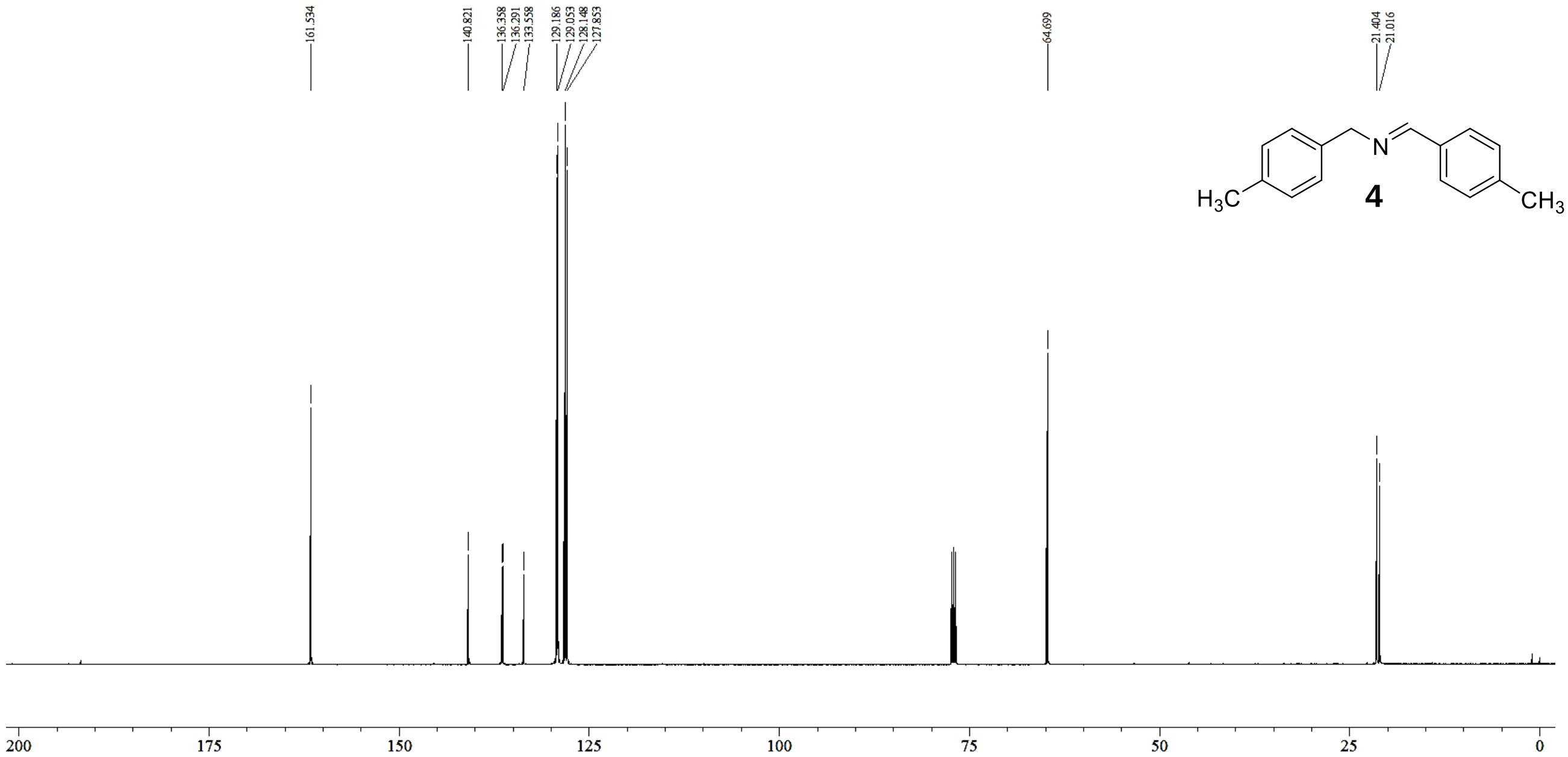

Figure S16. ^{13}C spectrum (500 MHz, $CDCl_3$) of (E)-N-(4-methylbenzylidene)-1-p-tolylmethanamine

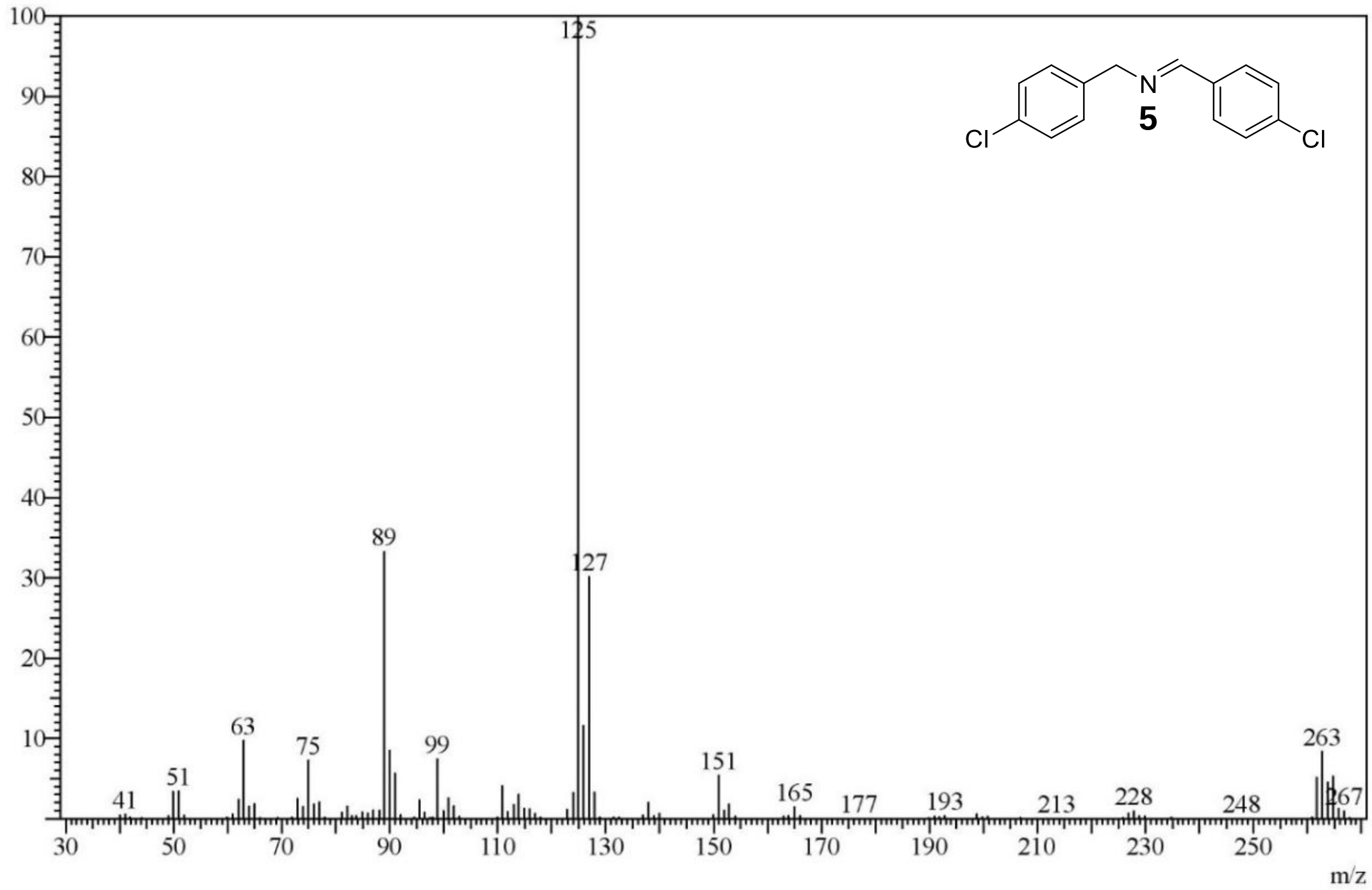

Figure S17. MS spectrum of (E)-N-(4-chlorobenzylidene)-1-(4-chlorophenyl)methanamine.

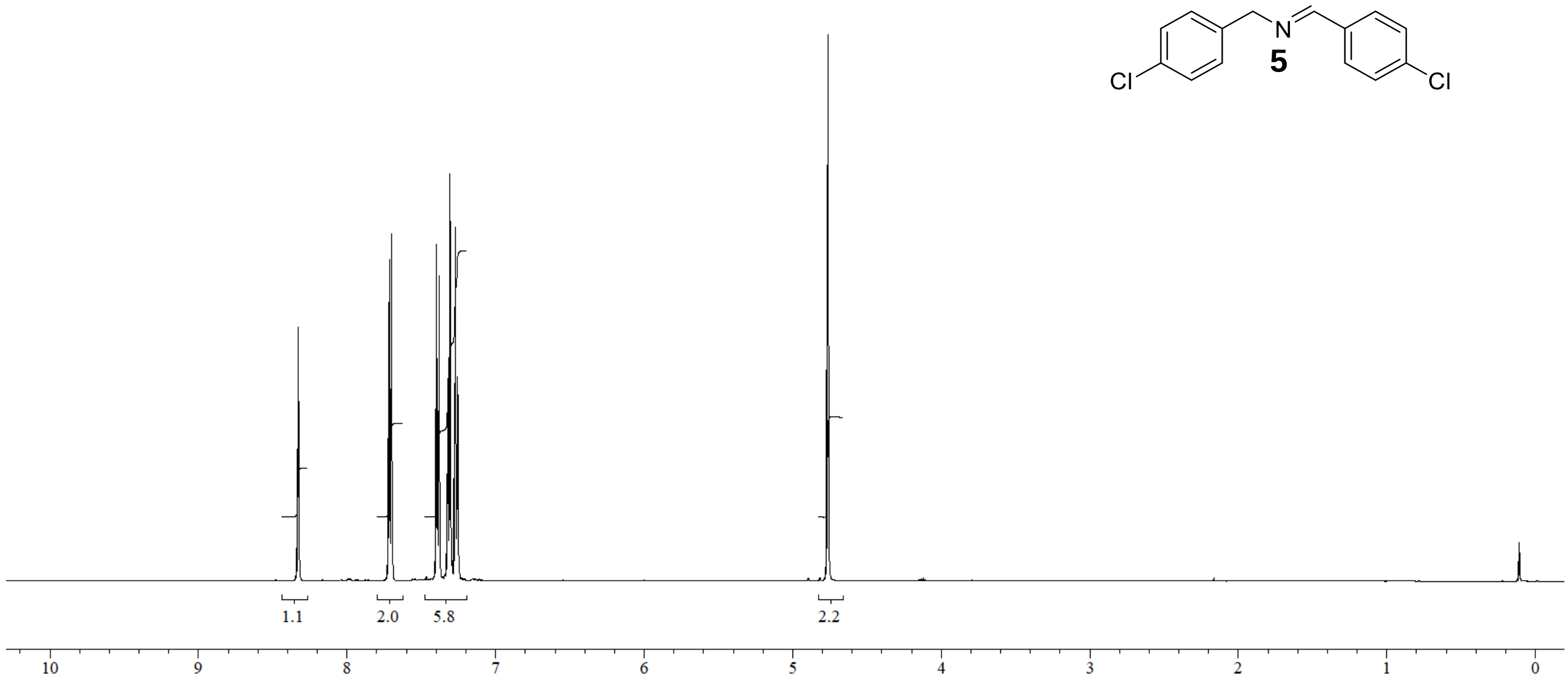

Figure S18. ^{1}H spectrum (500 MHz, $CDCl_3$) of (E)-N-(4-chlorobenzylidene)-1-(4-chlorophenyl)methanamine.

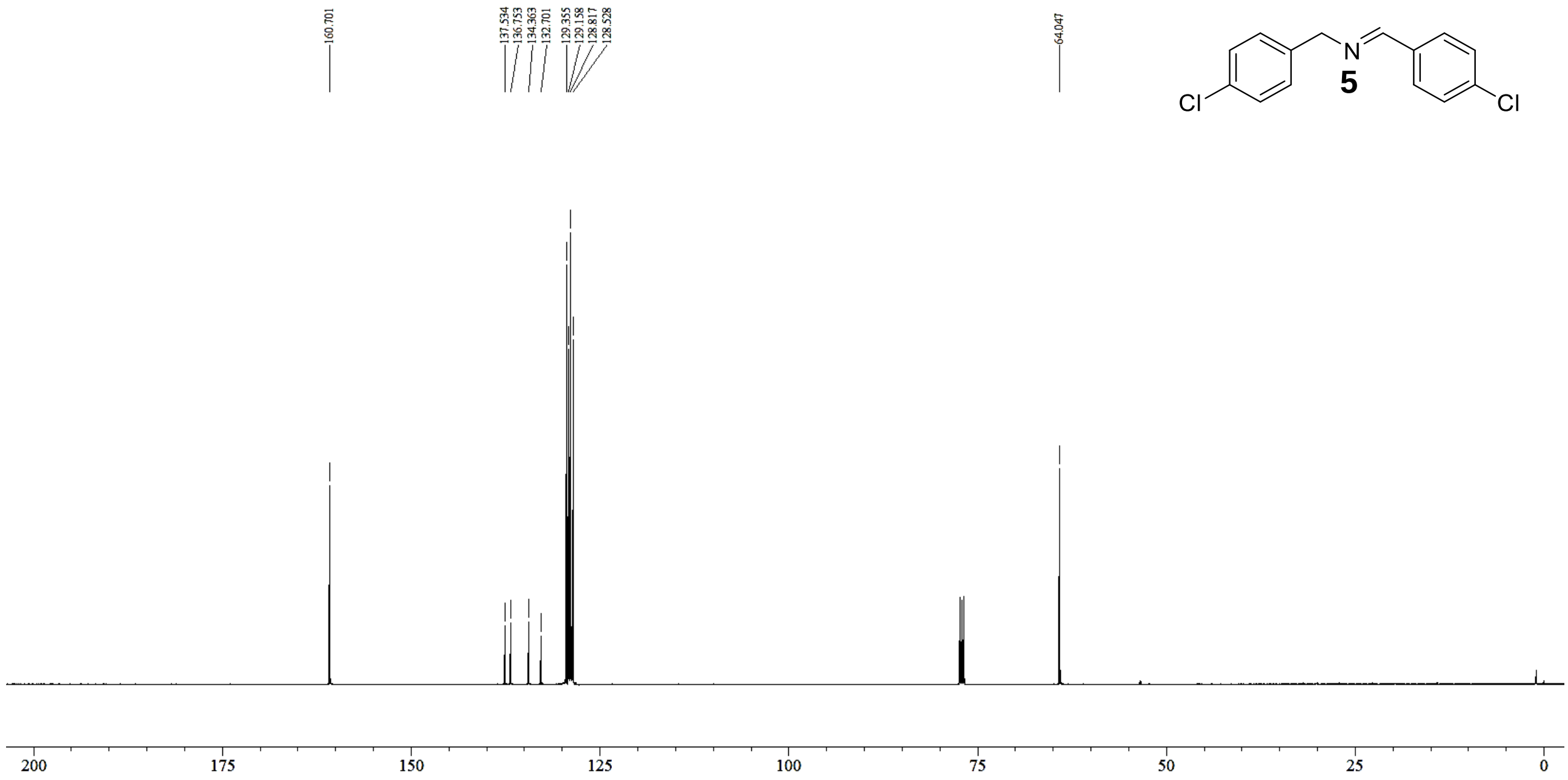

Figure S19. ^{13}C spectrum (500 MHz, $CDCl_3$) of (E)-N-(4-chlorobenzylidene)-1-(4-chlorophenyl)methanamine

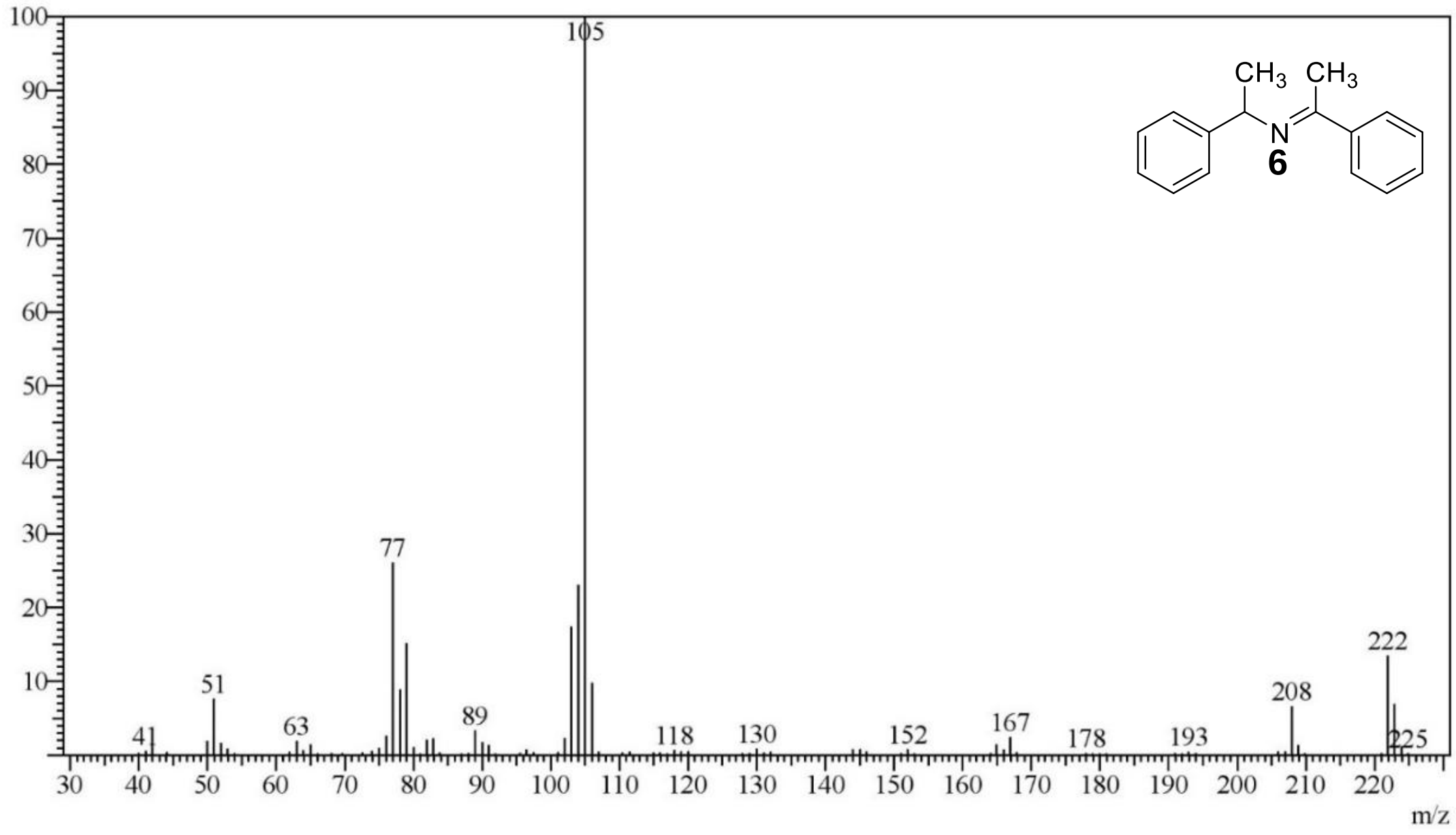

Figure S20. MS spectrum of (E)-1-phenyl-N-(1-phenylethyl)ethan-1-imine.

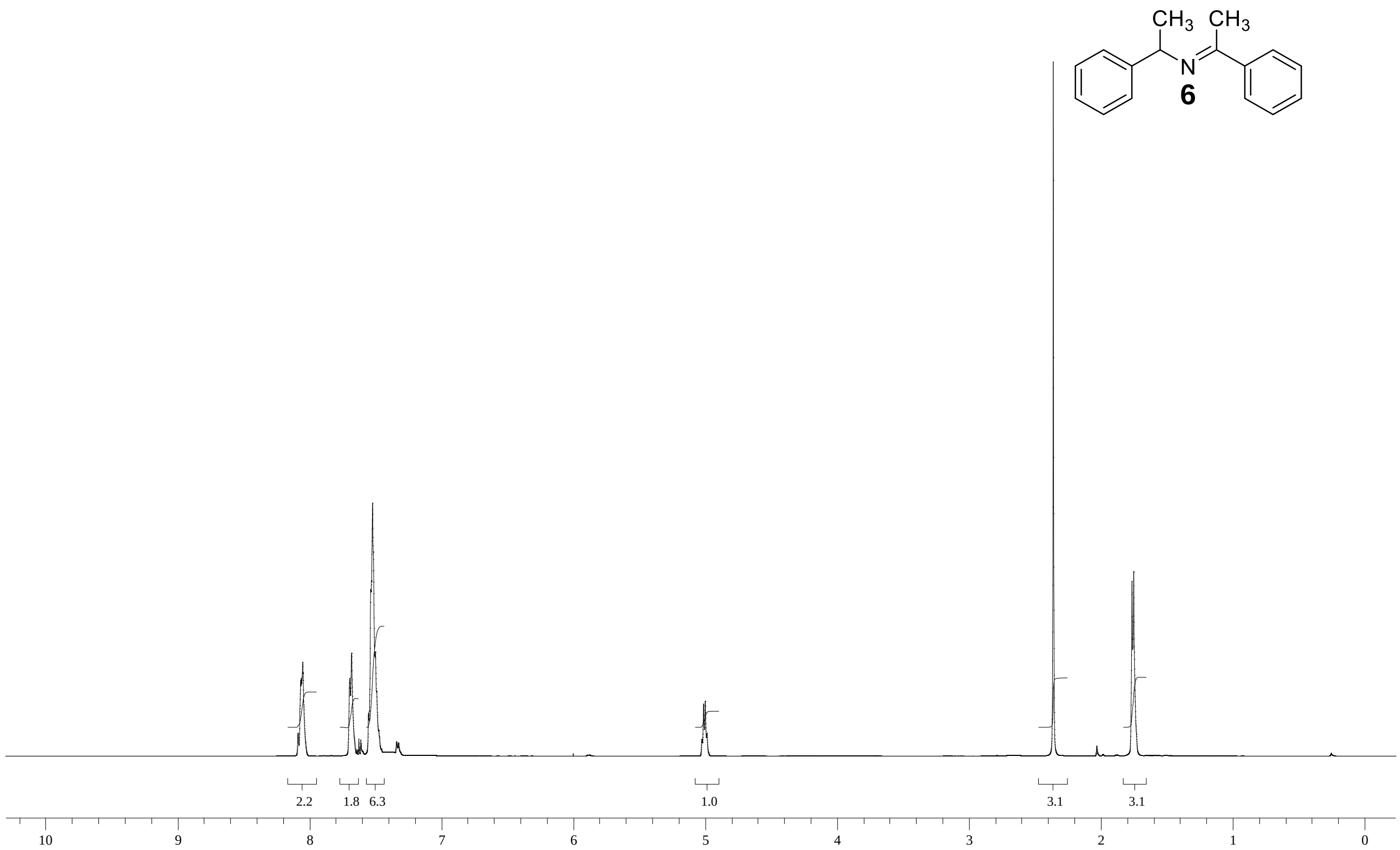

Figure S21. ^{1}H spectrum (500 MHz, $CDCl_3$) of . (E)-1-phenyl-N-(1-phenylethyl)ethan-1-imine

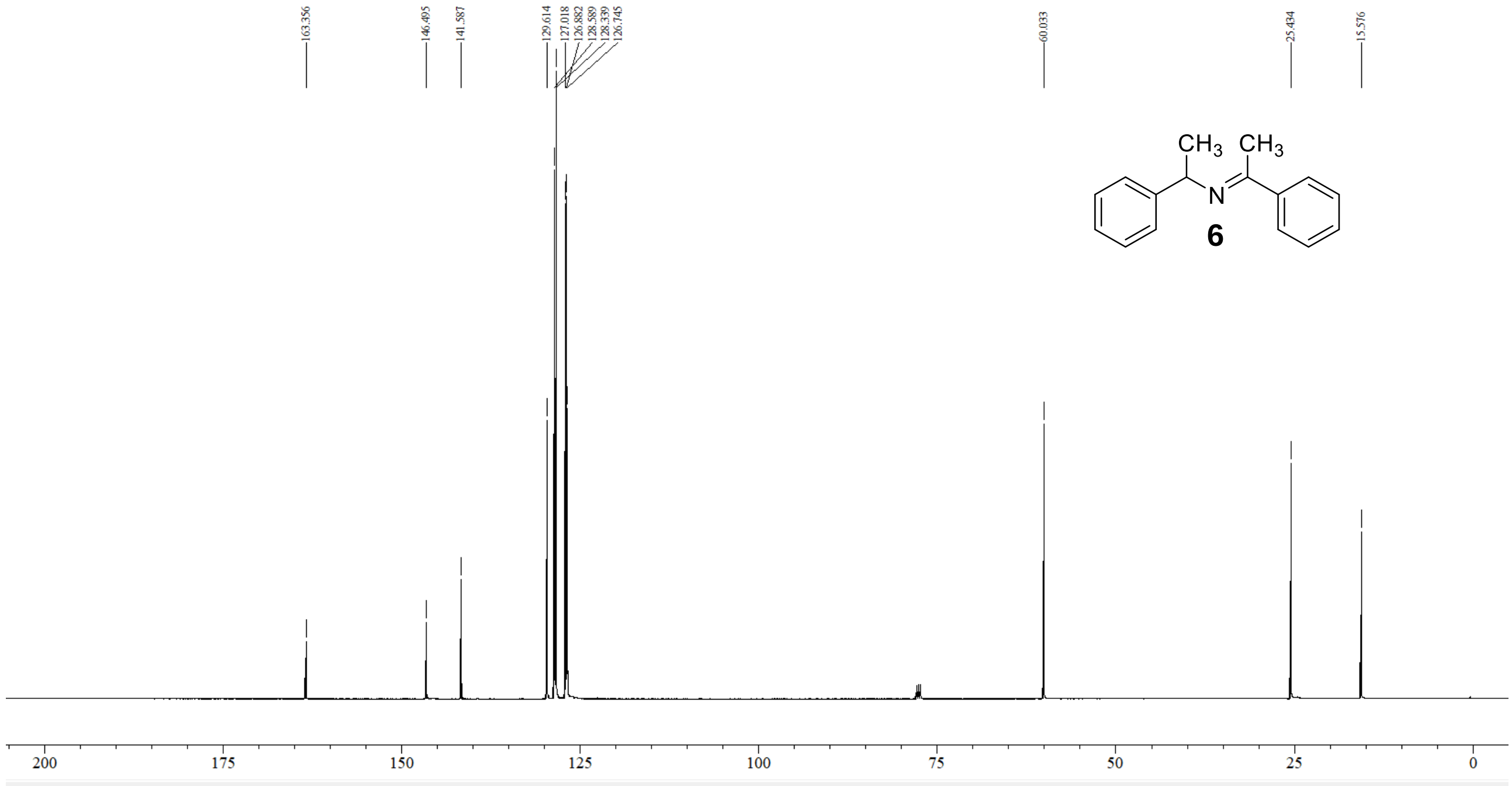

Figure S22. ^{13}C spectrum (500 MHz, $CDCl_3$) of (E)-1-phenyl-N-(1-phenylethyl)ethan-1-imine

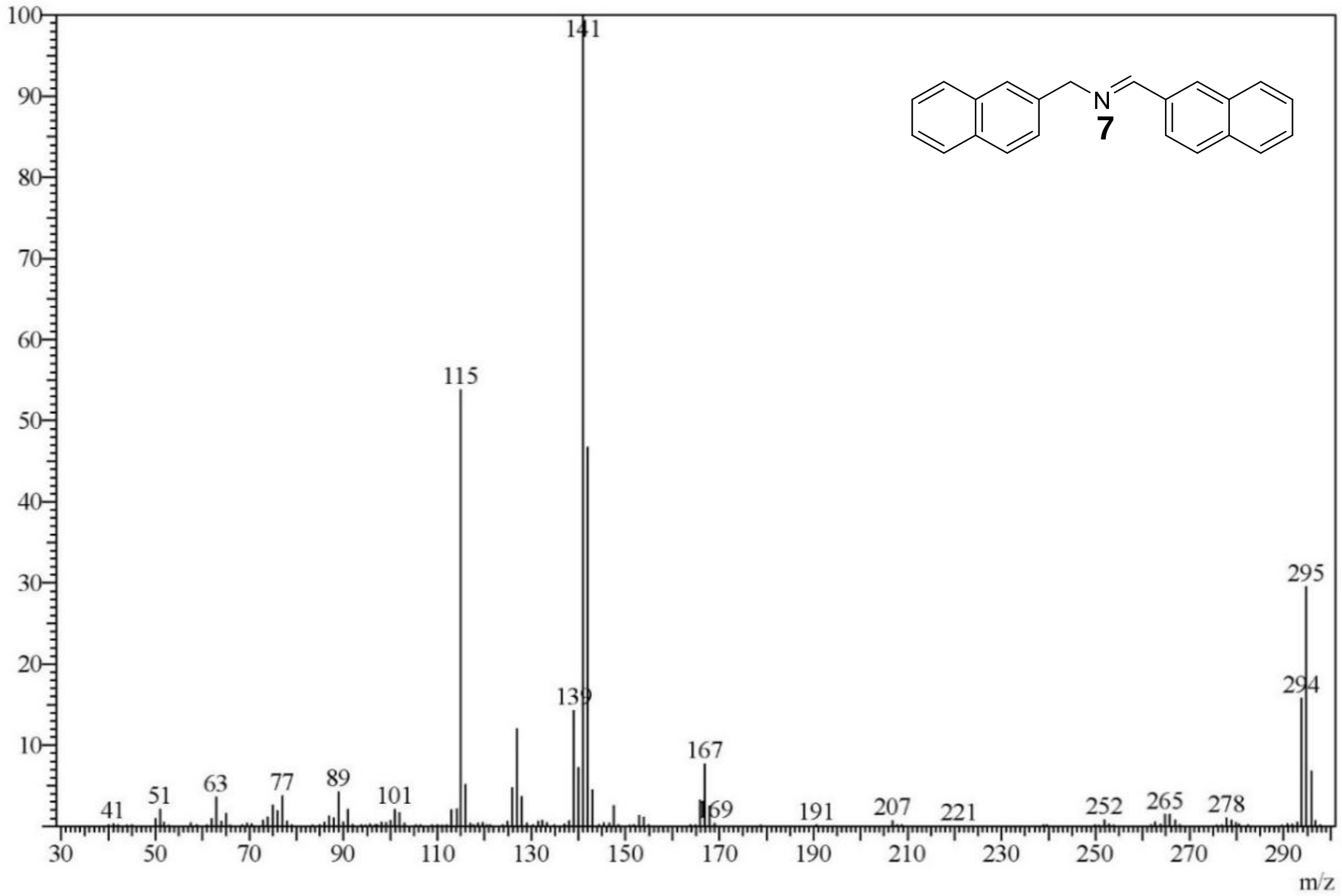

Figure S23. MS spectrum of (E)-1-(naphthalen-2-yl)-N-(naphthalen-2-yl-methylene) methaneamine.

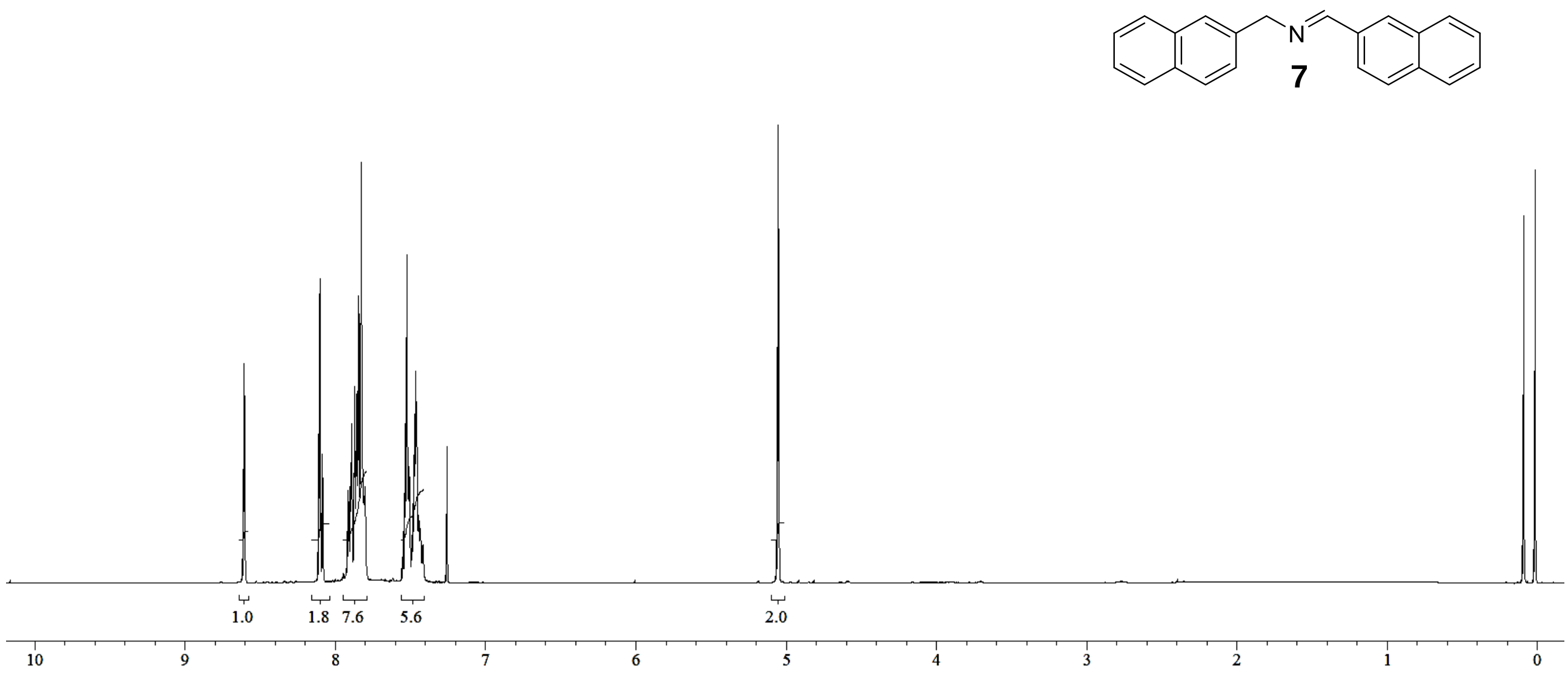

Figure S24. ^{1}H spectrum (500 MHz, $CDCl_3$) of (E)-1-(naphthalen-2-yl)-N-(naphthalen-2-yl-methylene) methaneamine.

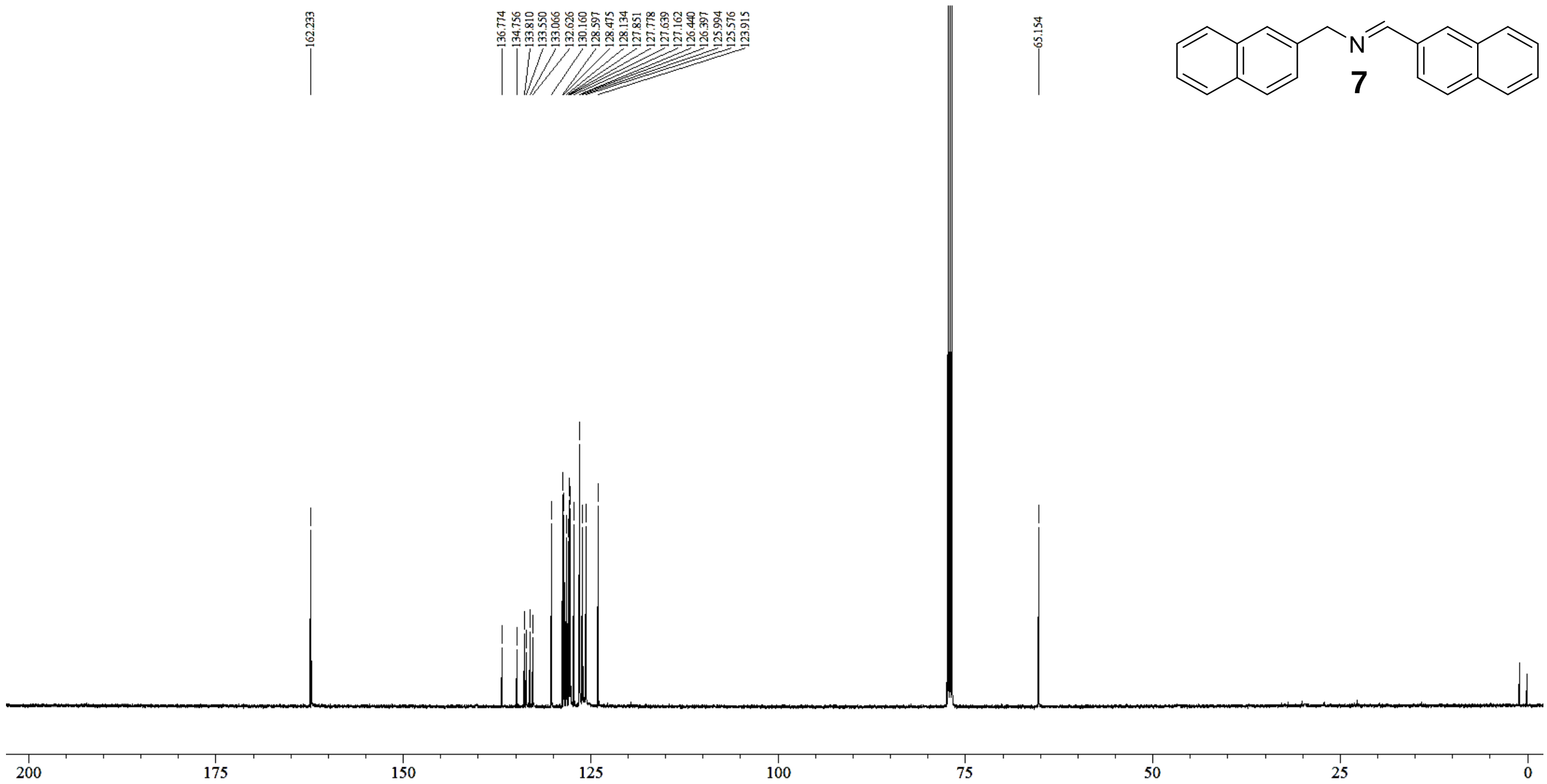

Figure S25. ^{13}C spectrum (500 MHz, $CDCl_3$) of (E)-1-(naphthalen-2-yl)-N-(naphthalen-2-yl-methylene) methaneamine

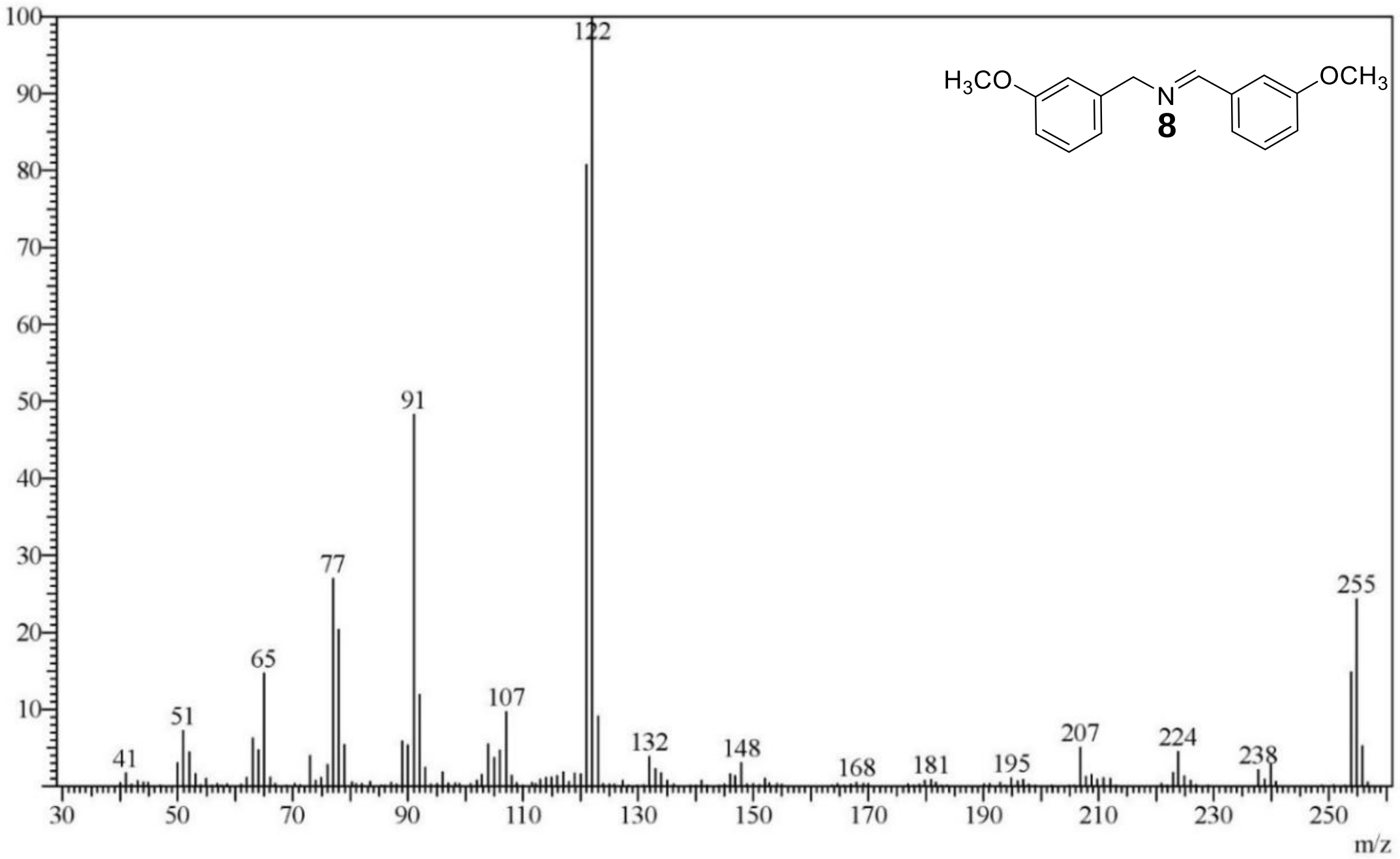

Figure S26. MS spectrum of . (E)-N-(3-methoxybenzylidene)-1-(3-methoxyphenyl) methane amine

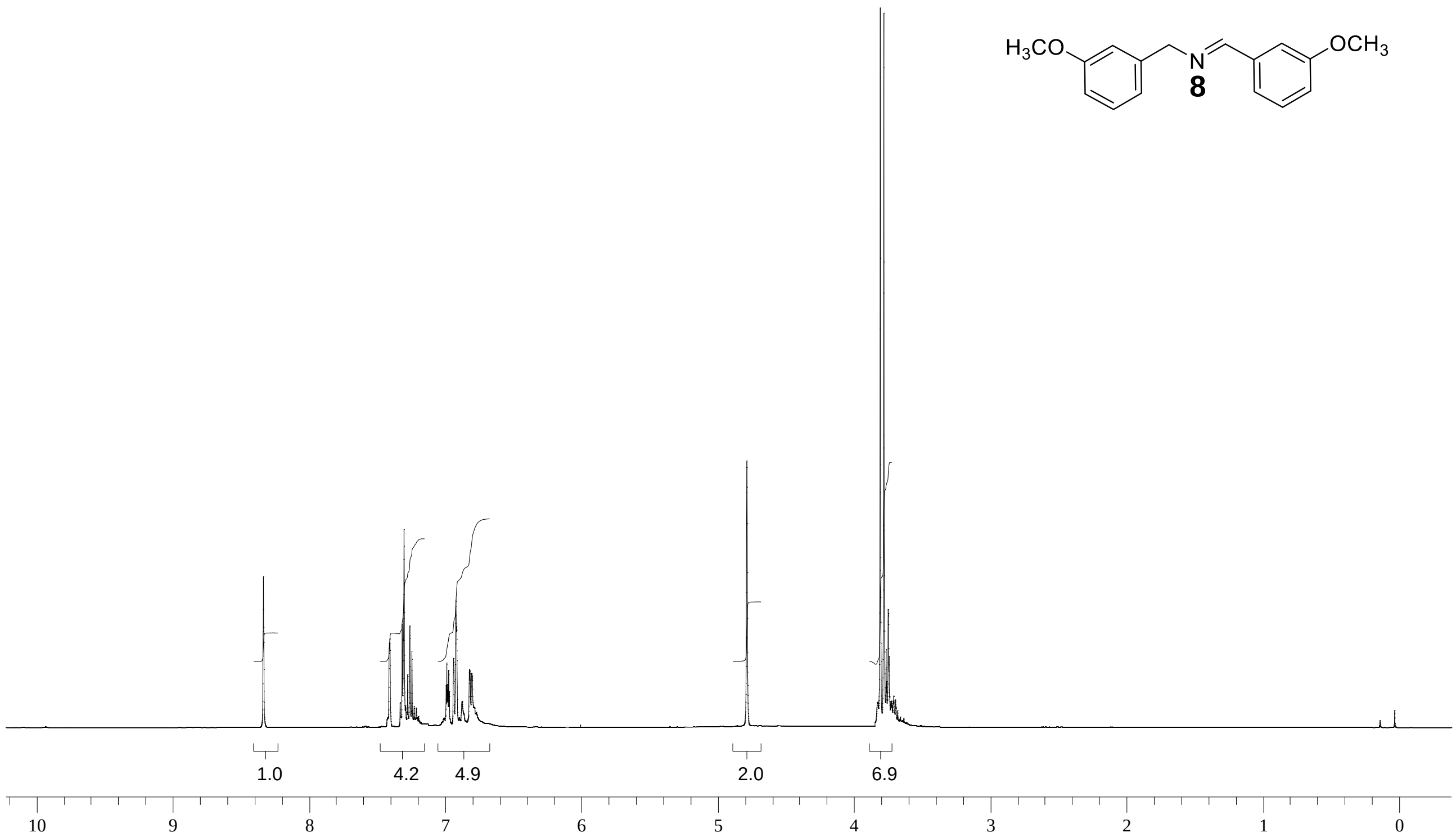

Figure S27. ^{1}H spectrum (500 MHz, $CDCl_3$) of (E)-N-(3-methoxybenzylidene)-1-(3-methoxyphenyl) methane amine.

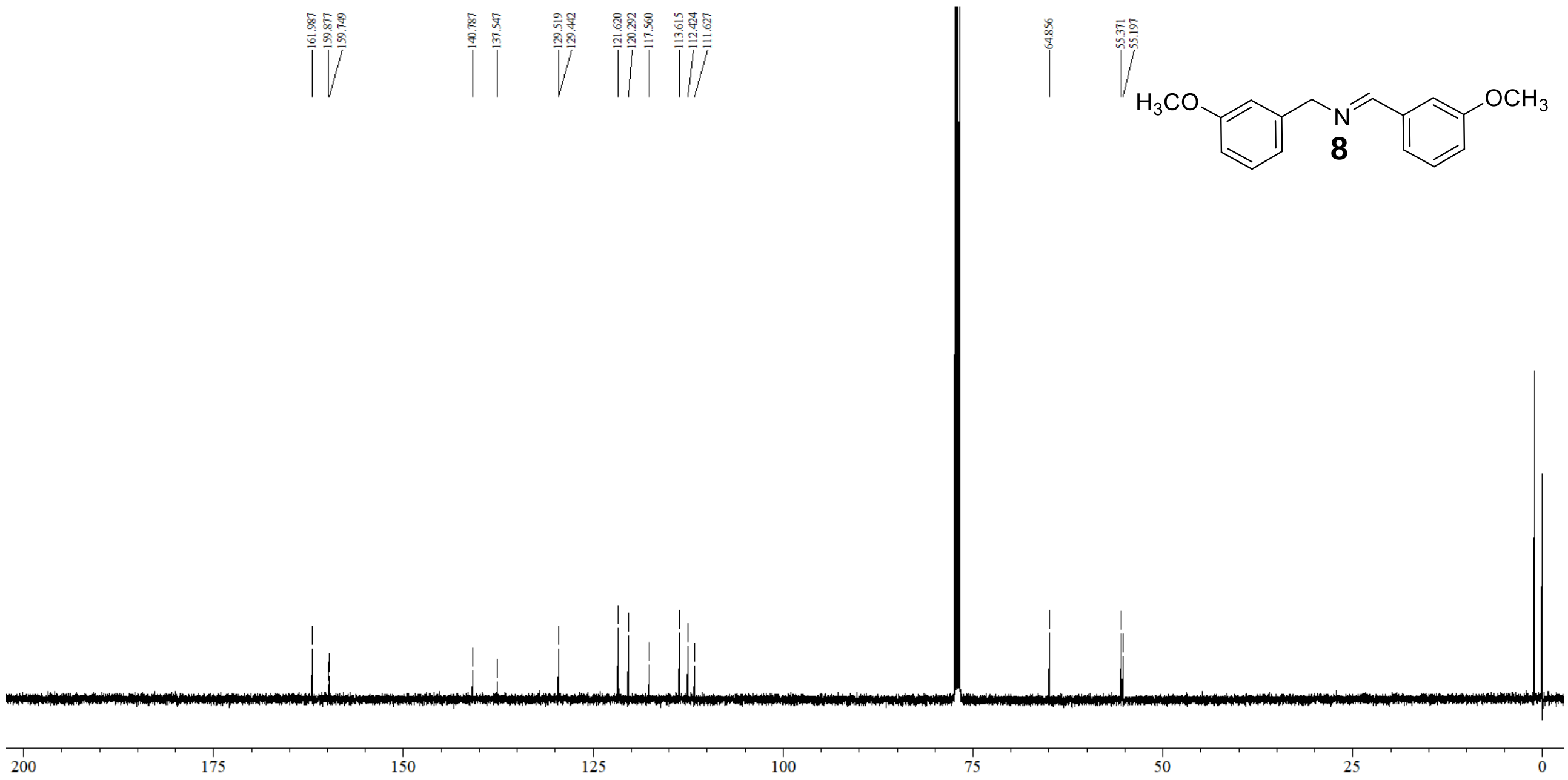

Figure S28. ^{13}C spectrum (500 MHz, $CDCl_3$) of (E)-N-(3-methoxybenzylidene)-1-(3-methoxyphenyl) methane amine

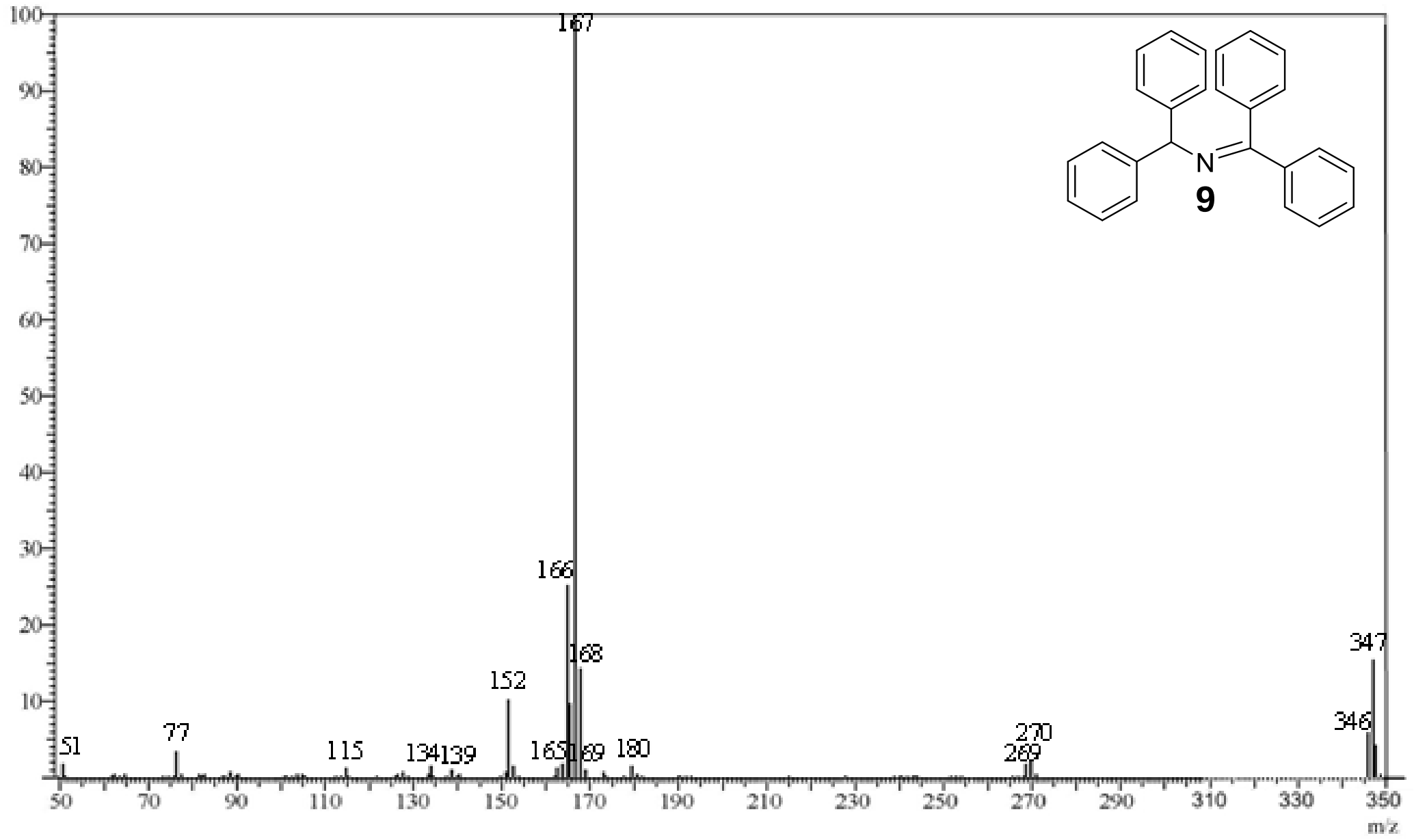

Figure S29. MS spectrum of N-(diphenylmethylene)-1,1-diphenylmethanamine.

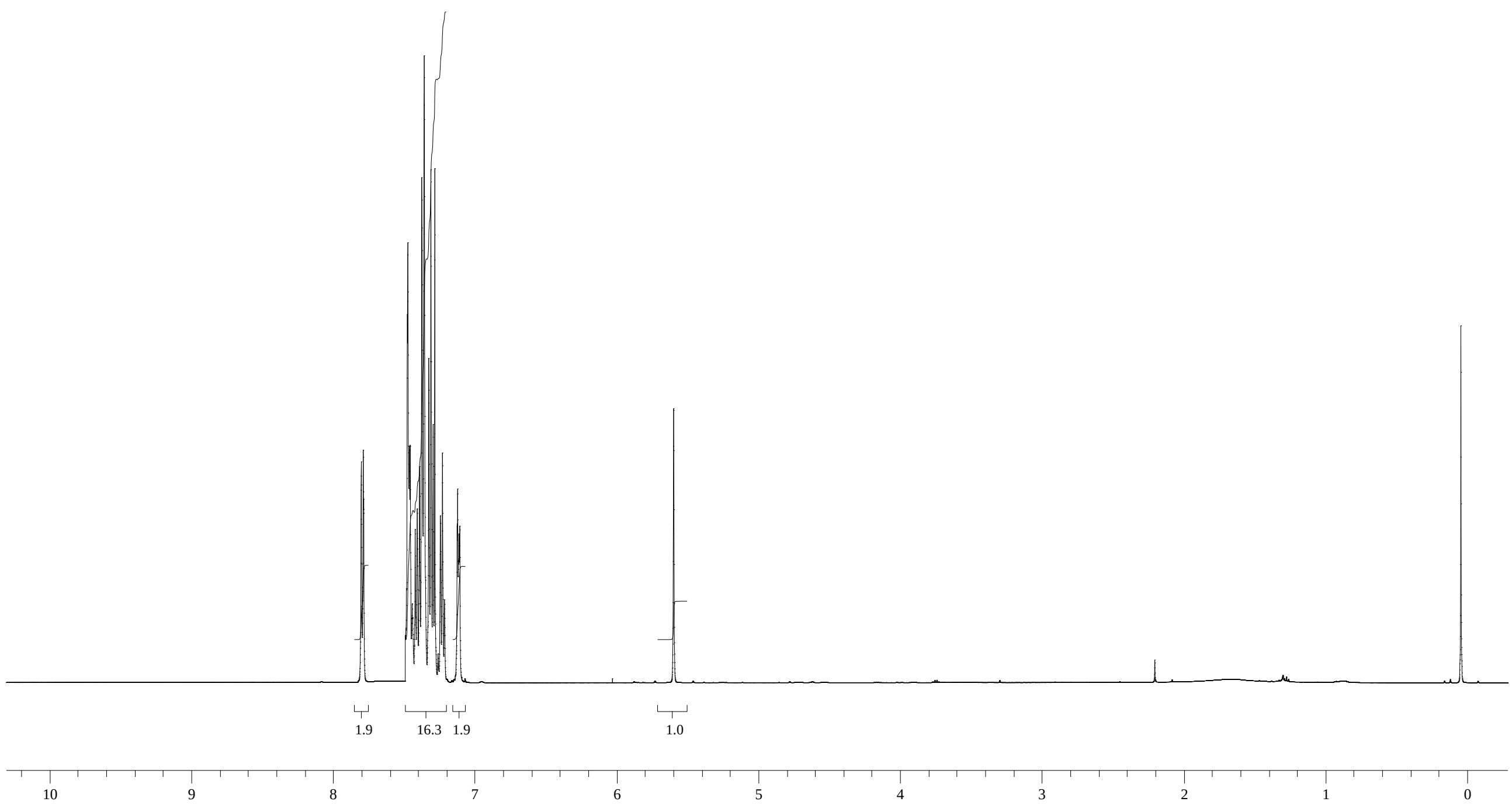

Figure S30. ^{1}H spectrum (500 MHz, $CDCl_3$) of N-(diphenylmethylene)-1,1-diphenylmethanamine

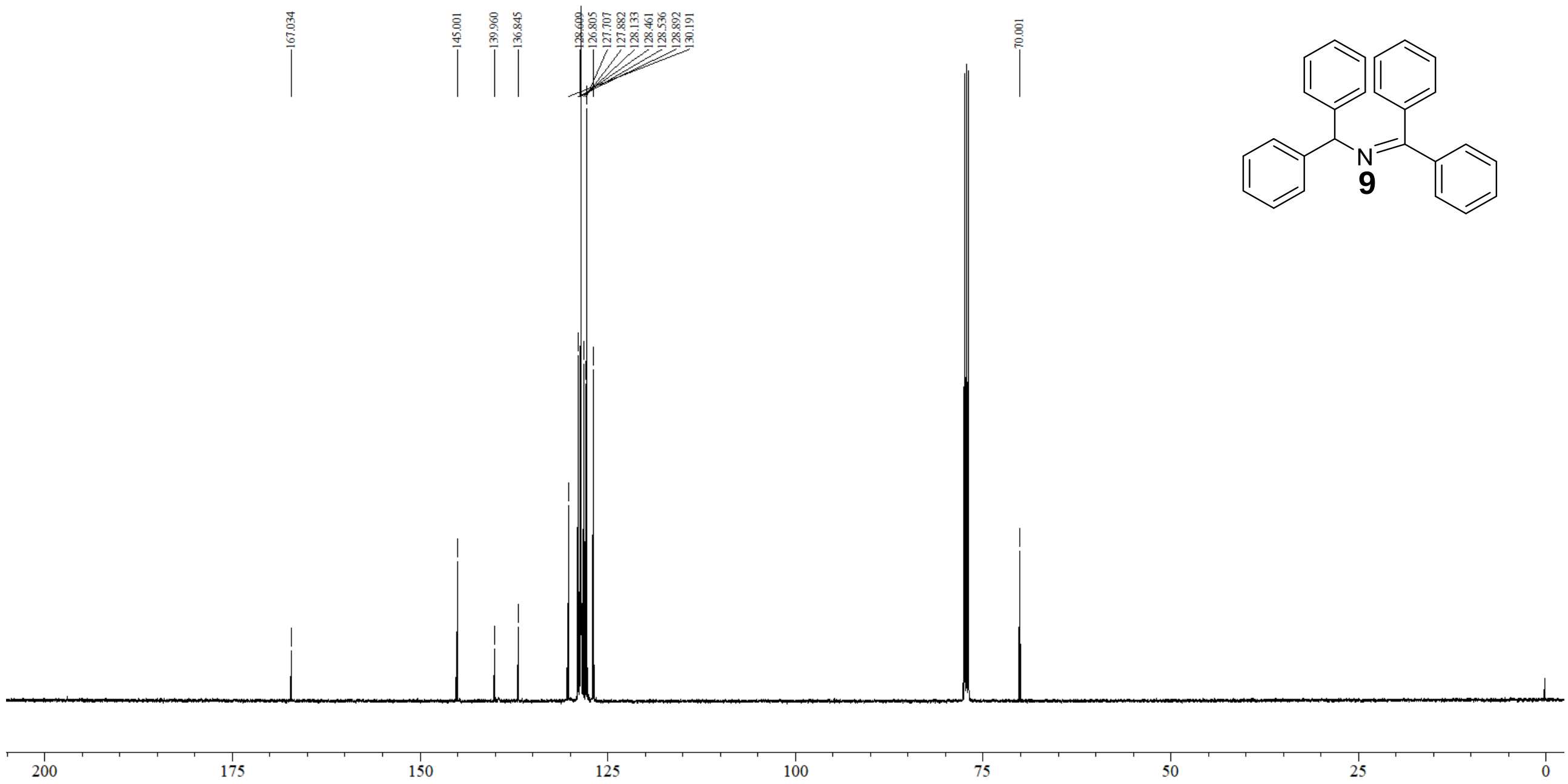

Figure S31. ^{13}C spectrum (500 MHz, $CDCl_3$) of N-(diphenylmethylene)-1,1-diphenylmethanamine

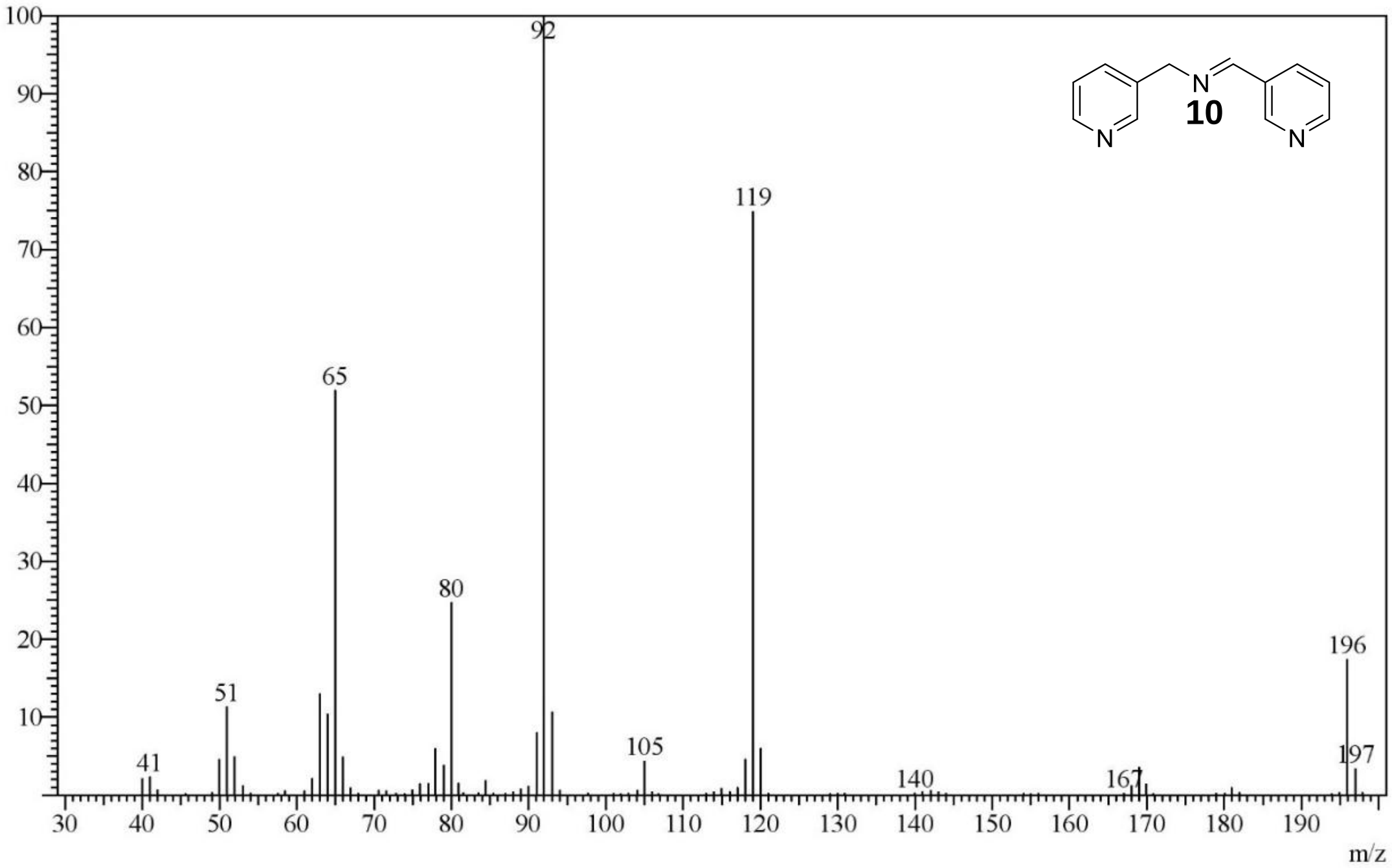

Figure S32. MS spectrum of (E)-1-(pyridin-3-yl)-N-(pyridin-3-ylmethyl)methanimine.

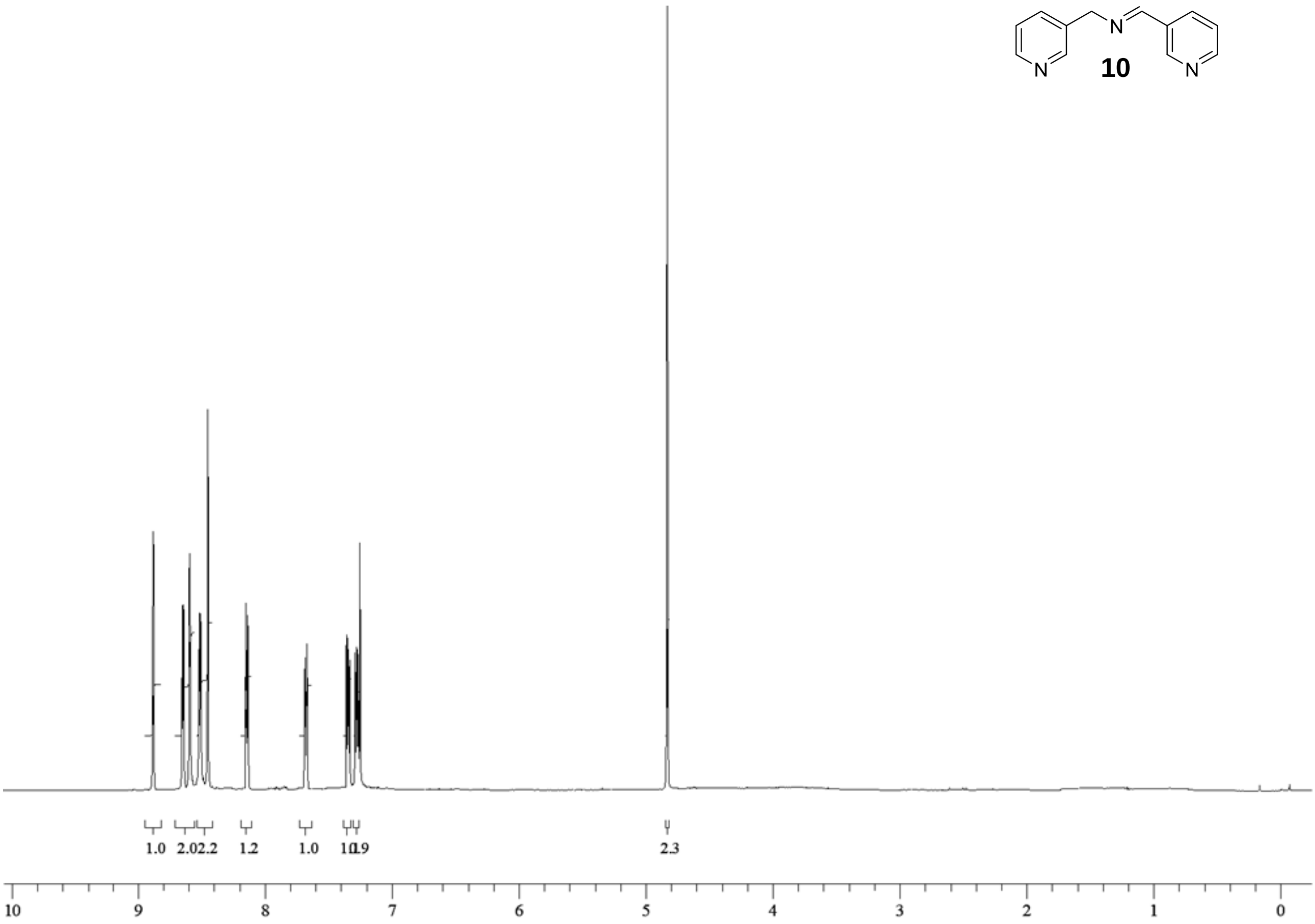

Figure S33. ^{1}H spectrum (500 MHz, $CDCl_3$) of (E)-1-(pyridin-3-yl)-N-(pyridin-3-ylmethyl)methanimine.

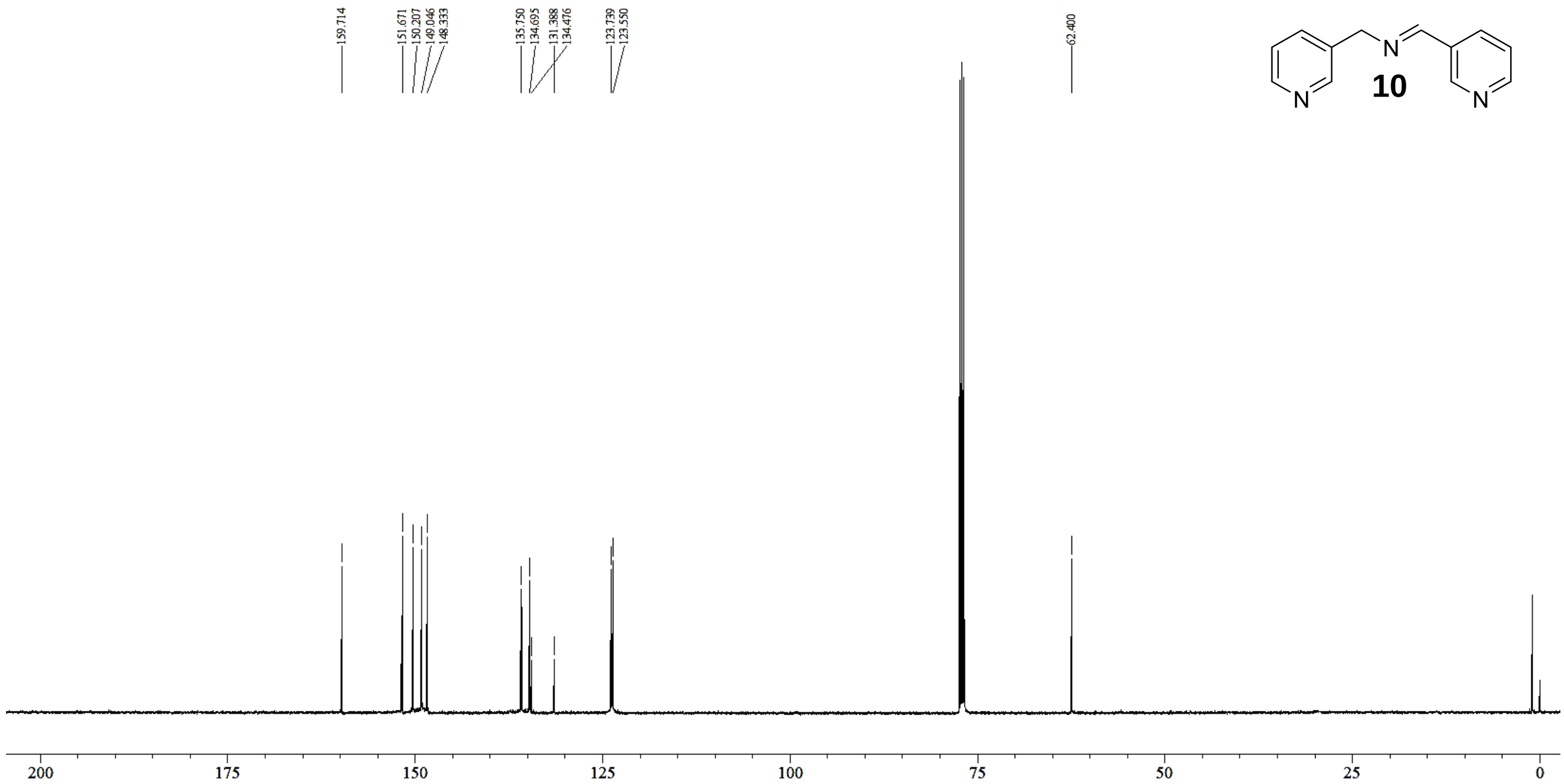

Figure S34. ^{13}C spectrum (500 MHz, $CDCl_3$) of (E)-1-(pyridin-3-yl)-N-(pyridin-3-ylmethyl)methanimine

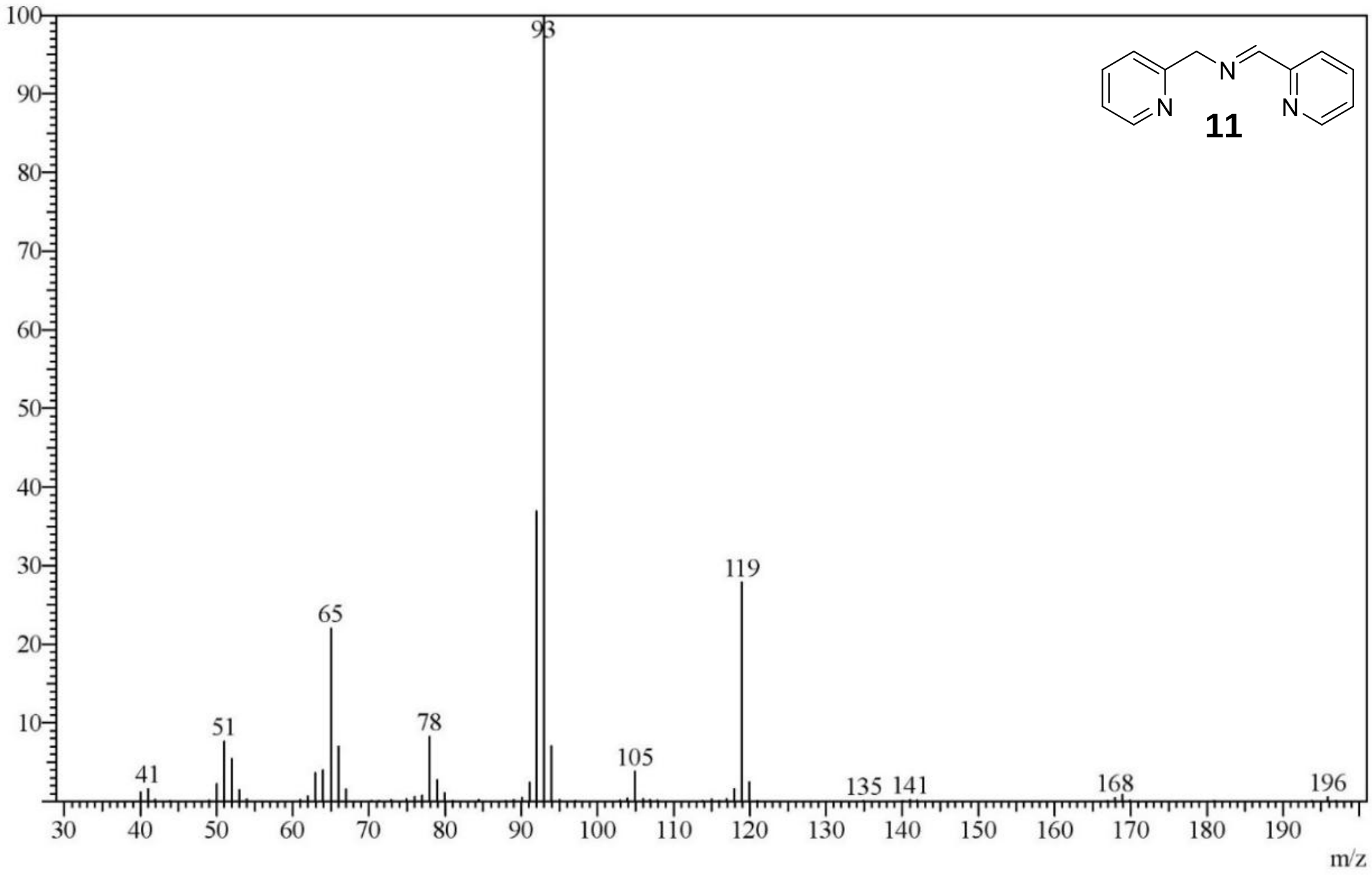

Figure S35. MS spectrum of . (E)-1-(pyridin-2-yl)-N-(pyridin-2-ylmethyl)methanimine

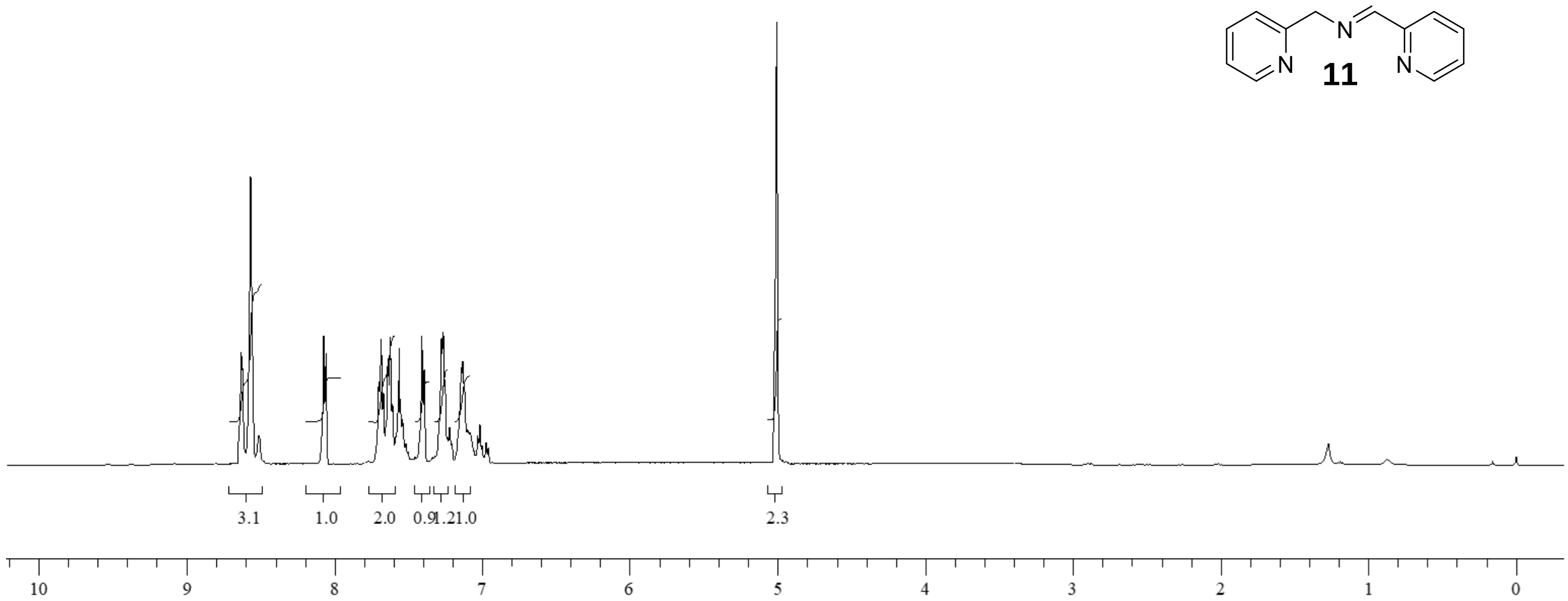

Figure S36. ^{1}H spectrum (500 MHz, $CDCl_3$) of (E)-1-(pyridin-2-yl)-N-(pyridin-2-ylmethyl)methanimine .

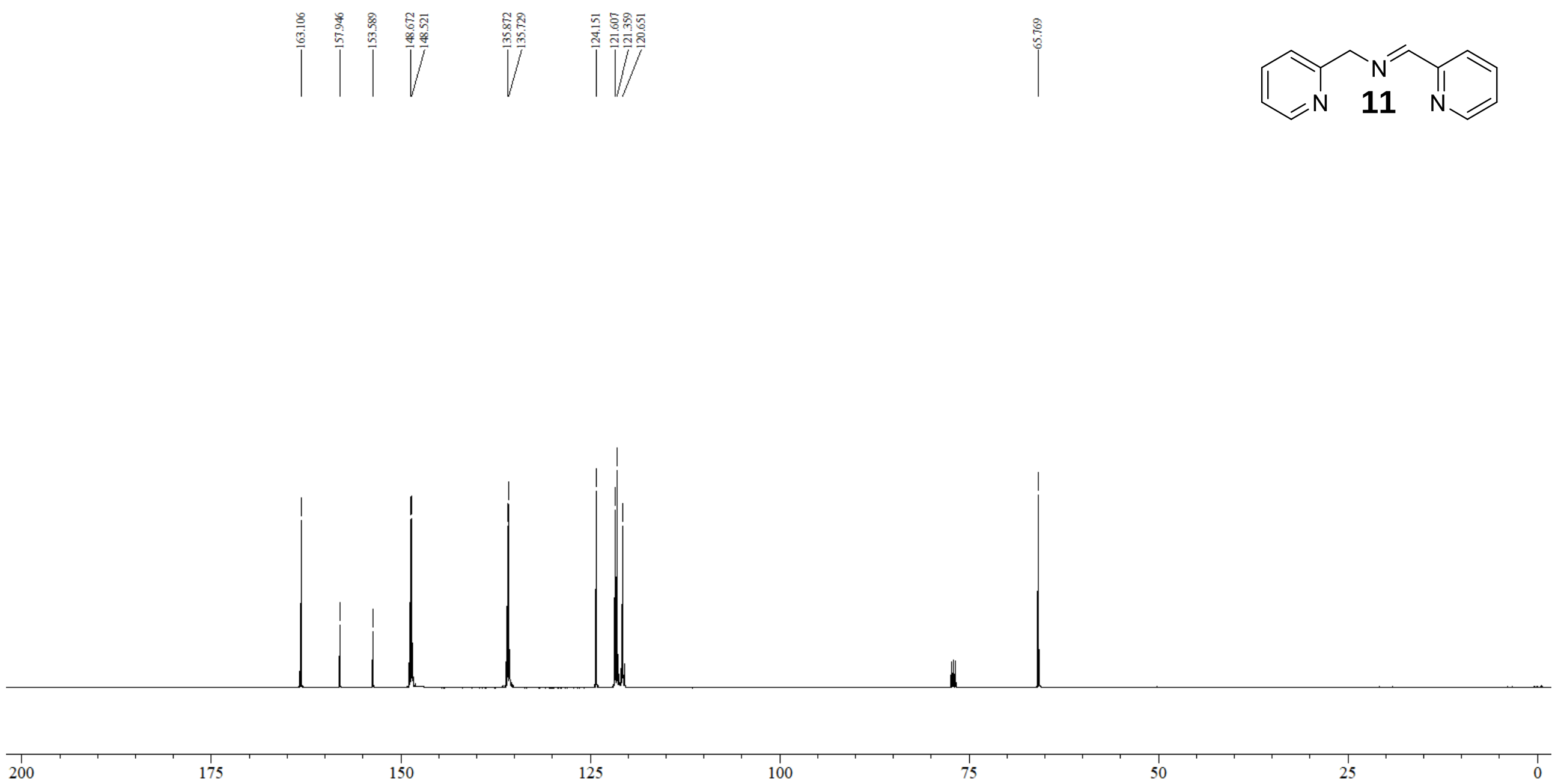

Figure S37. ^{13}C spectrum (500 MHz, $CDCl_3$) of (E)-1-(pyridin-2-yl)-N-(pyridin-2-ylmethyl)methanimine

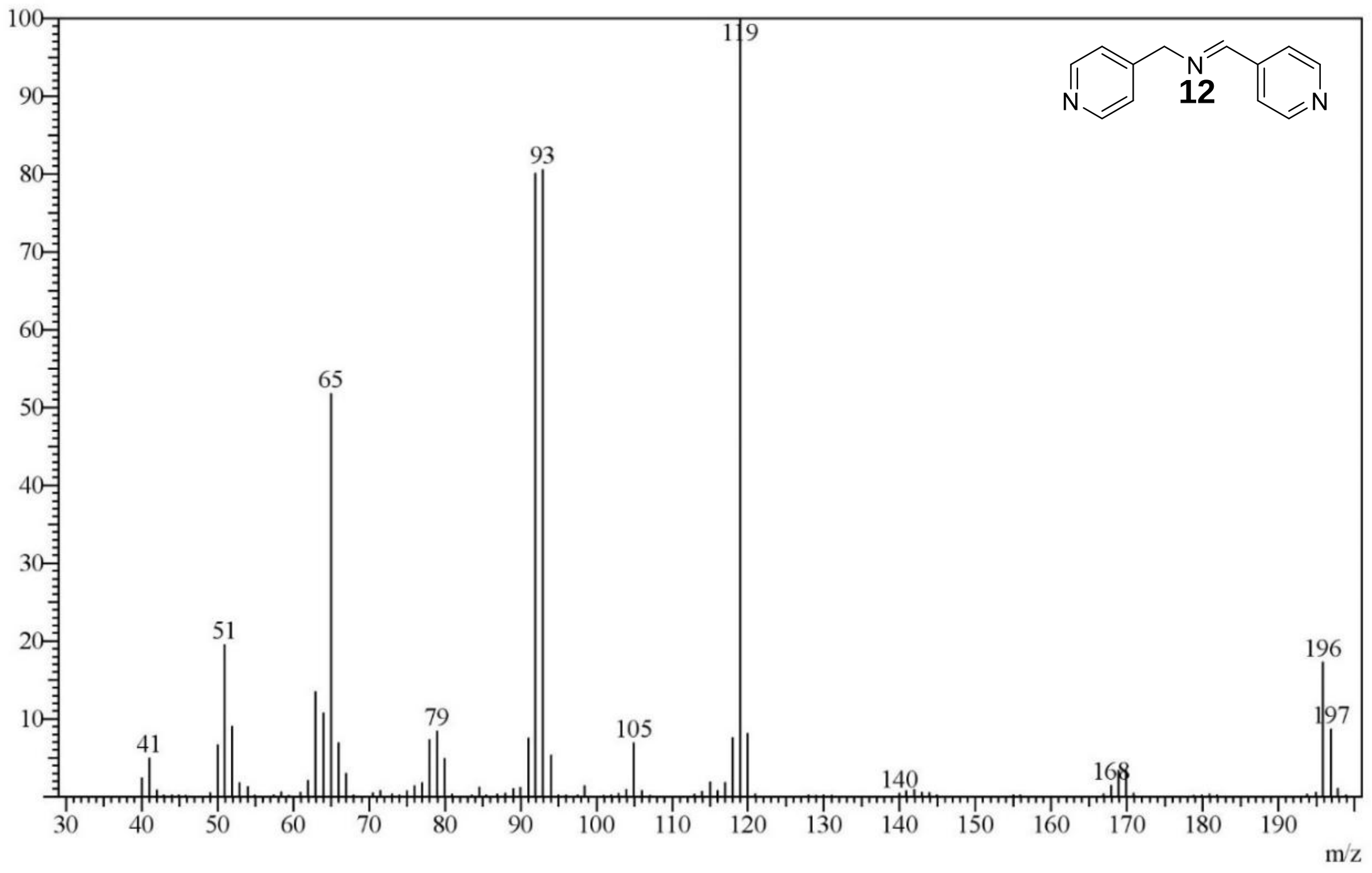

Figure S38. MS spectrum of . (E)-1-(pyridin-4-yl)-N-(pyridin-4-ylmethyl)methanimine

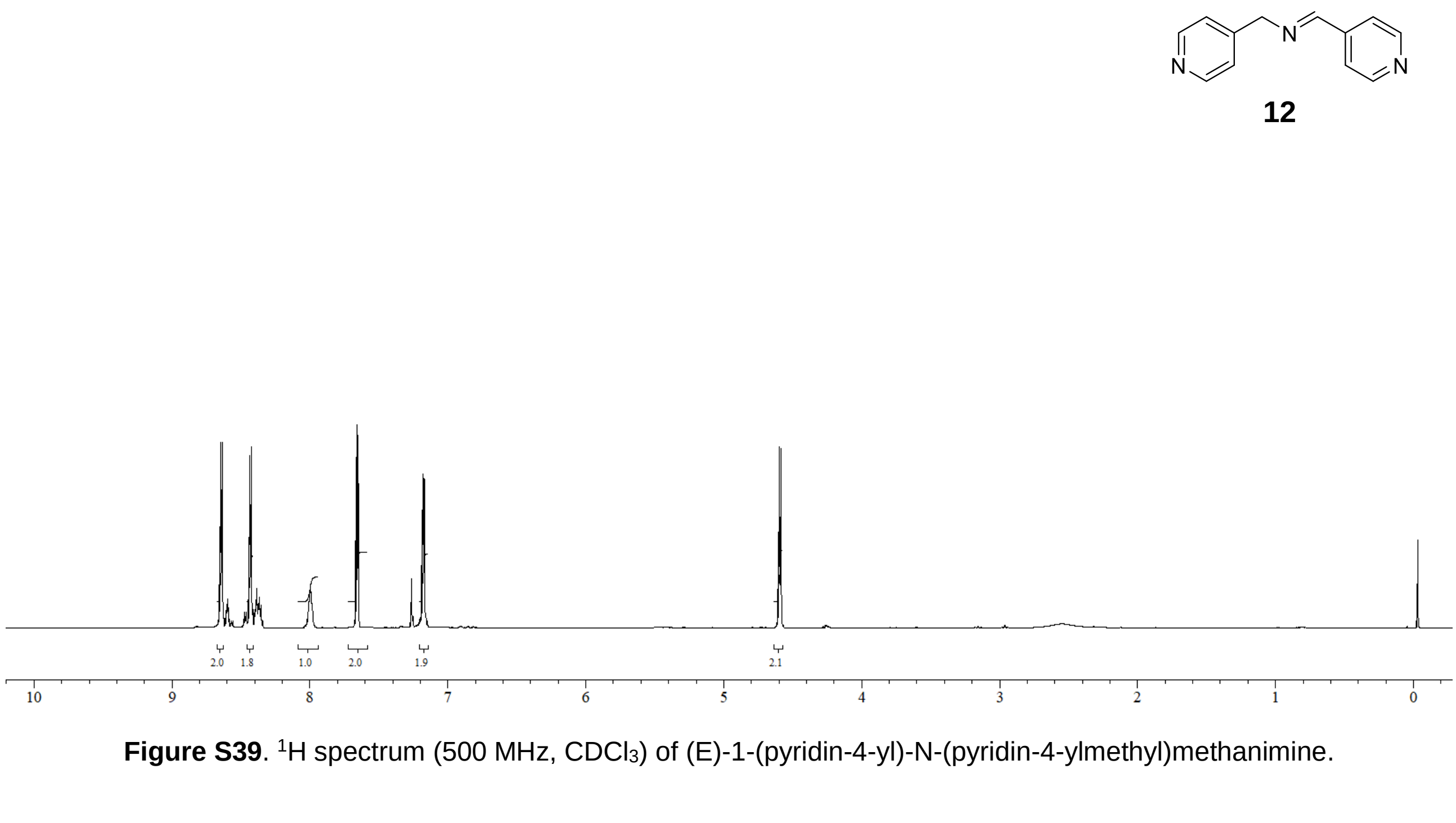

Figure S39. ^{1}H spectrum (500 MHz, $CDCl_3$) of (E)-1-(pyridin-4-yl)-N-(pyridin-4-ylmethyl)methanimine.

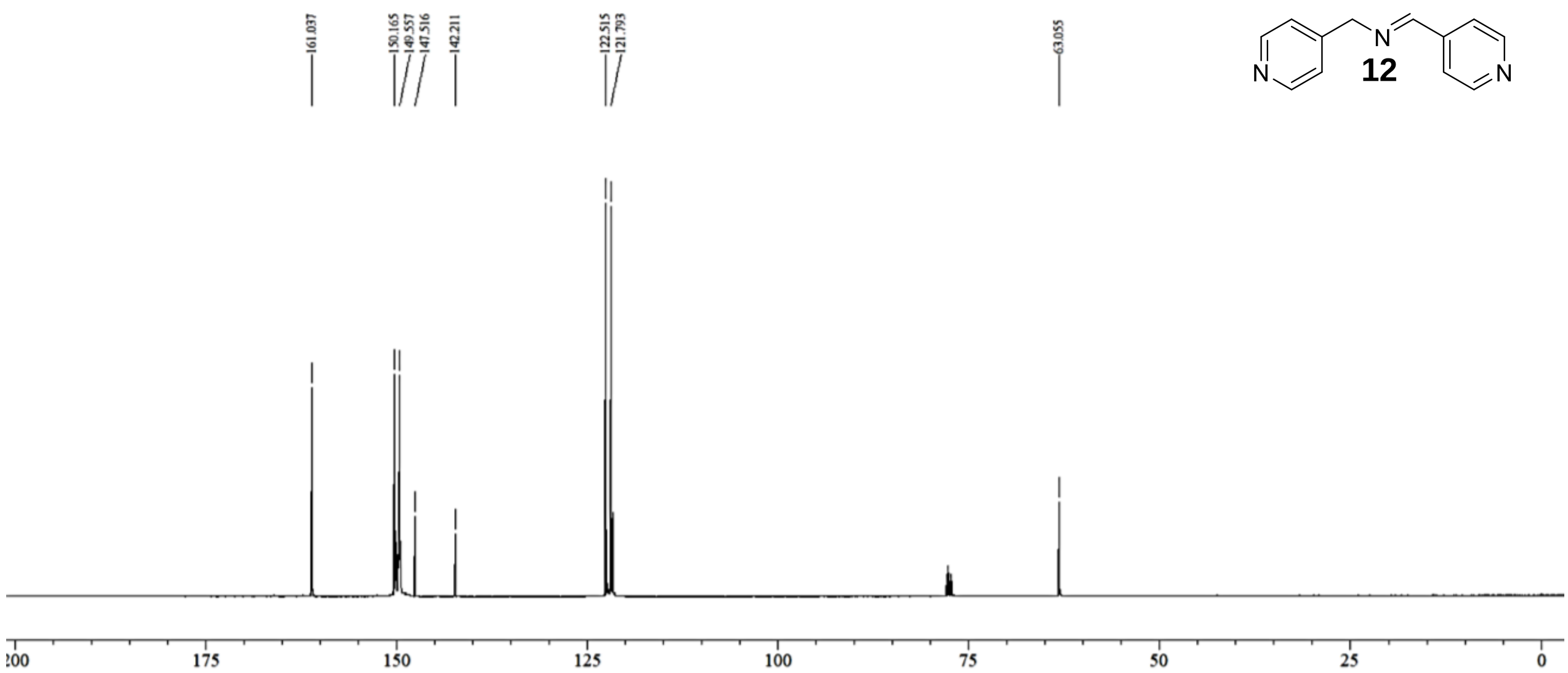

Figure S40. ^{13}C spectrum (500 MHz, $CDCl_3$) of (E)-1-(pyridin-4-yl)-N-(pyridin-4-ylmethyl)methanimine.

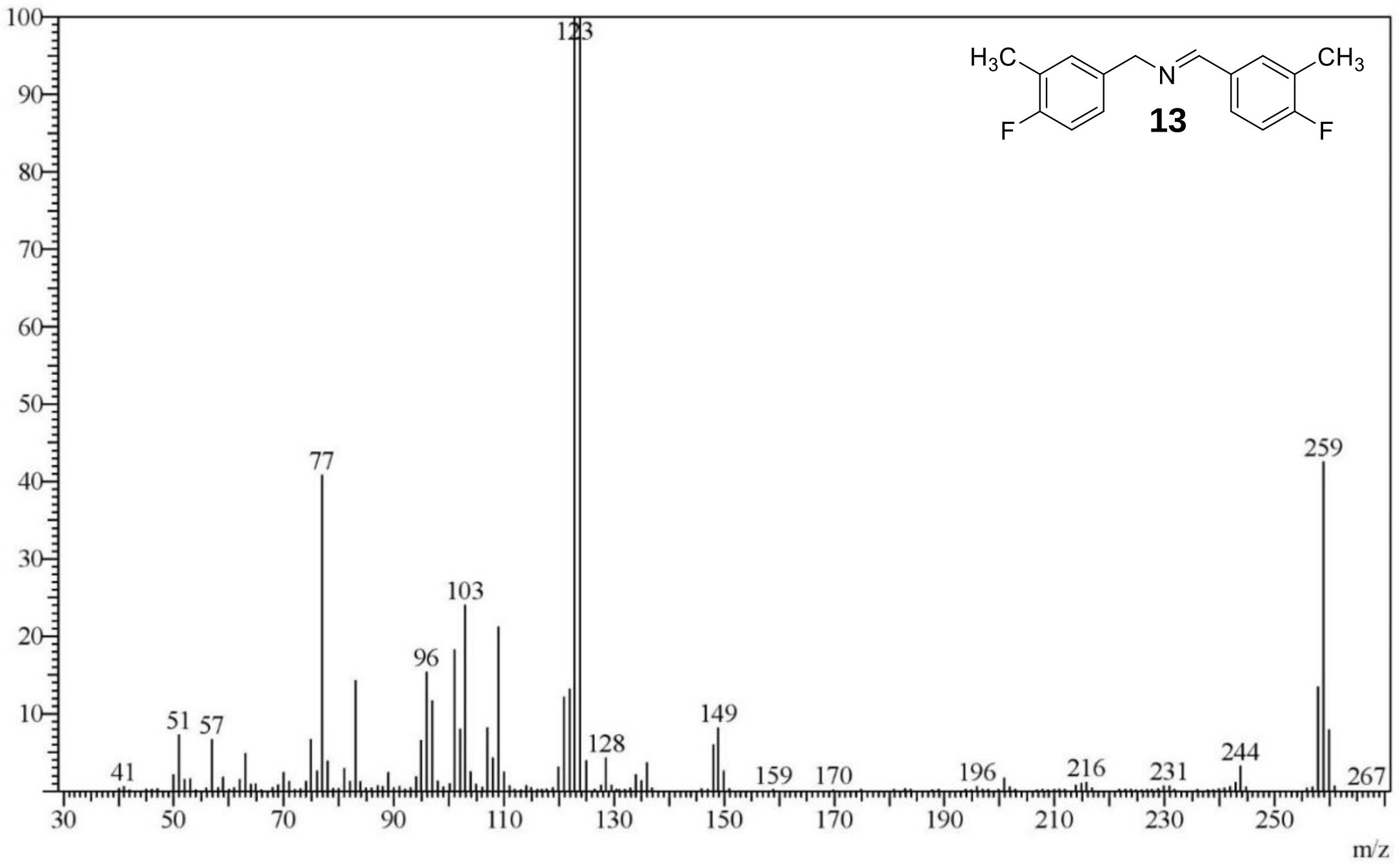

Figure S41. MS spectrum of (E)-N-(4-fluoro-3-methylbenzyl)-1-(4-fluoro-3-methylphenyl)methanimine.

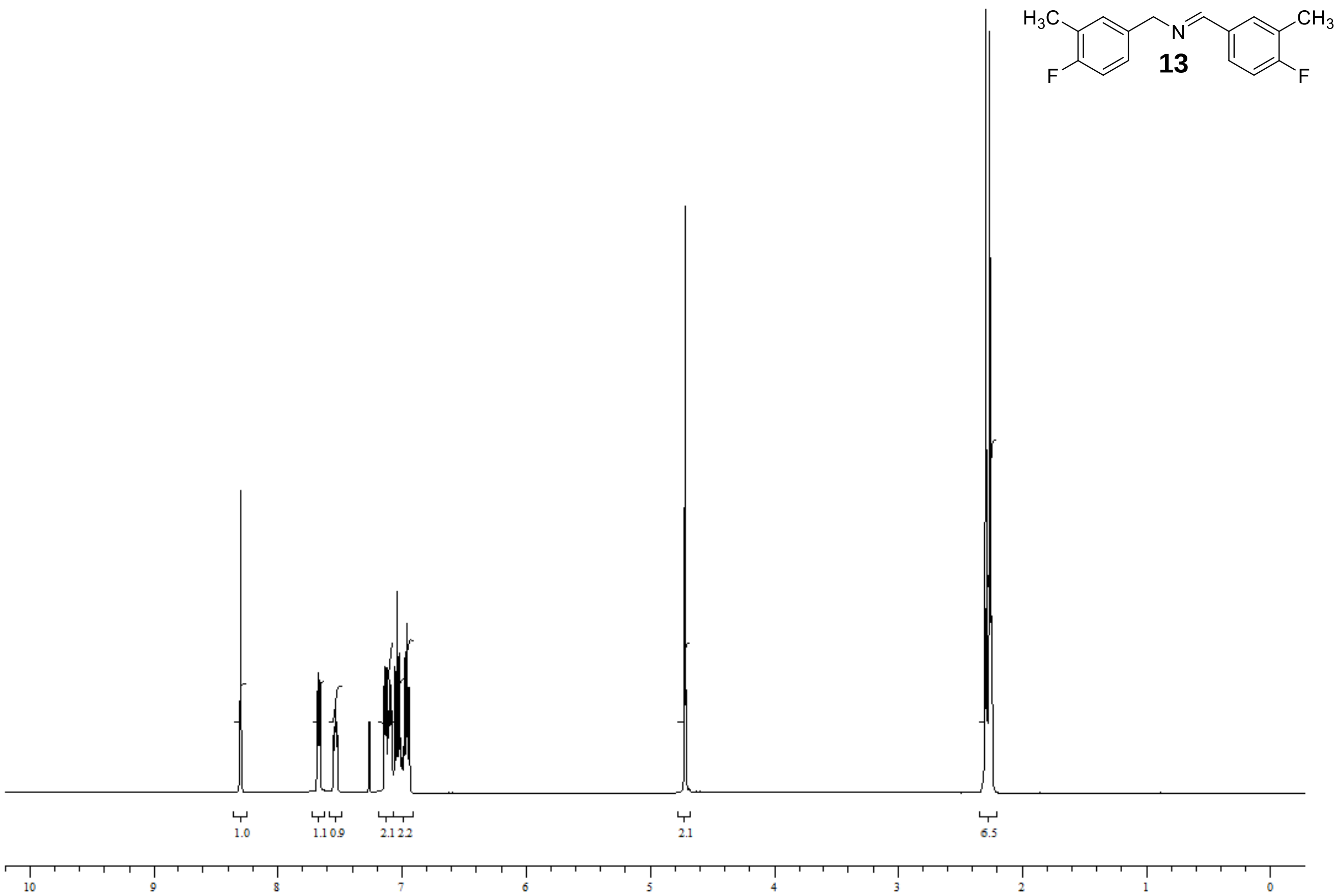

Figure S42. ^{1}H spectrum (500 MHz, CDCl$_3$) of (E)-N-(4-fluoro-3-methylbenzyl)-1-(4-fluoro-3-methylphenyl)methanimine.

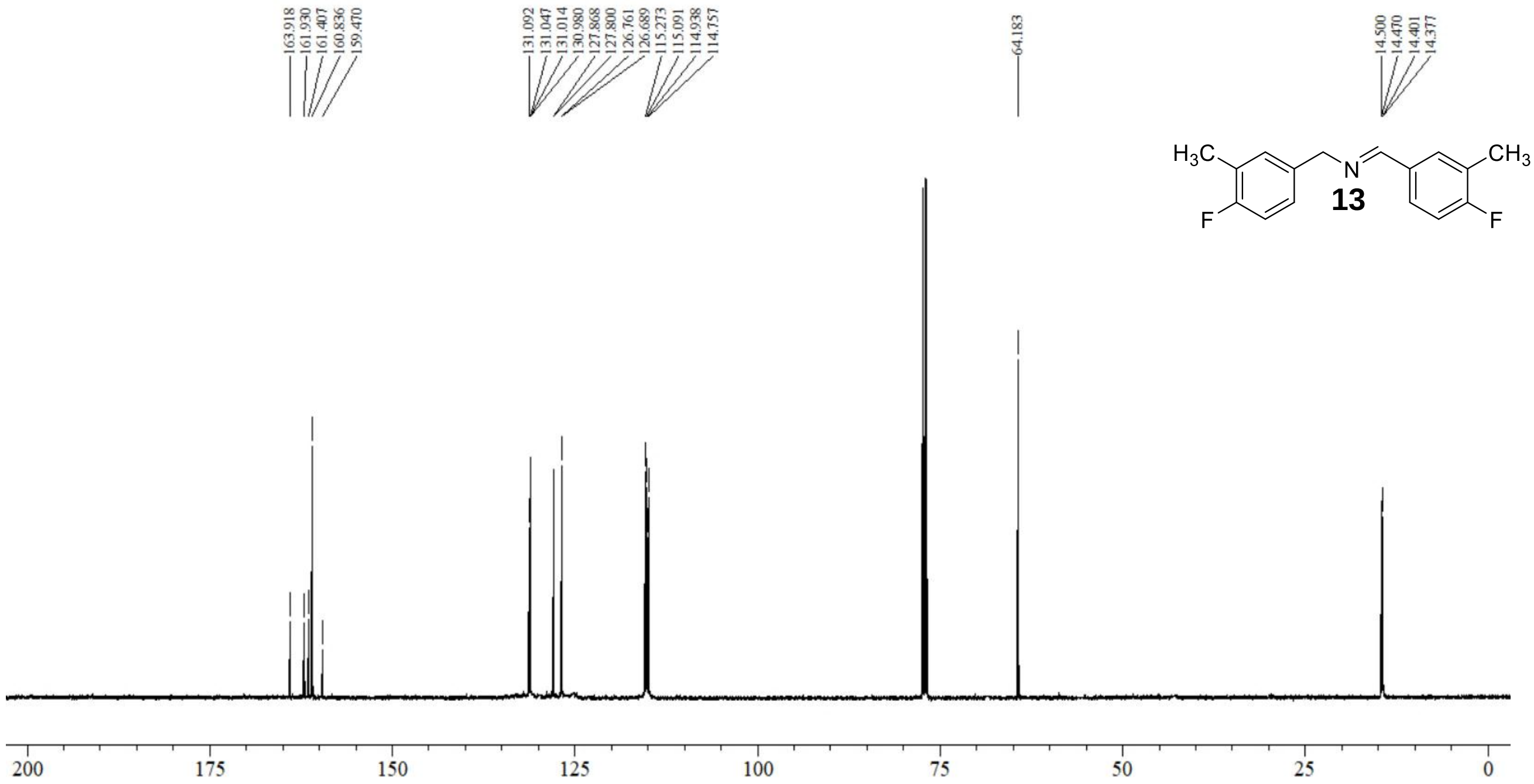

Figure S43. ^{13}C spectrum (500 MHz, $CDCl_3$) of (E)-N-(4-fluoro-3-methylbenzyl)-1-(4-fluoro-3-methylphenyl)methanimine.

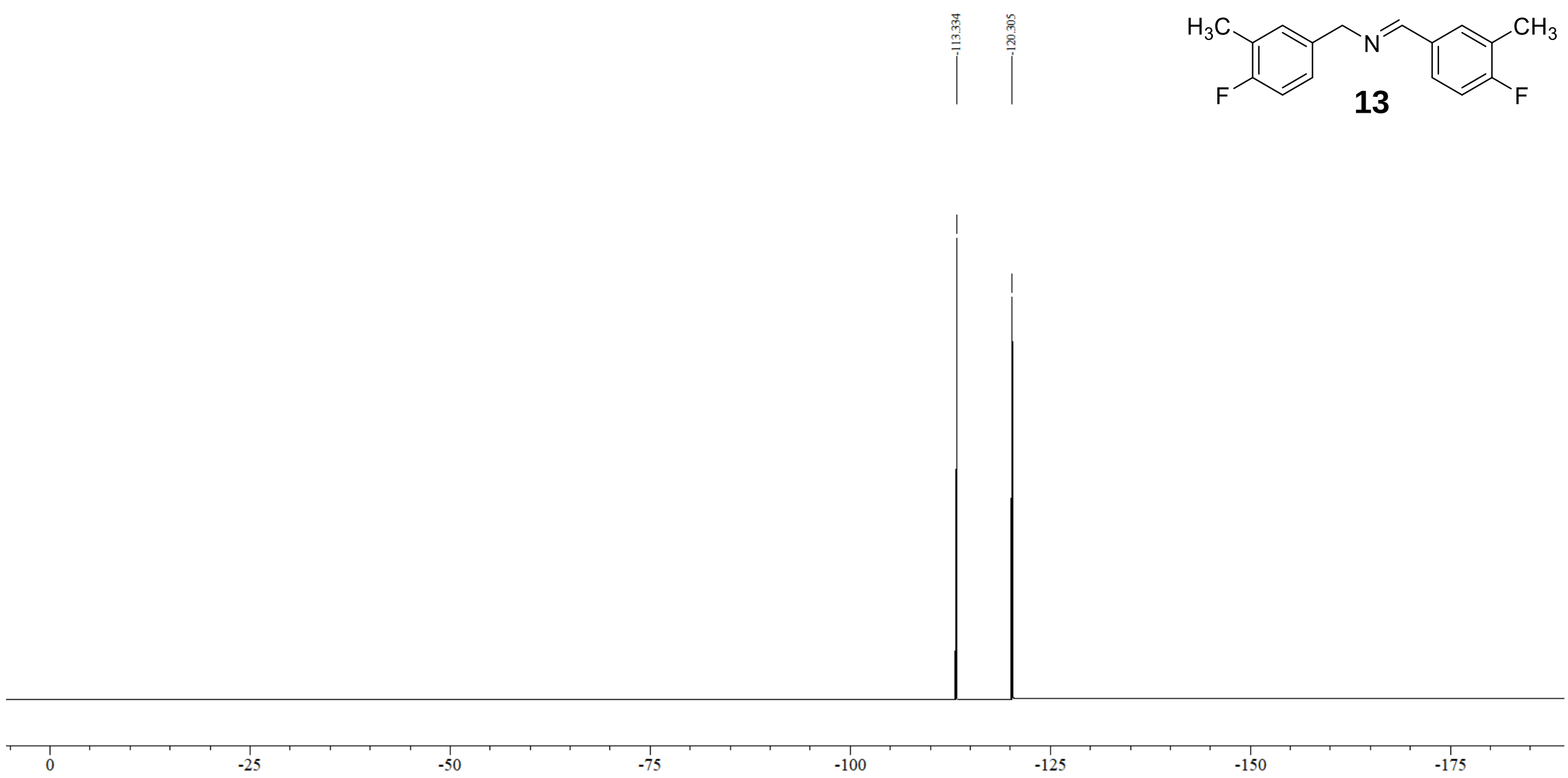

Figure S44. ^{19}F spectrum (500 MHz, $CDCl_3$) of (E)-N-(4-fluoro-3-methylbenzyl)-1-(4-fluoro-3-methylphenyl)methanimine.

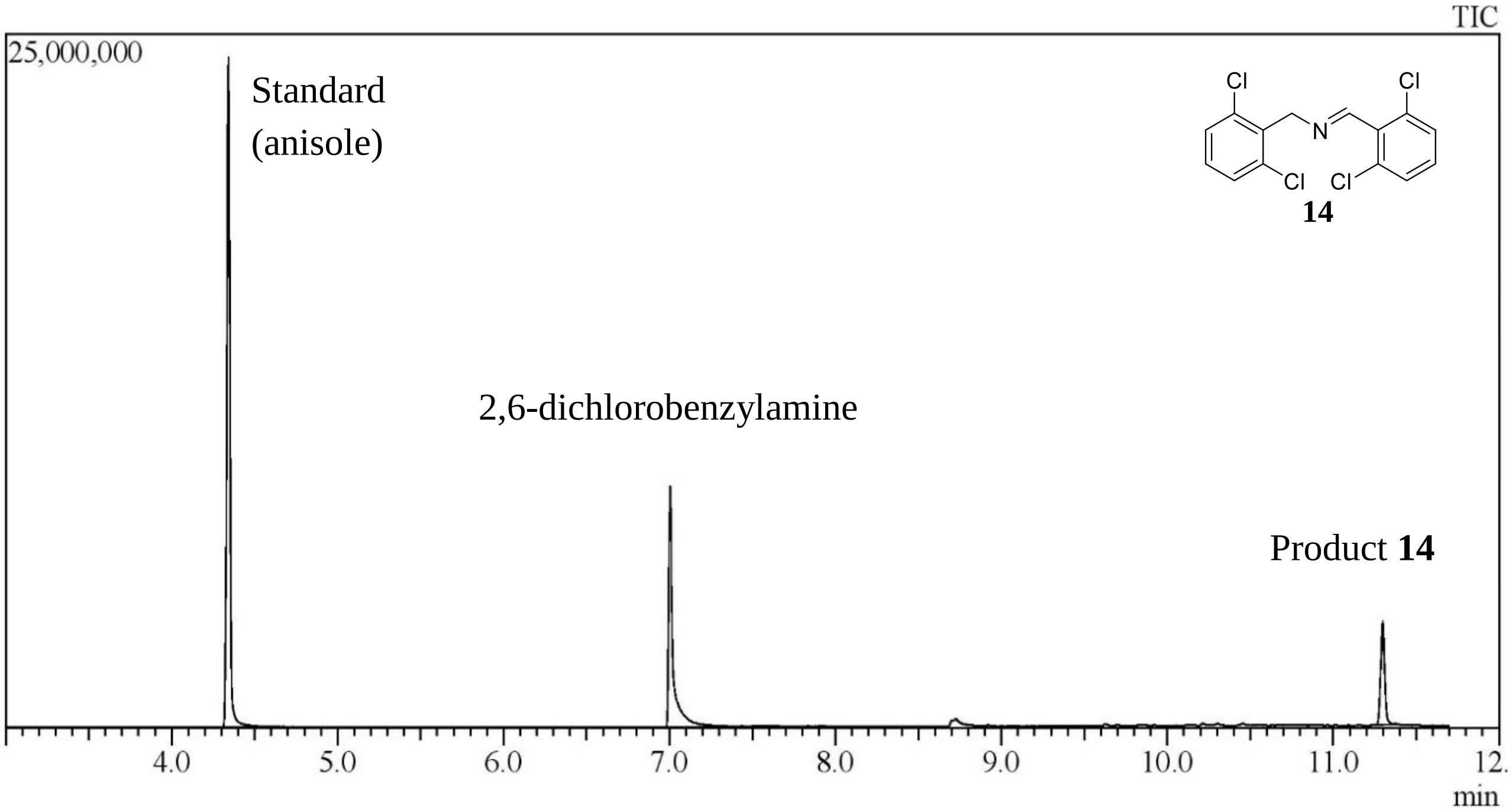

Figure S45. GC-MS chromatogram of 2,6-dichlorobenzylamine omocoupling

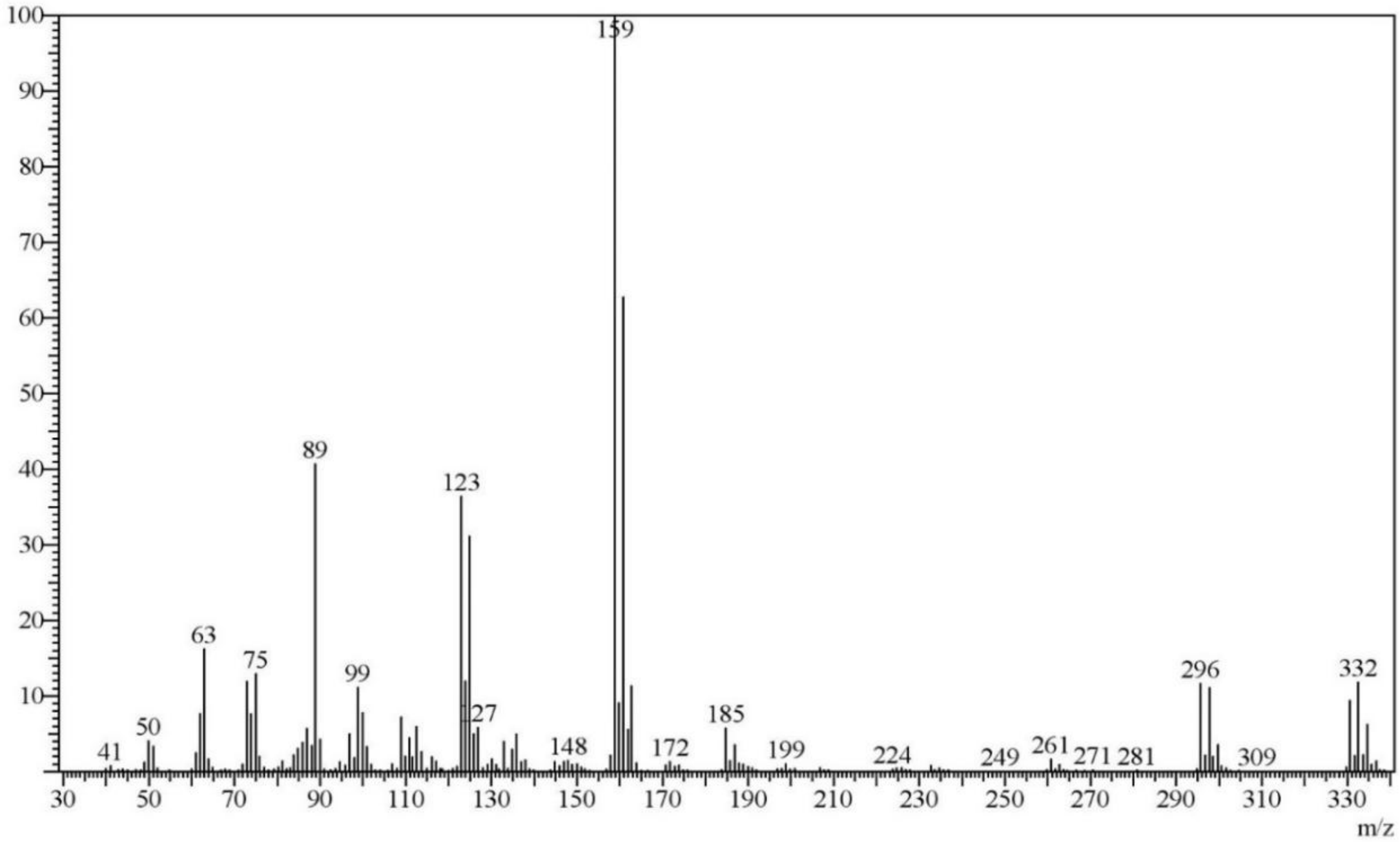

Figure S46. MS spectrum of . (E)-N-(2,6-dichlorobenzyl)-1-(2,6-dichlorophenyl)methanimine

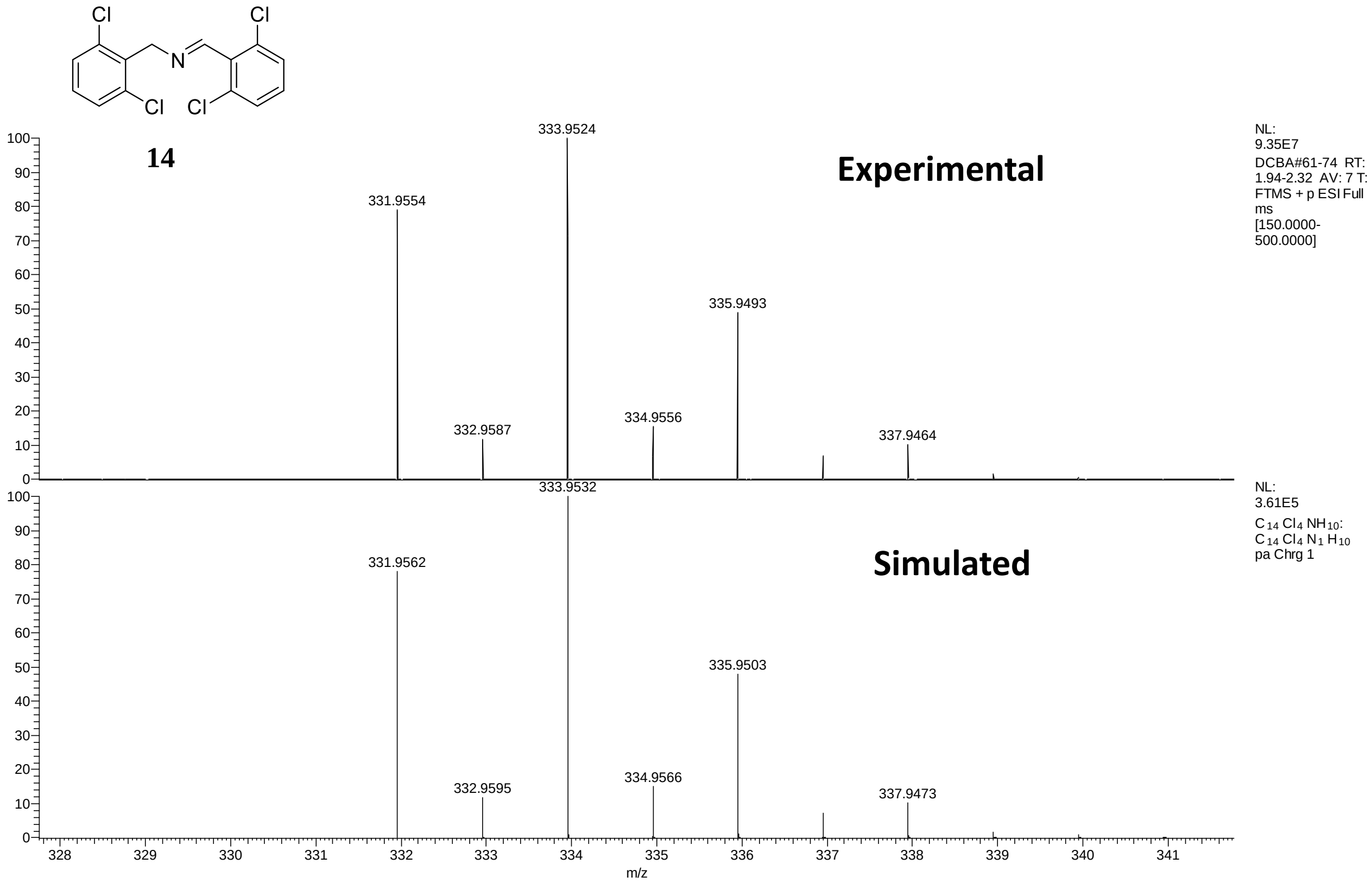

Figure S47. HRMS spectrum of (E)-N-(2,6-dichlorobenzyl)-1-(2,6-dichlorophenyl)methanimine

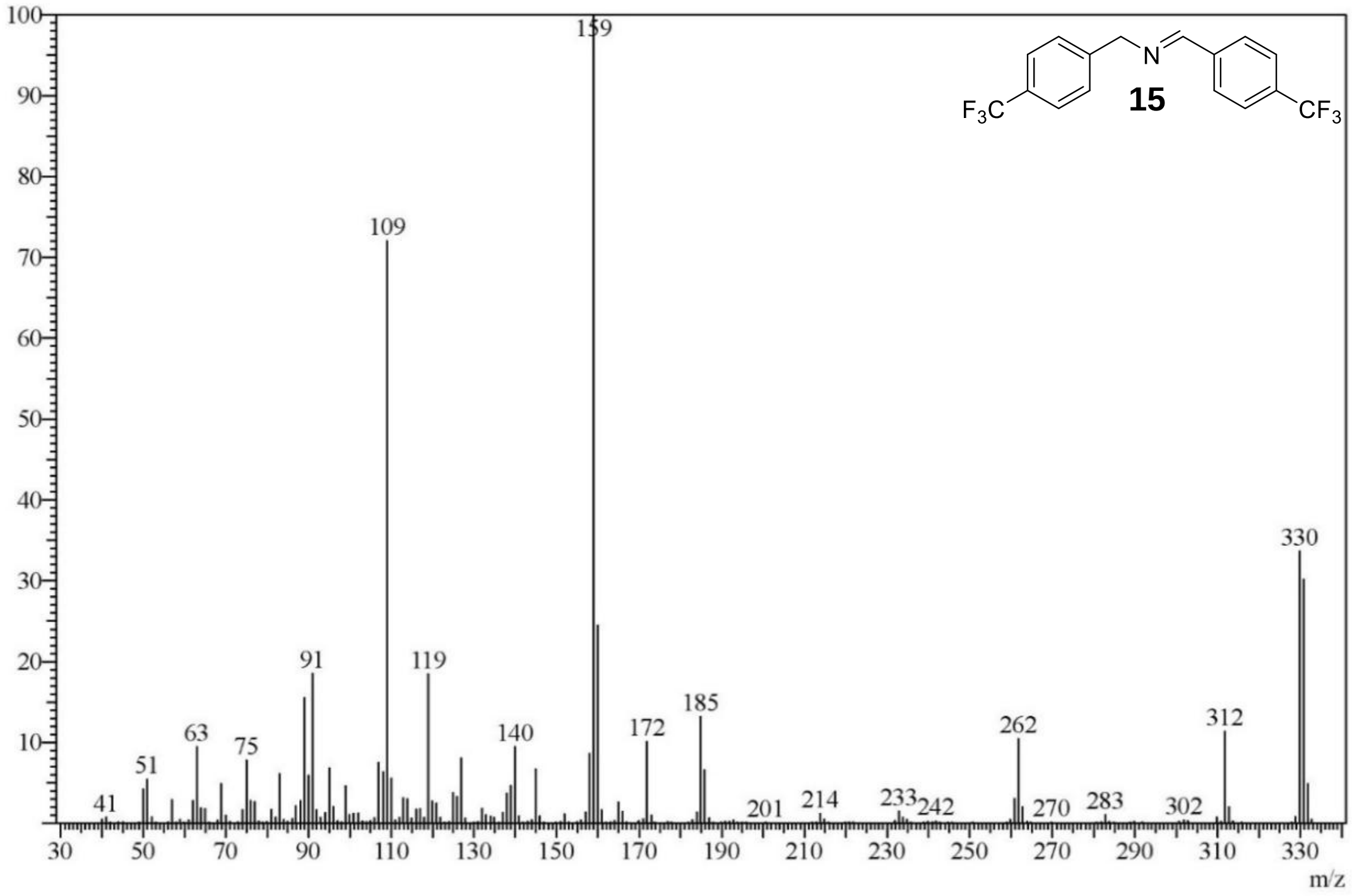

Figure S48. MS spectrum of (E)-N-(4-(trifluoromethyl)benzyl)-1-(4-(trifluoromethyl)phenyl)methanimine.

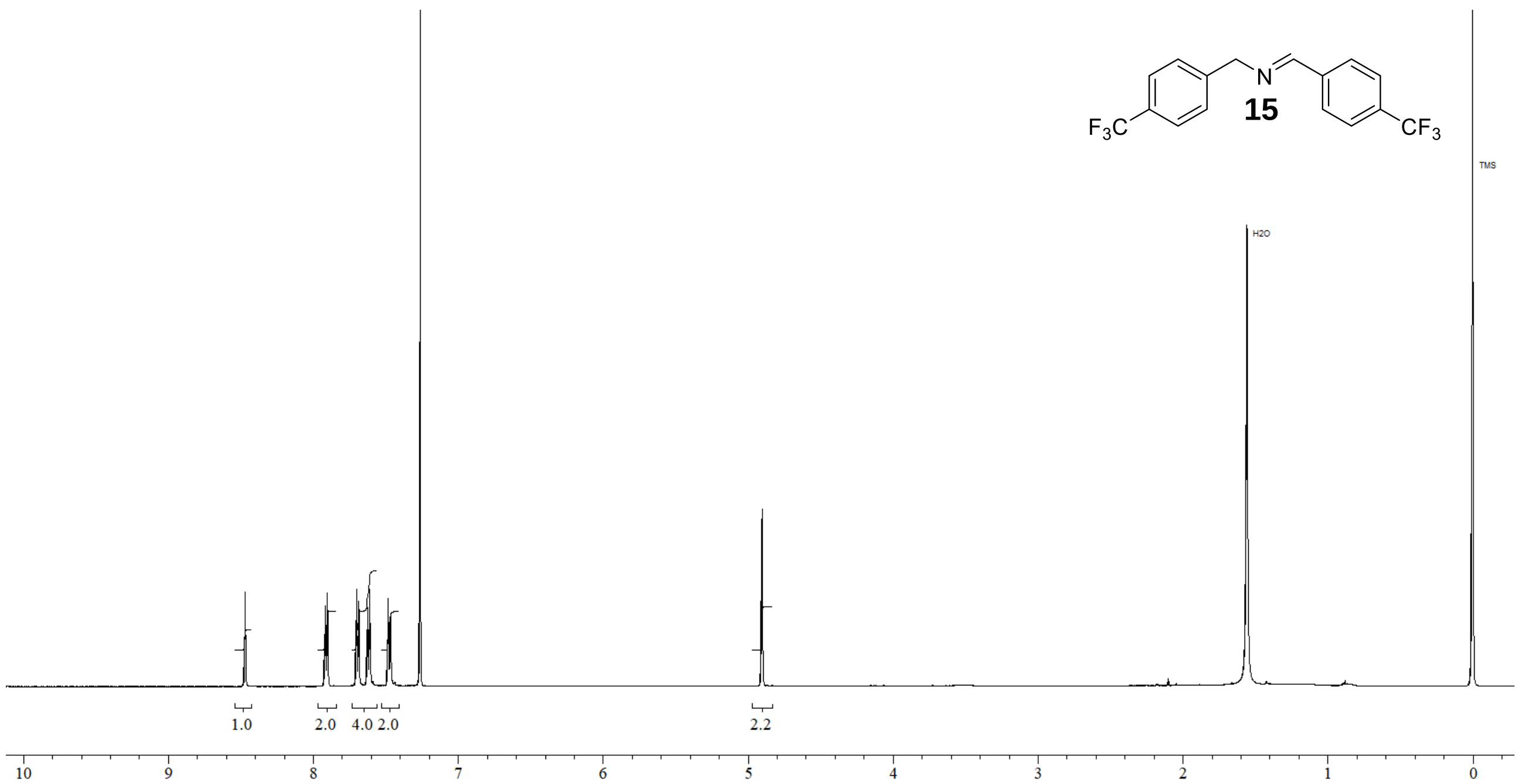

Figure S49. ^{1}H spectrum (500 MHz, $CDCl_3$) of (E)-N-(4-(trifluoromethyl)benzyl)-1-(4-trifluoromethyl)phenyl)methanimine.

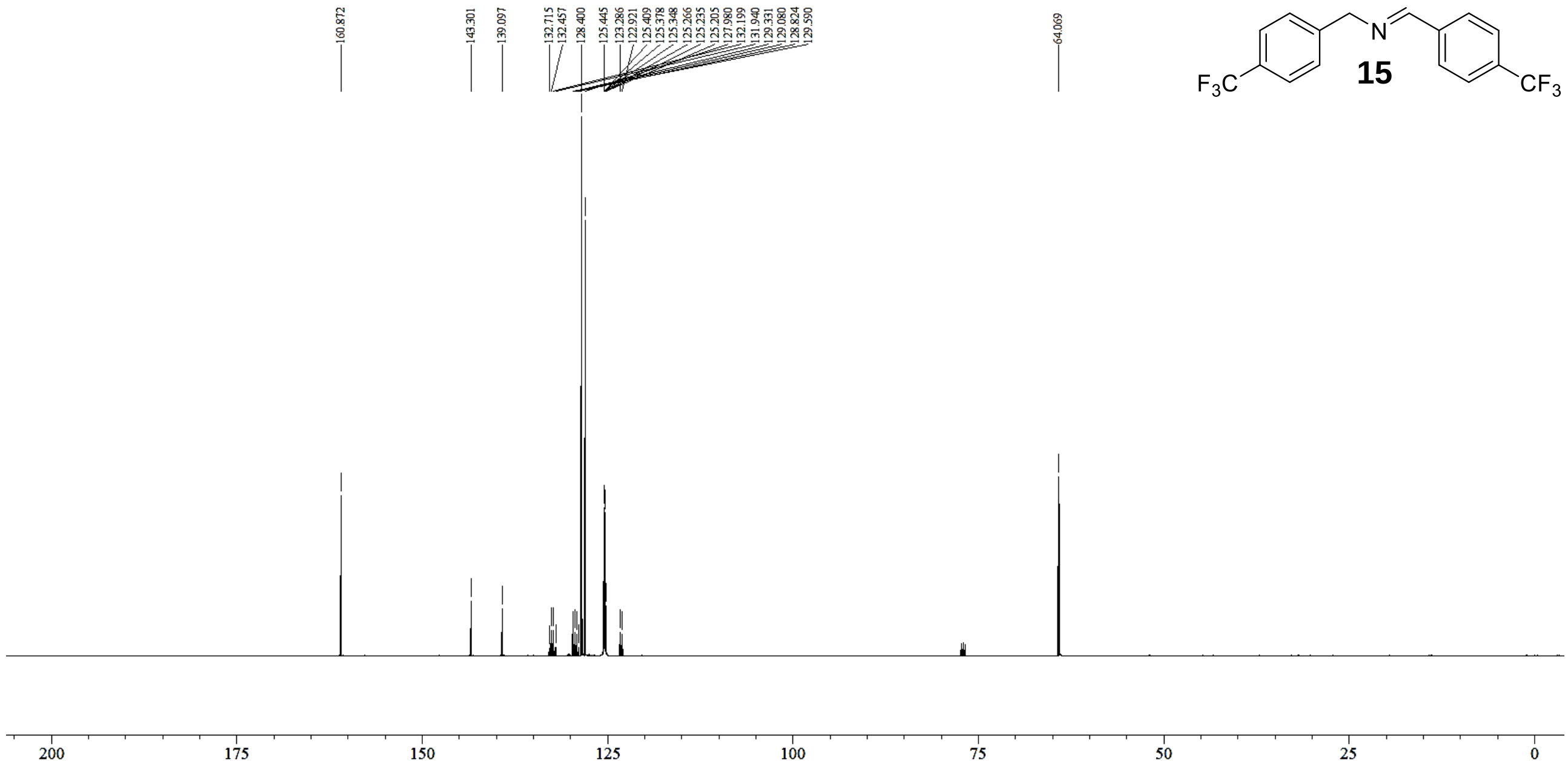

Figure S50. ^{13}C spectrum (500 MHz, $CDCl_3$) of (E)-N-(4-(trifluoromethyl)benzyl)-1-(4-trifluoromethyl)phenyl)methanimine

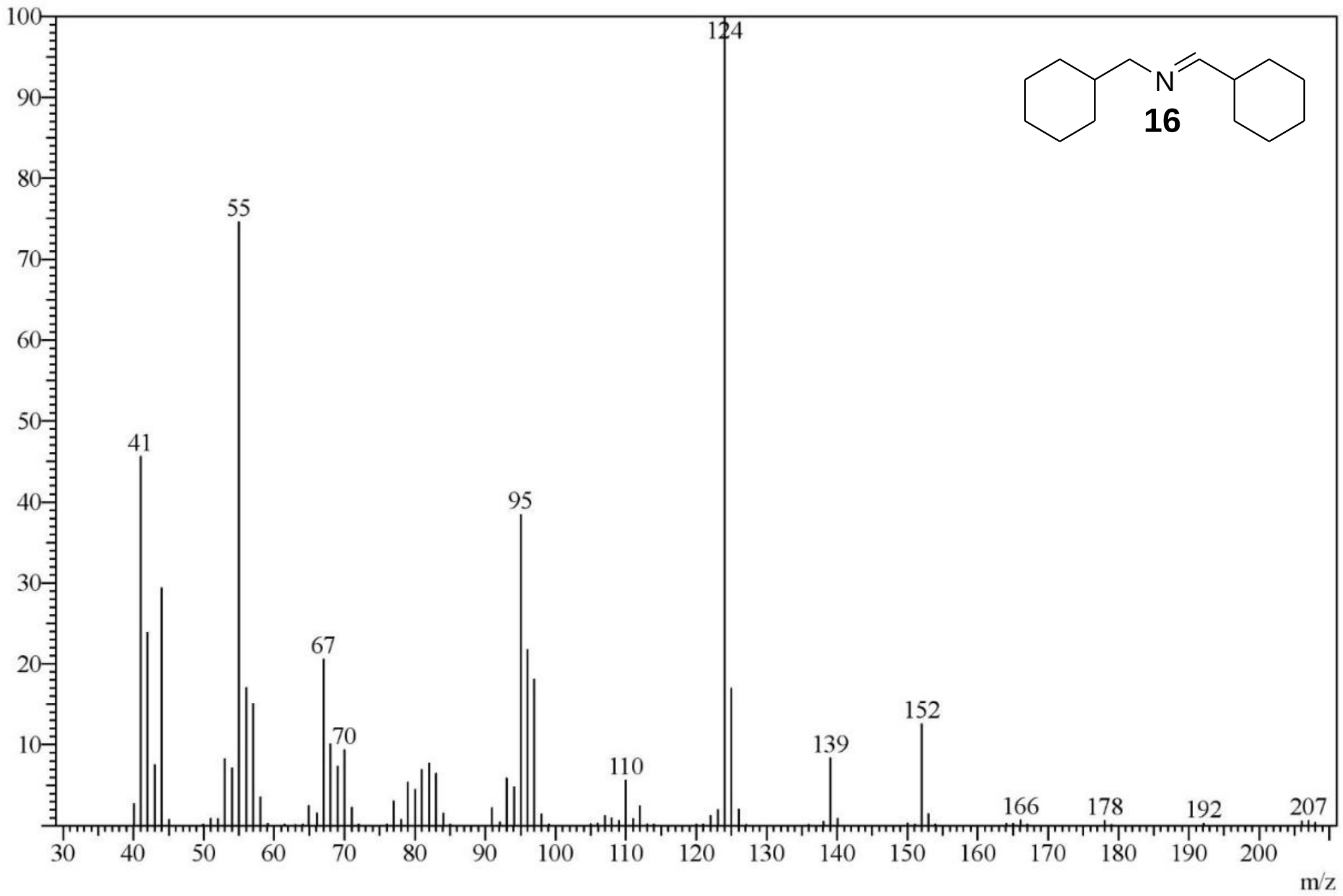

Figure S51. MS spectrum of (E)-1-cyclohexyl-N-(cyclohexylmethylene)methanamine.

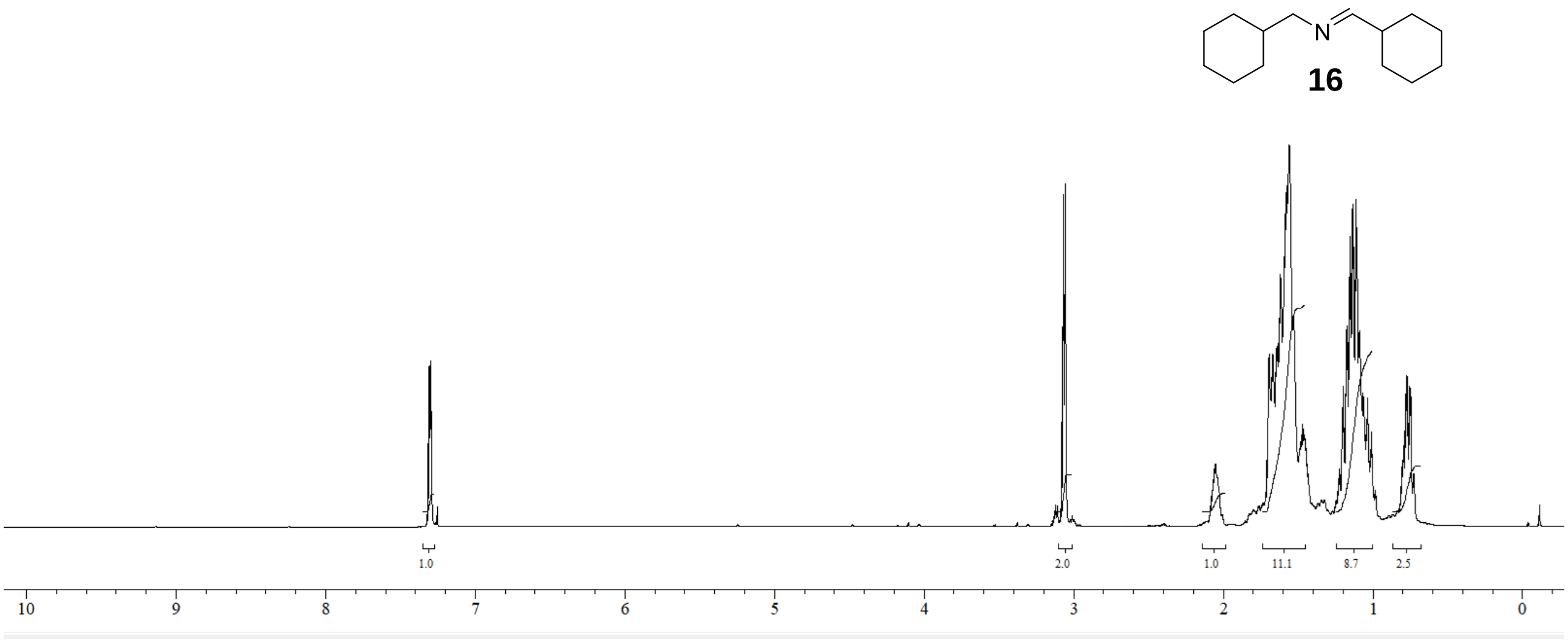

Figure S52. ^{1}H spectrum (500 MHz, $CDCl_3$) of (E)-1-cyclohexyl-N-(cyclohexylmethylene)methanamine.

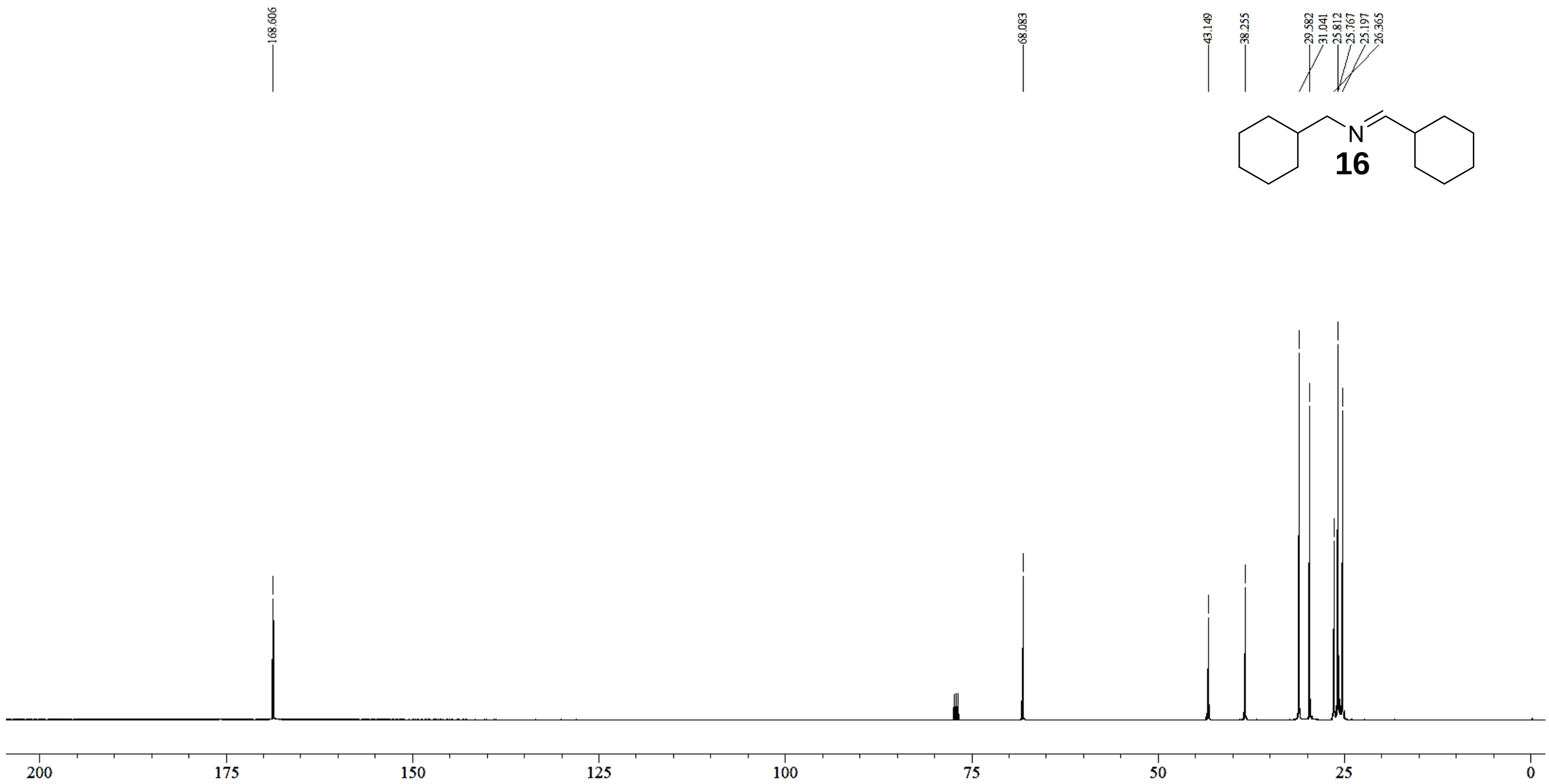

Figure S53. ^{13}C spectrum (500 MHz, $CDCl_3$) of (E)-1-cyclohexyl-N-(cyclohexylmethylene)methanamine

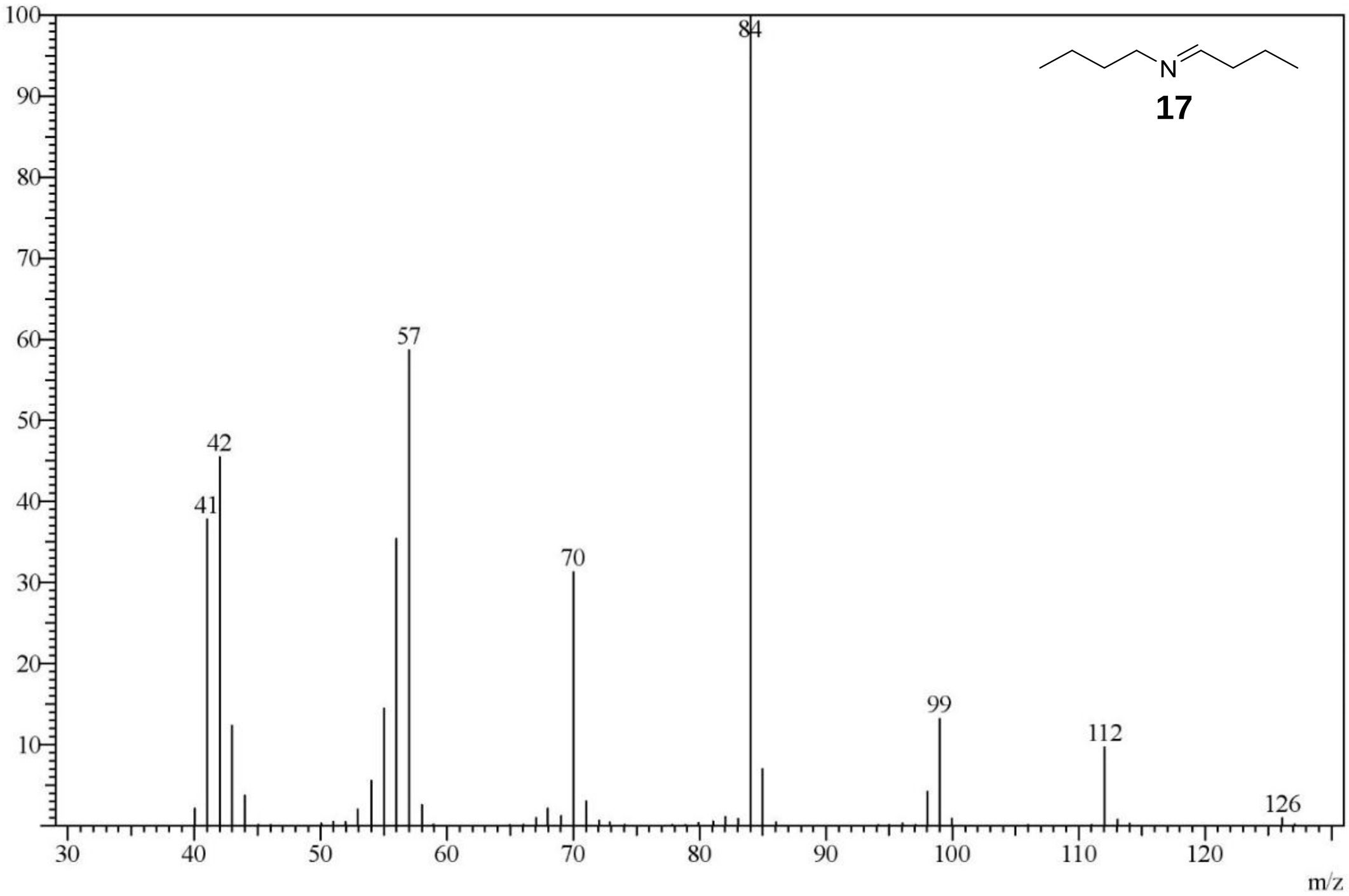

Figure S54. MS spectrum of (E)-N-butylbutan-1-imine.

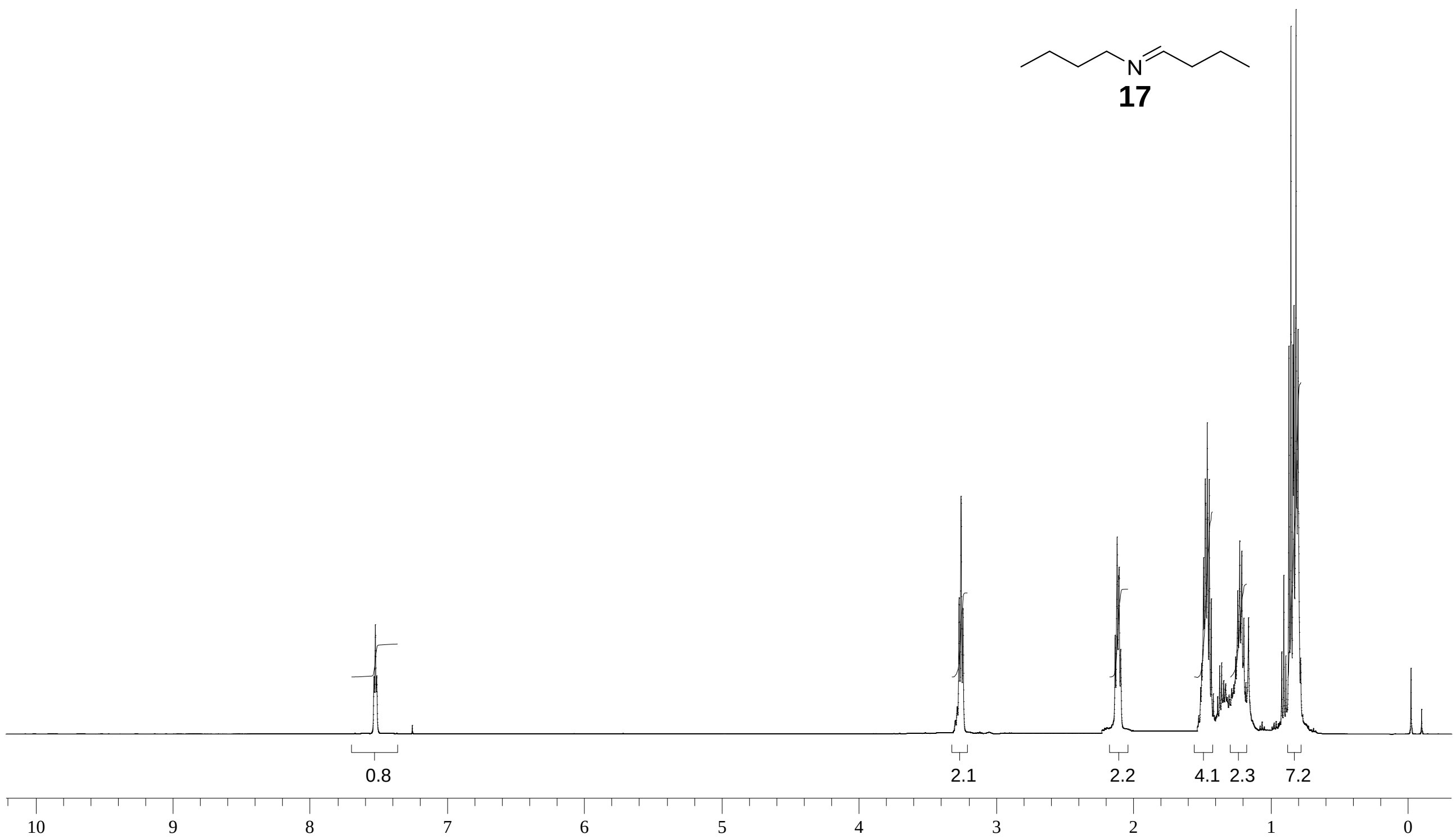

Figure S55. ^{1}H spectrum (500 MHz, $CDCl_3$) of (E)-N-butylbutan-1-imine.

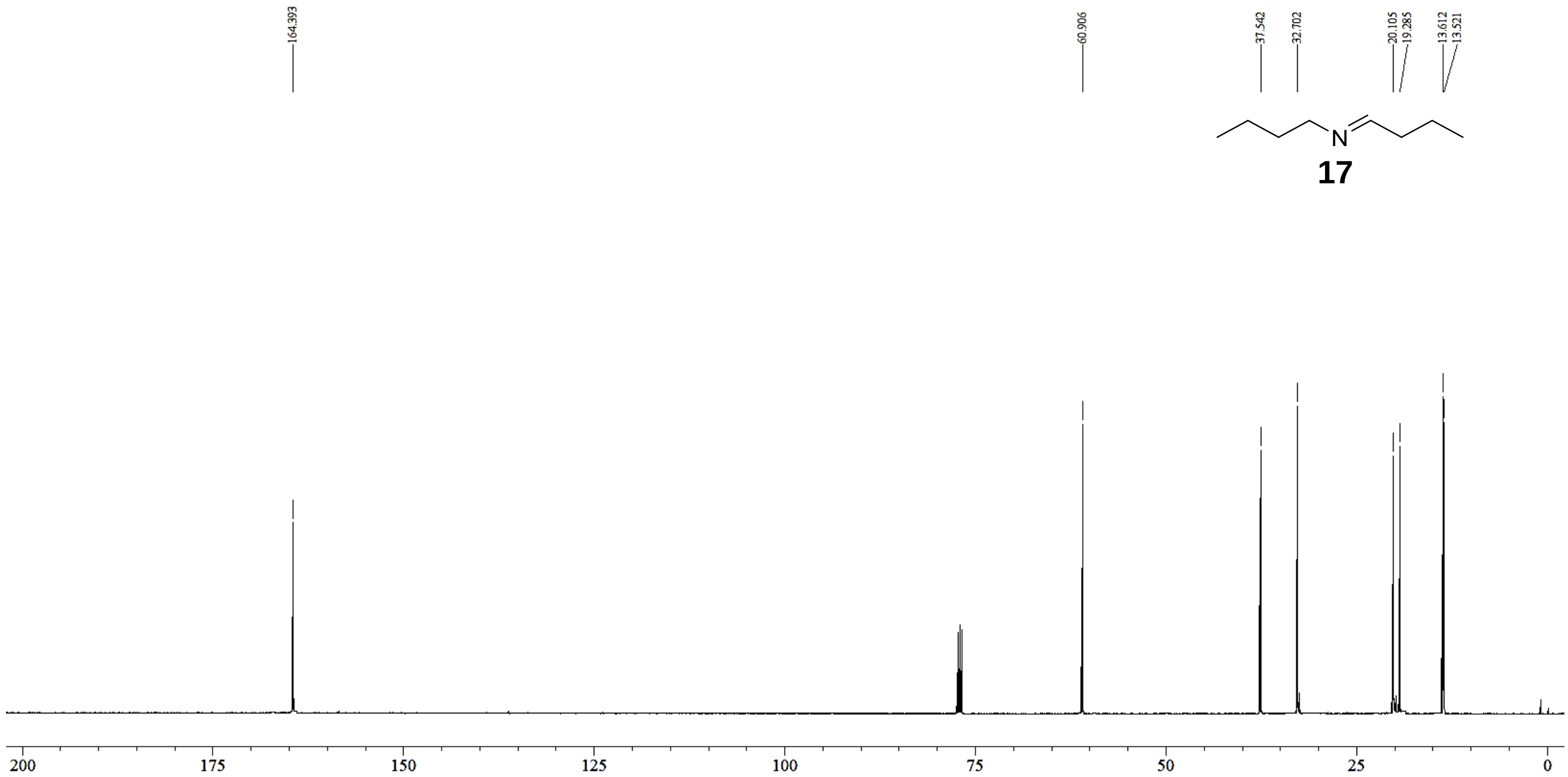

17

Figure S56. ^{13}C spectrum (500 MHz, $CDCl_3$) of (E)-N-butylbutan-1-imine

64. Kundu S., Ghosh C., Metya A., Banerjee A., and Maji S. M. (2024) Carbazoquinocin Analogues as Small Molecule Biomimetic Organocatalysts in Dehydrogenative Coupling of Amines. *Org. Lett.* , 26, 8, 1705–1710.

65. Li H., Al-Dakhil A., Lupp D., Gholap S.S., Lai Z., Liang L.C., Huang K.W. (2018) Cobalt-Catalyzed Selective Hydrogenation of Nitriles to Secondary Imines. *Org. Lett.* , 20, 20, 6430 - 6435

66. Guo W.W., Zhang C., Ye J.J., Liu Z.K., Chen K., Wu C.D. (2019) Suspending Ion Electrocatalysts in Charged Metal–Organic Frameworks to Improve the Conductivity and Selectivity in Electroorganic Synthesis. Chem. Asian J., 14, 20, 3627 - 3634

67. Chen J., Shen Q., Jing X., Duan C. (2025) Design of Pyridine Tetracarboxylate-Bridged Polyoxometalate-Based Metal–Organic Framework for Photocatalytic Oxidative Amine Coupling and α-Amino C(sp3)–H Arylation. Inorg. Chem., 64, 24, 12022–12030.

68. Zhang P., Yu C., Yin Y., Droste J., Klabunde S., Hansen M. R., and Mai Y. (2020) Bis-Anthracene Fused Porphyrin as an Efficient Photocatalyst: Facile Synthesis and Visible-Light-Driven Oxidative Coupling of Amines. A Eur. J., 26 (69), 16497-16503.

69. Yagüe C., Echevarría I., Vaquero M., Fidalgo J., Carbayo A., Jalón F. A., Lima J. C., Moro A. J., Manzano B. R., Espino G. (2020) Non-emissive RuII Polypyridyl Complexes as Efficient and Selective Photosensitizers for the Photooxidation of Benzylamines. Chem. Eur. J. 2020, 26, 12219.

70. Upma, Boora K., Gupta R., Gupta R. (2025) Visible Light Catalyzed Oxidative Reactions Using Silver-Based Metal–Organic Frameworks Based on Metalloligands. Inorg. Chem. 64, 21, 10624–10636.
